# Lifetime burden of prescription medication for insomnia in middle-aged and older adults in the US: a microsimulation study

**DOI:** 10.1016/j.lana.2025.101284

**Published:** 2025-10-24

**Authors:** Hanke Heun-Johnson, Johanna Thunell, Jonathan N. Cloughesy, Jeffrey A. Linder, Stephen D. Persell, Mark D. Sullivan, Bryan Tysinger, Jason N. Doctor

**Affiliations:** a Leonard D. Schaeffer Center for Health Policy & Economics, University of Southern California, Los Angeles, CA, USA; b Sol Price School of Public Policy, University of Southern California, Los Angeles, CA, USA; c Alfred E. Mann School of Pharmacy and Pharmaceutical Sciences, University of Southern California, Los Angeles, CA, USA; d Division of General Internal Medicine, Department of Medicine, Feinberg School of Medicine, Northwestern University, Chicago, IL, USA; e Center for Primary Care Innovation, Institute for Public Health and Medicine, Feinberg School of Medicine, Northwestern University, Chicago, IL, USA; f Psychiatry and Behavioral Sciences, University of Washington, Seattle, WA, USA

**Keywords:** Falls, Cognitive impairment, QALY, Benzodiazepines, Z-drugs, Trazodone

## Abstract

**Background:**

Prescription sleep medications, including z-drugs, benzodiazepines, and trazodone, are commonly used treatments in older adults for insomnia, but have negative consequences related to injuries, cognitive impairment, and quality of life. This study estimates the remaining lifetime burden of sleep medication for US individuals over 50 years of age.

**Methods:**

This study utilized the Future Elderly Model, a state-transition microsimulation based on 1998–2018 data from the Health and Retirement Study, to evaluate health and economic outcomes among individuals over 50 who regularly use prescription medications to help sleep. We simulated the status quo scenario and compared it to a scenario without future sleep medication use.

**Findings:**

Avoiding future sleep medication use in 15.3 million Americans over age 50 regularly using these drugs decreased lifetime incidence of falls by 8.5% (95% CI, 7.3–9.7) cognitive impairment by 2.1% (95% CI, 1.7–2.6), and increased life expectancy with 0.11 years (95% CI, 0.09–0.14). Collectively, eliminating future use could save 1.7 million life years and 1.3 million quality-adjusted life years in this cohort. The net lifetime economic savings was $6.6 K (95% CI: 4.7–8.5, 2024$) per person and $101 billion (95% CI: 72–129) in the US. The largest share is attributed to improved quality of life.

**Interpretation:**

In this microsimulation study, sleep medications in the status quo scenario worsen quantity and quality of life. Deprescribing efforts may improve quality of life for middle-aged and older Americans.

**Funding:**

National Heart, Lung & Blood Institute.

**Research in context**

**Evidence before this study**

We used PubMed and Google Scholar to search for studies published prior to May 2025 that investigated prescription drug use for insomnia and its consequences in older persons. We used search terms “insomnia,” “treatment,” “elderly,” “z-drugs,” “trazodone,” and “benzodiazepines”.” Treatment of insomnia with prescription medications is common, but they can have harmful side effects, lack evidence of long-term effectiveness, and are only recommended for a short period. In older adults, side effects include fractures from falls, cognitive impairment, parasomnias, and dependence. Discontinuation can lead to short-term rebound insomnia (about two weeks), but likely has longer-term health benefits.

**Added value of this study**

Through a dynamic microsimulation of 15.3 million Americans aged 51 and older, this study found that avoiding future prescription sleep medication was associated with a decreased lifetime incidence of falls, cognitive impairment, living in a nursing home, and increased life expectancy and quality-adjusted life years. Study findings demonstrate a lasting net benefit of cessation of sleep medication use.

**Implications of all the available evidence**

Sleep medication use has negative effects in both the short and longer term. Together with prior evidence of these effects, our results support the need to prescribe sleep medications less often and rely on alternatives, such as cognitive behavioral therapy for insomnia (CBT-I). Future research using behavioral economics interventions may offer a pathway toward the reduction of sleep medication use and improve the lives of middle-aged and older Americans.

## Introduction

Insomnia is characterized by having trouble with the initiation and maintenance of sleep and is common in older adults, with up to 50% of persons over age 65 reporting symptoms.^1^, ^2^, ^3^ Older people with insomnia have an increased risk of depression, anxiety, heart disease, hypertension, and cognitive impairment^3^^,^^4^; treating chronic insomnia may reduce risk of negative outcomes and improve quality of life.^3^, ^4^, ^5^, ^6^ Pharmacological treatment, for example with z-drugs (zolpidem, zaleplon, eszopiclone), benzodiazepines (BZDs), or trazodone, is only recommended for a short period because of serious harmful side effects and lack of evidence of their long-term effectiveness.^7^, ^8^, ^9^, ^10^, ^11^ In older adults, these side effects include fractures from falls, cognitive impairment, parasomnias, and dependence.^12^, ^13^, ^14^, ^15^, ^16^, ^17^, ^18^ Citing these adverse effects, benzodiazepines and z-drugs have been placed on the Beers list for potentially inappropriate medication use, and clinical practice guidelines from multiple medical societies (e.g. American Academy of Sleep Medicine) discourage their use.^7^^,^^15^^,^^19^ Despite safety concerns, an estimated 12% of older adults utilize sleep medications to treat insomnia; use increases with age and is more likely among non-Hispanic white persons and women.^20^, ^21^, ^22^ While use of some sleep medications (e.g. zolpidem) decreased somewhat in recent years, overall use of these drugs has been relatively steady.^22^, ^23^, ^24^

Deprescribing of sleep medications can be effective both in the short and long term, particularly when coupled with non-pharmacological treatments for insomnia (e.g. psychotherapy or cognitive behavioral therapy for insomnia [CBT-I]).^25^ Information on the lifetime burden of sleep medications in the United States—including health care costs, quality of life, and earnings—is not currently available but would help guide the prioritization of efforts to deprescribe sleep medication or reduce their initiation. Dynamic microsimulation can estimate lifetime projections of outcomes (e.g. falls, cognitive decline, nursing home) and medical costs among older adults taking sleep medications as well as changes in outcomes after eliminating these medications. In this study, we use the Future Elderly Model (FEM), which is based on nationally-representative Health and Retirement Study (HRS) data, to quantify lifetime burden of sleep medication use compared to the counterfactual of individuals no longer taking any sleep medication.

## Methods

This study was approved by the institutional review board at the University of Southern California with a waiver of informed consent, and followed the Consolidated Health Economic Evaluation Reporting Standards (CHEERS).^26^

### Model development

The Future Elderly Model (FEM) is a publicly-available, dynamic microsimulation model that has been used to project future health and economic outcomes in the US population.^26^, ^27^, ^28^, ^29^ The FEM relies on longitudinal trajectories from the nationally-representative, longitudinal Health and Retirement Study (HRS)^30^ of adults ages 51 years and older in 1998–2018. Other data, such as from the Medical Expenditures Panel Survey (MEPS) and Medicare Current Beneficiary Survey (MCBS) are used to calibrate and extend FEM outcomes. FEM estimates common disease incidence (e.g. heart disease, diabetes), changes in health risk factors (e.g. smoking, BMI), quality and quantity of life (e.g. mortality, QALYs), functional limitations, nursing home status, demographic and socioeconomic characteristics, and more. The FEM can be used to estimate the overall impact of diseases or potentially harmful treatment on health and economic consequences, and can also accommodate hypothetical counterfactual scenarios to estimate burden.

**Fig. 1:**
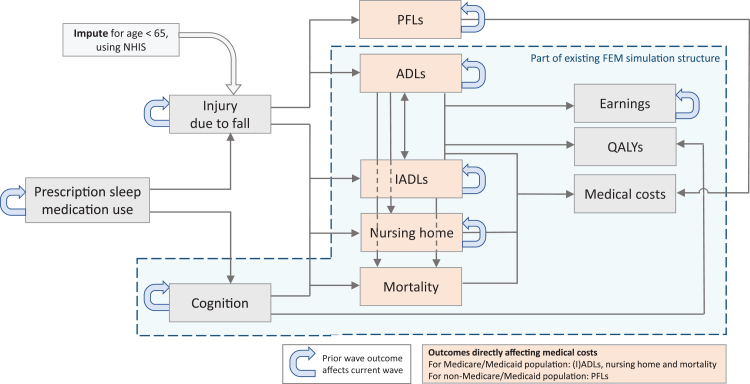
The FEM simulation framework was expanded to include additional models related to (effects of) prescription sleep medications. The existing structure is indicated with a light-blue background. Grey arrows indicate which outcomes are affecting each other and the direction of the relationship. Blue self-referencing arrows indicate whether the prior status of a simulant affects the current status (two years later). This overview is a reduced schematic; transition models include other variables such as demographic characteristics, health behaviors and health conditions. The FEM also includes other models not shown here. See Supplementary data 2 and 3 for all specifications and coefficients. ADLs: limitations in activities of daily living; IADLs: limitations in instrumental activities of daily living; NHIS: National Health Interview Survey; PFLs: limitations in physical functioning; QALYs: quality-adjusted life years, based on Health Utilities Index Mark 3.

For the current study, we developed models with HRS data from 2008 to 2018 to simulate effects of sleep medication use (Fig. 1). Model extensions were validated using calculations of area under the receiver-operator characteristic curve (ROC) (Supplementary data 1, Figures S1 and S2).

Studies show that sleep medication use increases the probability of injuries due to falling and cognitive impairment.^12^, ^13^, ^14^ We included prevalent use of sleep medications in transition models that predicted cognitive impairment and fall injuries and their effect on future functional limitations, living in a nursing home, mortality, medical costs, earnings, and quality-adjusted life years (QALYs). Refer to Supplementary data 2 for model specifications.

### Simulation cohort and outcomes

The starting cohort consisted of 2016 HRS respondents who indicated regularly taking prescription medication to help sleep. We compared simulation outcomes under status quo and counterfactual scenarios for each respondent all else equal, where all HRS respondents currently using sleep medication would discontinue usage for the rest of their lives. The difference between these scenarios summed across all individuals represented the burden of sleep medications for this cohort. We report results for the whole cohort and stratified by age, sex, self-reported race-ethnicity, and educational attainment. Race-ethnicity categories were defined as Hispanic, non-Hispanic Black, non-Hispanic White, and non-Hispanic “other”. Because of a lower sample size, non-Hispanic “other” was combined with non-Hispanic White. Educational attainment was defined as less than high school, high school graduation (including GED), and some college or more. The analysis of a specific subgroup of interest, those who were predicted to regularly use z-drugs, was included as a sensitivity analysis (Supplementary data 1, Section 2). Another sensitivity analysis includes a measure of insomnia in estimation models (Supplementary data 1, Section 3). Simulation outcomes were expressed as a comparison between all remaining life years. For several outcomes (e.g. medical costs), we additionally report only those years when respondents are alive in both scenarios.

*Limitations in (Instrumental) Activities of Daily Living ((I)ADLs)* are indicators of functional limitations due to health, cognitive, or mobility impairments (Supplementary data 1, Section 4). The simulation transitioned (I)ADL variables as categorical variables with the number of ADLs (0, 1, 2, or 3+) and IADLs (0, 1, or 2+) in ordered probit models.

*Limitations in Physical Functioning (PFLs).* We created another measure to identify common mobility issues not captured by ADLs that could impact quality of life and affect medical costs (Supplementary data 1, Section 4). This variable was implemented as a categorical variable with the number of PFLs (0, 1, or 2+), estimating PFL status with an ordered probit model.

*Injury due to fall* data were available for HRS respondents 65 and over. This variable captures fall injuries that are serious enough to need medical treatment. We imputed this variable for ages 51–64 using data from the National Health Interview Survey (NHIS) (Supplementary data 1 and 2, Section 5). In the simulation this variable was transitioned using a probit model.

*Nursing home* status was derived from RAND HRS^31^ data, and was transitioned using a probit model.

*Cognitive impairment* was measured using the Telephone Interview for Cognitive Status (TICS) by HRS, an assessment with a maximum of 27 points, based on immediate and delayed recall, backwards count, and serial 7s. While we aimed to report on dynamic cognitive functioning and not neurodegenerative disease states, we utilized a categorical transition model (ordered probit) to assess cognitive functioning in the simulation, with a score of 0–6 classified as cognitive functioning similar to someone with dementia, 7–11 as someone with mild cognitive impairment, and 12–27 as someone with healthy cognitive functioning.^32^ Adults could transition between cognitive impairment categories over time without restrictions.

*Quality-Adjusted Life Years (QALYs)* were calculated using the Health Utilities Index Mark 3 (HUI3) score, which consists of several health-related measures, including cognition.^33^^,^^34^ QALYs were estimated using an ordinary least squares (OLS) regression model, valued at $150,000 (in 2024$) per additional QALY and discounted for years after the start.^35^, ^36^, ^37^

*Earnings* were calculated as the sum of the respondent's wage/salary income, bonuses/overtime pay/commissions/tips, second job or military reserve earnings, and professional practice or trade income (Supplementary data 3).

*Medical Costs* models were based on MCBS data for Medicare population and MEPS data for non-Medicare, non-institutionalized population ages 51–64 (Supplementary data 1, Section 4 and Supplementary data 3). Functional limitations were represented by limitations in (I)ADLs in the MCBS model, and PFLs in the MEPS model. In the simulation, medical costs were estimated using OLS models.

### Burden calculation

The burden estimate of sleep medications was calculated as the difference between the status quo and counterfactual (where simulants no longer take any sleep medications) value of QALYs, earnings, and medical costs. Burden calculations were calculated over all life years (including differences due to changes in life expectancy). All dollar values were discounted 3% after the start of the simulation and expressed in 2024 dollars.

### Uncertainty and statistical analyses

For baseline characteristics of the starting cohort, the standard error of the mean was used to create 95% confidence intervals, calculated with Stata's svy prefix for survey analysis with weights, strata and primary sampling units.

Mean values for all simulation outcomes were created by averaging outcomes from 75 Monte Carlo repetitions. Uncertainty around the mean was evaluated through a non-parametric bootstrap method, involving 50 resamplings of the survey observations that were used in re-estimating the transition models 50 times for the simulation. The starting cohort, which remained the same, was then simulated using these 50 sets of models, with 75 repetitions each. Outcomes were averaged within each repetition and each bootstrap, and bootstrap averages were used to construct 95% confidence intervals around the simulation means. Data preparation, analyses and simulations were performed using SAS 9.4, Stata MP 17.0, and C++.

### Role of the funding source

The funding sources had no role in collection, analysis, and interpretation of the data, study design, or decision to publish.

## Results

### Cohort description

The simulation cohort included people over age 50 who were taking prescription sleep medication at the start of the simulation, representing approximately 15.3 million Americans, or 13.4% of the reweighted HRS cohort over age 50. At baseline, people using sleep medication were on average slightly older (65.7 years_sleepRx_ vs 64.1 years_no sleepRx_), less often male (37.7%_sleepRx_ vs 48.0%_no sleepRx_), less educated (18.7%_sleepRx_ did not complete high school vs 11.4%_no sleepRx_), with higher prevalence of all reported health conditions and functional limitations, compared to those who do not use sleep medication (Table 1). In addition, they more often had a lower cognitive score (30.5%_sleepRx_ had TICS score <12 vs 17.3%_no sleepRx_), lived in a nursing home (5.8%_sleepRx_ vs 1.7%_no sleepRx_), and their yearly average medical costs were higher ($27,195_sleepRx_ vs $16,556_no sleepRx_).

**Table 1:** Demographic and health characteristics of the simulation cohort (in “Prescription sleep medication” column).

	No prescription sleep medication	Prescription sleep medication
Sample size, N	15,448	2728
Sample size weighted, N	98,560,611	15,292,254
Age, mean	64.1 (63.7–64.5)	65.7 (64.9–66.4)
Female, %	52.0 (51.2–52.8)	62.3 (60.3–64.4)
Male, %	48.0 (47.2–48.8)	37.7 (35.6–39.7)
Black, %	10.4 (9.3–11.4)	10.8 (9.2–12.4)
Hispanic, %	9.8 (8.0–11.6)	9.7 (7.5–12.0)
White, %	79.8 (77.9–81.7)	79.5 (76.8–82.2)
Educational attainment, %		
Less than high school	11.4 (10.2–12.5)	18.7 (16.4–20.9)
High school graduate	31.3 (29.9–32.7)	31.7 (29.4–34.1)
Some college or more	57.3 (55.5–59.2)	49.6 (46.6–52.6)
BMI, kg/m^2^	28.8 (28.6–28.9)	28.9 (28.5–29.3)
Health conditions (ever), %		
High blood pressure	55.5 (54.2–56.7)	68.8 (66.3–71.2)
Diabetes	23.0 (22.1–23.9)	30.9 (28.5–33.3)
Heart disease	20.6 (19.6–21.5)	34.6 (32.1–37.2)
Myocardial infarction	7.7 (7.2–8.1)	15.6 (13.8–17.4)
Lung disease	8.9 (8.4–9.4)	20.9 (18.6–23.2)
Stroke	7.3 (6.7–7.9)	15.6 (14.0–17.2)
Injured from fall (in last 2 yrs), %	8.2 (7.6–8.9)	15.1 (13.5–16.7)
TICS cognitive score <12, %	17.3 (16.3–18.3)	30.5 (28.2–32.9)
Any limitations in ADLs, %	14.4 (13.6–15.2)	37.9 (35.4–40.5)
Any limitations in IADLs, %	11.8 (11.0–12.6)	35.7 (33.0–38.4)
Any PFLs, %	37.2 (35.9–38.4)	70.1 (67.6–72.7)
Living in nursing home, %	1.7 (1.5–1.9)	5.8 (4.8–6.7)
Average medical costs (2024$)	16,556 (16,166–16,946)	27,195 (26,241–28,149)

This cohort included respondents over age 50 who indicated regularly using prescription medication to help sleep in the Health and Retirement Survey, at simulation baseline in 2016. The values in parentheses indicate the 95% confidence intervals based on survey-weighted standard errors.

ADLs: activities of daily living; BMI: body mass index; IADLs instrumental activities of daily living; PFLs: physical functioning limitations; TICS: Telephone Interview for Cognitive Status.

### Cohort simulation—status quo vs eliminating future Sleep Medication use

To predict remaining lifetime outcomes, we simulated the baseline cohort until every person died (Table 2, Column 1). Among our starting cohort, sleep medication was used for approximately half of the rest of their lifetime (51.7%) under the status quo (approximately 9.4 years on average). Injuries due to falls serious enough to need medical attention occurred in a large fraction of the cohort (60.8%). In the counterfactual scenario, eliminating future use of sleep medications reduced this by 8.5% (95% CI: 7.3–9.7) to 55.6%. The majority (75.5%) also experienced cognitive impairment at some point in their life, and the average number of years with cognitive impairment was 5.6 years in the baseline simulation. Eliminating sleep medications reduced lifetime incidence of cognitive impairment by 2.1% (95% CI: 1.7–2.6) and duration by 5.0% (95% CI: 4.1–5.9), or approximately 100 days. These improvements affected other outcomes: the duration of living in a nursing home was reduced by 15 days or 3.0% (95% CI: 2.3–3.6). The duration of living with any limitations in ADLs was reduced by 11 days (0.5%; 95% CI: 0.4–0.7) and in IADLs by 22 days (1.2%; 95% CI: 1.0–1.4). Injuries due to falls and cognitive impairment mostly occurred after retirement age, and thus we did not observe major differences in outcomes related to receiving disability benefits or earnings. The cohort increased remaining life years by 1.3 months or 0.11 years (95% CI: 0.09–0.14) to 83.9 years on average, with a slightly smaller increase in QALYs (0.08, 95% CI: 0.06–0.11). Because elimination of sleep medications increased life expectancy, we observed an associated increase in medical spending ($1,260, 95% CI: 590–1930). When not accounting for this increased life expectancy, there was a reduction of $1880 (95% CI: 1500–2270) in medical costs on average (or 0.5% (95% CI: 0.4–0.6) of remaining lifetime medical costs). Controlling for insomnia status at baseline in the counterfactual scenario did not change these results significantly; eliminating future use of sleep medications reduced falls by 8.0% (95% CI: 6.3–9.8), reduced lifetime incidence of cognitive impairment by 2.4% (95% CI: 1.9–2.9), and increased remaining life years by 1.4 months or 0.12 years (95% CI: 0.10–0.15) (Supplementary data 1, Table S6).

**Table 2:** Comparison between lifetime outcomes in status quo and counterfactual scenario, in which future prescription sleep medications were eliminated.

	Status quo: using sleep medication at baseline	Counterfactual: no future sleep medication	Difference	Percentage difference
Sleep medication use (% at start)	100 (100–100)	100 (100–100)	0 (0–0)	(0–0)
Sleep medication use (avg lifetime %)	51.7 (50.8–52.7)	0 (0–0)	−51.7 (−52.7 to −50.8)	−100 (−100 to −100)
Injury due to fall (% ever)∗	60.8 (59.6–62.0)	55.6 (54.5–56.8)	−5.16 (−5.94 to −4.39)	−8.49 (−9.70 to −7.28)
TICS cognitive score <12 (% ever)∗	75.5 (74.8–76.3)	73.9 (73.1–74.8)	−1.62 (−1.94 to −1.29)	−2.14 (−2.58 to −1.70)
Living in nursing home (% ever)∗	24.6 (23.3–25.9)	24.1 (22.8–25.3)	−0.53 (−0.65 to −0.40)	−2.14 (−2.64 to −1.65)
Any limitations in ADLs (% ever)∗	81.4 (80.7–82.0)	81.3 (80.6–81.9)	−0.10 (−0.14 to −0.07)	−0.13 (−0.17 to −0.09)
Any limitations in IADLs (% ever)∗	79.1 (78.5–79.8)	78.8 (78.2–79.5)	−0.29 (−0.36 to −0.22)	−0.37 (−0.46 to −0.28)
Avg nr of survey waves with any injury due to fall∗	1.53 (1.45–1.60)	1.31 (1.25–1.38)	−0.21 (−0.25 to −0.18)	−13.85 (−15.87 to −11.83)
Avg years with TICS cognitive score <12∗	5.63 (5.51–5.76)	5.35 (5.20–5.50)	−0.28 (−0.33 to −0.23)	−5.00 (−5.92 to −4.09)
Avg years in nursing home∗	1.22 (1.13–1.32)	1.19 (1.09–1.28)	−0.04 (−0.04 to −0.03)	−2.98 (−3.62 to −2.33)
Avg years with limitations in any ADLs∗	5.94 (5.80–6.09)	5.91 (5.77–6.06)	−0.03 (−0.04 to −0.02)	−0.53 (−0.66 to −0.40)
Avg years with limitations in any IADLs∗	5.38 (5.26–5.51)	5.32 (5.20–5.44)	−0.06 (−0.07 to −0.05)	−1.16 (−1.38 to −0.95)
Avg years receiving disability benefits∗	1.27 (1.23–1.31)	1.27 (1.23–1.31)	−0.00 (−0.00 to −0.00)	−0.03 (−0.06 to −0.00)
Avg years working for pay∗	3.12 (3.04–3.19)	3.12 (3.04–3.20)	0.00 (0.00–0.00)	0.05 (0.03–0.07)
Avg earnings (x 1000, 2024$, disc.)∗	172 (165–179)	172 (165–179)	0.03 (0.00–0.06)	0.02 (0.00–0.04)
Avg earnings (x 1000, 2024$, disc.)	160 (153–166)	160 (154–166)	0.05 (0.01–0.10)	0.03 (0.01–0.06)
Avg medical costs (x 1000, 2024$, disc.)∗	360 (349–371)	358 (348–369)	−1.88 (−2.27 to −1.50)	−0.52 (−0.62 to −0.42)
Avg medical costs (x 1000, 2024$, disc.)	367 (357–378)	369 (358–379)	1.26 (0.59–1.93)	0.34 (0.17–0.52)
Avg age at death (years)	83.7 (83.6–83.9)	83.9 (83.7–84.0)	0.11 (0.09–0.14)	0.14 (0.10–0.17)
Avg life years	18.1 (17.9–18.2)	18.2 (18.0–18.4)	0.11 (0.09–0.14)	0.63 (0.48–0.77)
Avg QALYs	10.5 (10.2–10.9)	10.6 (10.2–11.0)	0.08 (0.06–0.11)	0.80 (0.57–1.03)
Avg QALYs (x 1000, 2024$, disc.)	1193 (1153–1232)	1201 (1162–1239)	7.8 (5.6–10.0)	0.65 (0.46–0.85)
Cohort total life years (x 1000)	276,492 (274,103–278,882)	278,223 (275,820–280,627)	1731 (1332–2130)	0.63 (0.48–0.77)
Cohort total QALYs (x 1000)	161,300 (155,327–167,272)	162,586 (156,702–168,470)	1286 (928–1645)	0.80 (0.57–1.03)
Cohort total nursing home years∗ (x 1000)	17,233 (15,883–18,582)	16,720 (15,401–18,040)	−512 (−629 to −395)	−3.0 (−3.6 to −2.3)
Cohort total years claiming disability∗ (x 1000)	17,851 (17,285–18,416)	17,845 (17,279–18,412)	−5.0 (−9.8 to −0.3)	−0.03 (−0.06 to −0.00)
Cohort total years working for pay∗ (x 1000)	43,932 (42,844–45,020)	43,953 (42,864–45,042)	20.9 (11.1–30.8)	0.05 (0.03–0.07)
Cohort total earnings (millions, 2024$, disc.)∗	2,427,346 (2,329,718–2,524,975)	2,427,802 (2,330,109–2,525,494)	456 (9–902)	0.02 (0.00–0.04)
Cohort total earnings (millions, 2024$, disc.)	2,445,120 (2,346,751–2,543,489)	2,445,913 (2,347,585–2,544,241)	793 (114–1473)	0.03 (0.01–0.06)
Cohort total medical costs (millions, 2024$, disc.)∗	5,074,540 (4,923,844–5,225,235)	5,047,994 (4,898,978–5,197,011)	−26,545 (−31,995 to −21,096)	−0.52 (−0.62 to −0.42)
Cohort total medical costs (millions, 2024$, disc.)	5,617,940 (5,454,702–5,781,178)	5,637,210 (5,472,209–5,802,211)	19,270 (8970–29,570)	0.34 (0.17–0.52)
Cohort total QALYs (millions, 2024$, disc.)	18,241,036 (17,639,301–18,842,771)	18,360,106 (17,767,185–18,953,027)	119,070 (85,156–152,983)	0.65 (0.46–0.85)

The cohort sample consisted of HRS respondents who indicated regularly using prescription medication to help sleep. All outcomes indicated with an asterisk (∗) were based on simulation timepoints (waves) that were in common between the status quo and counterfactual scenarios (i.e. not accounting for extended lifespan). The remainder of the outcomes included all simulated time points. Results expressed as a reduction in years are not conditional on respective outcomes. Outcomes in the (Percentage) Difference columns are statistically significant when the confidence intervals do not include 0.

ADLs: activities of daily living; IADLs: instrumental activities of daily living; JSS: QALYs: quality-adjusted life years; TICS: telephone interview for cognitive status. All dollar values are in 2024$ and discounted 3% for years after the start of the simulation. QALYs are valued at $150,000 (in 2024$).

Per capita lifetime sleep medication burden among our cohort of people with prevalent sleep medication use over age 50 was $6.6 K (95% CI: 4.7–8.5), due to $7.8 K in lost QALYs (95% CI: 5.6–10.0) and $0.05 K in lost earnings (95% CI: 0.01–0.1), offset by −$1.26 K in medical expenditures (95% CI: −1.93 K to −0.59 K). The aggregate burden of sleep medication for this cohort in the US was $101 billion (95% CI: 72–129) due to $119 billion in lost QALYs (95% CI: 85–153) and $0.8 billion in lost earnings (95% CI: 0.1–1.5), offset by −$19 billion in medical expenditures (95% CI: −30 to −9). Approximately 1.7 million life years (95% CI: 1.3–2.1), and 1.3 million QALYs (95% CI: 0.9–1.6), were lost due to prescription sleep medication use.

Lifetime differences in outcomes were not distributed equally by age. The largest relative differences between status quo and counterfactual scenarios were observed for people between ages 70 and 74 at the beginning of the simulation (Supplementary data 1, Figure S4). For example, relative gains in duration with cognitive impairment or functional limitations were largest for this age group, as was relative reduction in duration of nursing home living. These relative differences are a product of remaining lifespan, probabilities and time to events. (Supplementary data 1, Table S7a–h for absolute differences by age). Lifetime differences were also not distributed equally by sex, educational attainment, and race-ethnicity (Supplementary data 1, Tables S7i–p). Prevalence of sleep medication use in the starting cohort differed by sex, educational attainment and age, but not by race-ethnicity for this age group (Supplementary data 1, Table S8).

**Fig. 2:**
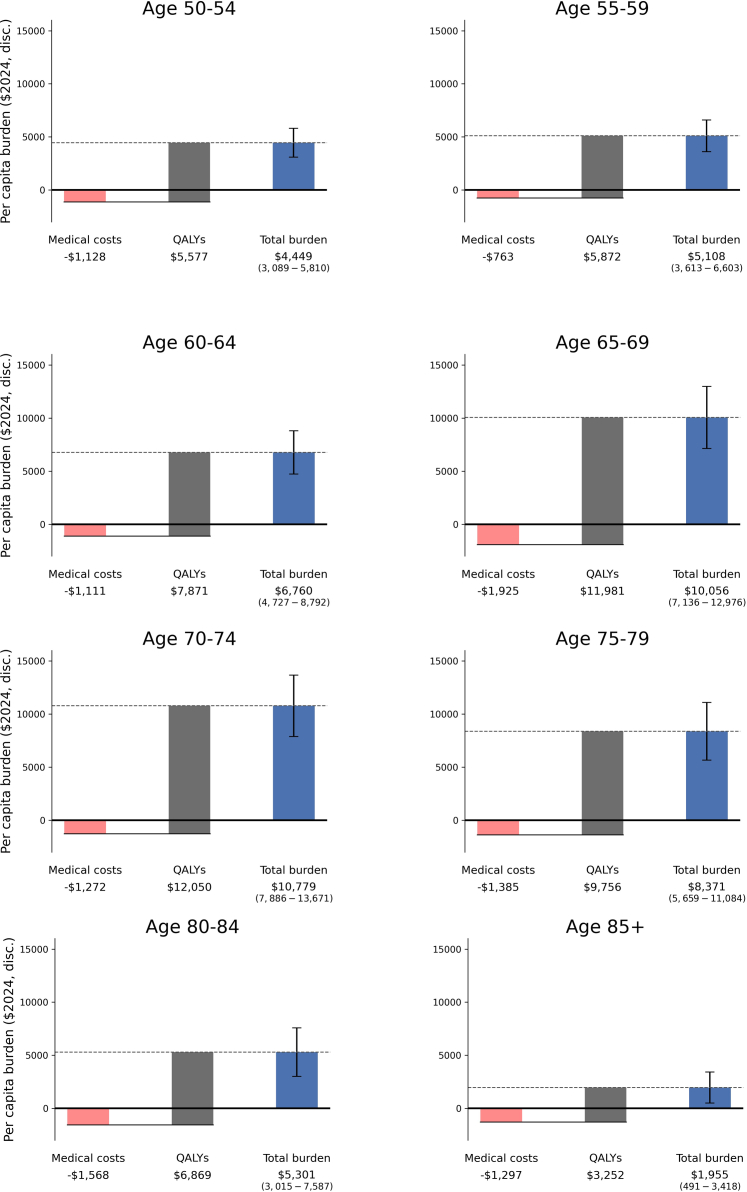
Per capita burden of prescription sleep medication for people over age 50, by age at the start of the simulation. All values are in 2024$ and discounted 3% for years after the start of the simulation. Lost earnings were not shown in the chart because of small amounts ($0–$120, refer to Supplementary data 1 Tables S7a–h) relative to medical costs and value of QALYs. QALYs are valued at $150,000 (in 2024$). Error bars indicate 95% confidence intervals.

**Fig. 3:**
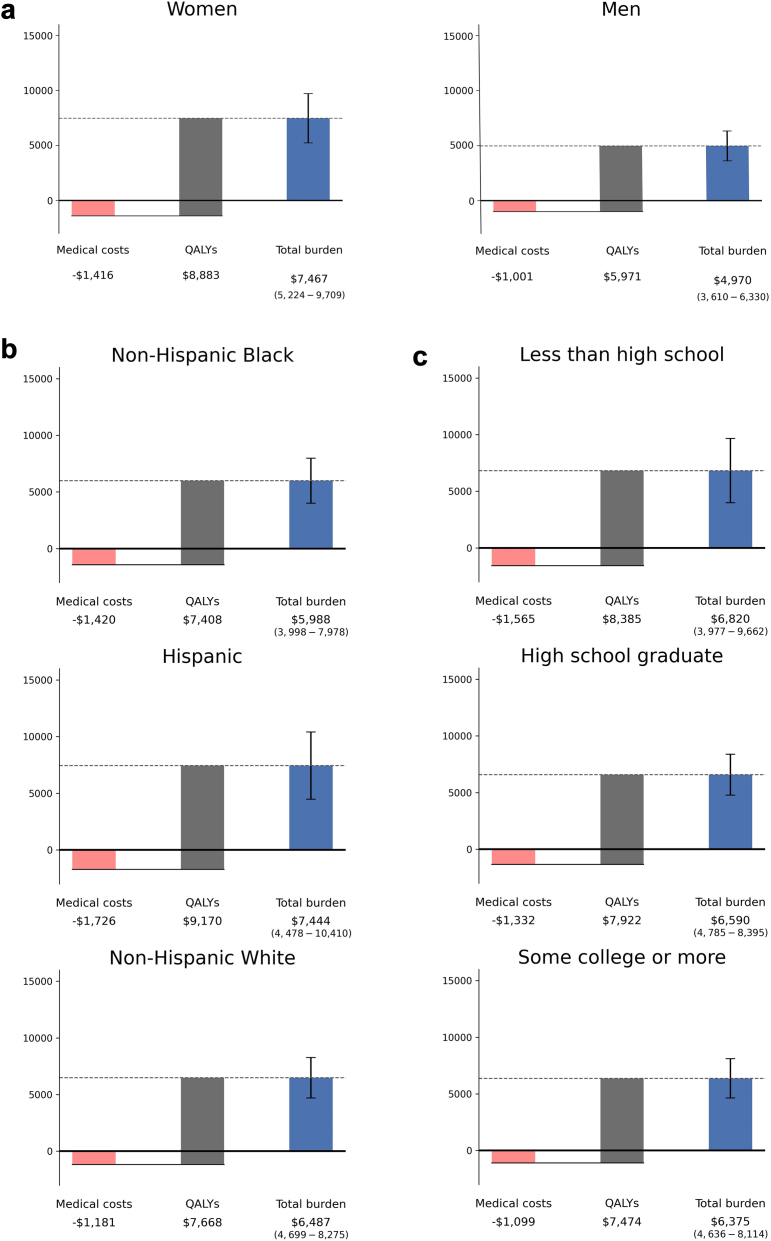
Per capita burden of prescription sleep medication for people over age 50, by a) sex, b) race-ethnicity, or c) educational attainment. All values are in 2024$ and discounted 3% for years after the start of the simulation. Lost earnings were not shown in the chart because of small amounts ($10–$60, refer to Supplementary data 1 Tables S7i–p) relative to medical costs and value of QALYs. QALYs are valued at $150,000 (in 2024$). Error bars indicate 95% confidence intervals.

**Fig. 4:**
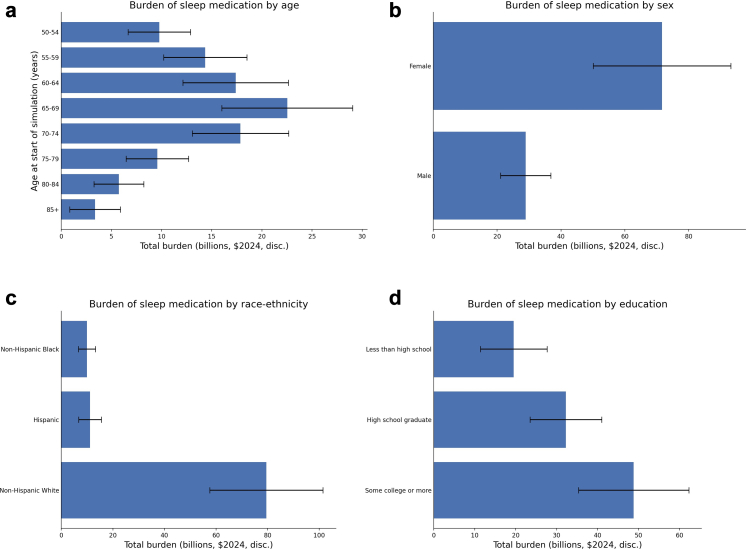
Aggregate burden of prescription sleep medication for people over age 50 in the US, by a) age at start of the simulation, b) sex, c) race-ethnicity, or d) educational attainment. Total burden includes lost QALYs valued at $150,000, lost earnings, and medical costs. All values are in 2024$ and discounted 3% for years after the start of the simulation. Error bars indicate 95% confidence intervals.

Per capita, the lifetime burden of sleep medication use was approximately $10 K (2024$, 3% discounted) for ages 65 to 74 at baseline (Fig. 2). The elimination of prescription sleep medication for this group creates value by preventing falls and cognitive impairment in the near-term while a reasonably long remaining lifespan allows for benefits to accrue. The average lifetime burden did not differ significantly by sex, race-ethnicity, or educational attainment (Fig. 3). Fig. 4 illustrates the differences on an aggregate level (accounting for the number of people who use sleep medication in each group), with the largest share of the total cohort burden for people ages 65–69 ($23 billion (95% CI: 16–29)), women (($72 billion (95% CI: 50–93)), non-Hispanic white people ($80 billion (95% CI: 58–101)), and those with at least some college education ($49 billion (95% CI: 35–62)).

## Discussion

In this study, we employed a dynamic microsimulation model (FEM) to estimate the lifetime burden of prescription sleep medication use for individuals over age 50 in the US. From a clinical standpoint, the results indicate that long-term treatment is less than beneficial: if a patient tries to improve quality of life by regularly using sleep drugs to improve sleep, then, on the whole, this aim is achieved less effectively than if they had never used them. Prescription sleep medications had small but pervasive and persistent effects on quality of life that added up to produce a considerable economic burden. The lifetime burden of prescription sleep medication use for an individual was valued at $6.6 K per person and $101 billion in this US cohort over age 50. The majority of lifetime burden is attributed to decreased quality of life. Eliminating sleep medication use, modeled gains in life expectancy were accompanied by gains in quality-adjusted life years, suggesting that most of these extra months would be in good health, on average. This was true even when we accounted for insomnia status at baseline in our sensitivity analysis. Although individual effects appear modest, they represent gains in overall health and quality of life that are achievable by reducing the frequency or duration that these medications are prescribed. The overall burden, and subsequently largest potential improvement in the near-term, was particularly large among 65-74-year-olds, suggesting this group would be important to target to reduce burden. Aggregate results from subgroup analysis of other demographic characteristics (sex, race-ethnicity, and educational attainment) mostly reflect differences in average life expectancy, and prevalence of sleep medication use.

Our study has several limitations. Prior studies have found that initial benefits of sleep medication (i.e. reduced insomnia), are modest and likely not sustained with chronic use.^38^^,^^39^ Although we did not incorporate rebound insomnia in the microsimulation approach, studies show that a small percentage of people who stop taking their prescription sleep medications will experience a short-term rebound effect with increased insomnia symptoms, and then revert to levels of insomnia as when on the medication.^40^^,^^41^ At most, we expect rebound effects that last for four weeks, which would result in a reduction of 0.007 QALYs, since estimated QALYs (for a full year) are reduced by 0.09 for people with insomnia compared to those without.^42^ This translates to an attenuation of 8% in our estimated QALY gains for the subset of individuals with insomnia rebound effects (or a reduction of approximately $1 K in the average burden of prescription sleep medication). In sensitivity analyses that allowed persistent insomnia to impact outcomes, we did not find substantive differences in outcomes. Additionally, for people who use sleep medication long-term, dependence formation may shape their perception about the impact and effectiveness of the therapy, leading some individuals to overlook side effects or place greater emphasis on perceived improvements. Our baseline model accounts for this possibility by allowing for heterogeneous (positive, negative, neutral) effects at the individual level. Another limitation is that we do not include obstructive sleep apnea (OSA), a contributor to poor sleep, in our model. This is for two key reasons: (1) HRS data limitations and availability; and (2) OSA is commonly un (der)diagnosed, so we are likely to miss cases. Another limitation of our model is that it is adjusted for measured variables, but unobserved variables may explain, in part, the effects we observe. For example, some respondents have comorbid psychiatric or other medical conditions that may impact how discontinuation of these medications affect their overall wellbeing, but we do not directly control for these conditions. Disentangling the impacts on persons with complex comorbid conditions is an important nuance to consider in future studies. Finally, validation exercises showed that the simulation performed well for years with available data, but there is an inherent uncertainty of predicting future trends.

Despite the common use of sleep medications, CBT-I is the recommended first-line insomnia treatment. CBT-I includes behavioral modification, cognitive restructuring, and relaxation training.^2^^,^^7^^,^^9^ It can be delivered in-person or via telemedicine, telephone, app, or website. CBT-I is as effective as pharmacological treatment in the short-run and more effective in the long-run, without the adverse side effects associated with sleep medications.^10^^,^^11^^,^^43^^,^^44^ While some patients may experience daytime sleepiness and downstream consequences during the initial period of CBT-I, the side effect is generally transient on the path to better sleep.^45^ Thus, patients may experience additional benefits if they were to receive CBT-I instead of a (continuing or new) sleep medication prescription. Many patients with insomnia may lack access to in-person CBT-I due to location, provider shortages, insurance coverage, or simply lack of awareness. To improve access to CBT-I, apps have been developed that are free-of-charge (e.g. Veterans Administration's CBT-I Coach) and have been shown to be effective in reducing insomnia.^46^ Improving clinician and patient knowledge of these apps would likely increase uptake of CBT-I.

Our results support the need to prescribe sleep medications less often. Use of behavioral economics interventions to limit inappropriate prescribing has demonstrated success in studies across several similar domains and medications, including opioids, and antibiotics, and may offer a pathway toward reduction of sleep medication use.^47^, ^48^, ^49^, ^50^, ^51^, ^52^ Our analysis suggests that reductions in the use of prescription sleep medications would have positive downstream impacts on individuals and the population.

## Contributors

Conceptualization, Methodology, Writing—review & editing: JNC, JAL, SDP, MDS, JND.

Conceptualization, Data curation, Formal analysis, Investigation, Methodology, Project administration, Software, Supervision, Validation, Writing—original draft, Writing—review & editing: HHJ, JT.

Investigation, Methodology, Supervision, Writing—review & editing: BT.

Conceptualization, Funding acquisition, Methodology, Project administration, Supervision, Writing—review & editing: JND.

## Data sharing statement

Survey data used in the microsimulation are available directly from the Health and Retirement Study, the Medical Expenditure Panel Survey, and the Medicare Current Beneficiary Survey. Microsimulation and analysis code can be obtained from the authors upon request.

## Declaration of interests

MS is a board member of Physicians for Responsible Opioid Prescribing and discloses board participation in the NIAMS SKOAP Trial. BT discloses participation in the International Microsimulation Association. JND discloses participation at the NHLBI Cascade Screening DSMB. All other authors have no declarations to disclose.
