# Supplementary material for: Lifetime burden of prescription medication for insomnia in middle-aged and older adults in the US: a microsimulation study
Source: Lancet Reg Health Am. 2025 Oct 24;52:101284. doi: 10.1016/j.lana.2025.101284 (PMC12593590; doi:10.1016/j.lana.2025.101284)
Supplement: Supplementary data 1 [file mmc1.pdf]

Lifetime burden of prescription medication for insomnia in middle-aged and older adults in the US: a microsimulation study

Hanke Heun-Johnson, PhD; Johanna Thunell, PhD; Jonathan N. Cloughesy; Jeffrey A. Linder, MD, MPH, FACP; Stephen D. Persell, MD MPH; Mark D. Sullivan, MD PhD; Bryan Tysinger, PhD; Jason N. Doctor, PhD

October 2025

TABLE OF CONTENTS

Section 1. Validation of simulation variables

Section 2. Analysis of z-drug subgroup

Section 3. Insomnia

Section 4. Functional limitations

Section 5. Imputation of injury due to a fall

Section 6. Supplementary results

List of Supplementary Tables and Figures

Figure S1 Two-year ROC curves of simulation variables 3

Figure S2 Ten-year ROC curves of simulation variables 4

Table S1 Prescription sleep medications in MEPS z-drug imputation model....5

Table S2 Demographic and health characteristics of imputed z-drug simulation cohort....6

Table S3 Survey questions for limitations in physical functioning....9

Table S4 Prevalence of PFLs and (I)ADLs....10

Figure S3 Imputation alignment of injury due to a fall for ages 51-64....11

Figure S4 Percentage change in lifetime outcomes after sleep medication elimination by age....12

Table S5 Lifetime effects of sleep medication for imputed z-drug cohort....13

Table S6 Lifetime effects of sleep medication while controlling for insomnia 14

Table S7a-h Lifetime effects of sleep medication elimination, by age.... 15-22

Table S7i-j Lifetime effects of sleep medication elimination, by sex.... 23-24

Table S7k-m Lifetime effects of sleep medication elimination, by race-ethnicity 25-27

Table S7n-p Lifetime effects of sleep medication elimination, by educational attainment.... 28-30

Table S8 Use of sleep medication by age, sex, race-ethnicity, and educational attainment....31

Section 1. Validation of simulation variables

To validate new and existing variables in the expanded FEM simulation framework, we assessed internal validity using the 2008 cohort of HRS respondents. We simulated their outcomes and compared to 2010 (two-year) and 2018 (ten-year) intervals.

Outcomes were simulated using a five-fold cross validation approach: HRS survey respondents in years 2008 through 2018 were randomly split into five equal parts; four parts were used to generate transition models for the simulation (years 2010-2018), and one part was used as the starting cohort for the simulation in 2008. This was repeated five times so that all 2008 respondents entered the simulation once. Respondents were included in any of the outcomes for which they had non-missing data in the simulation start year (2008) and the evaluation year (either 2010 or 2018). If a respondent had died before the end of the evaluation or had missing data in 2018, the last known value was used. For absorbing variables (cancer, diabetes, heart disease, high blood pressure, lung disease, stroke, and death), only those without the condition in 2008 were included in the analysis. The simulation was repeated 1000 times and the prediction from these simulations averaged for each respondent. We then constructed the receiver operating characteristic (ROC) curves using these averaged probability scores by varying the classification threshold from 0 to 1. For each threshold, we compared the predicted classification to the actual survey outcome to calculate the sensitivity (true positive rate) and specificity (1 - false positive rate), and plotted these values to generate the ROC curves. The area under the ROC curve (AUROC) was calculated to quantify predictive performance.

Simulation predictions are acceptable to good on a two-year horizon for new variables: injuries due to a fall (AUROC of 0.72), using prescription sleep medications (0.80), and any limitations in physical functioning (0.89) (**Figure S1**). The AUROC values are slightly lower, but still acceptable to good, for outcomes on a ten-year horizon, at 0.67, 0.71, and 0.83, respectively (**Figure S2**).

Existing simulation variables are also listed in **Figures S1** and **S2**, with AUROC values comparable to our standard microsimulation framework. Some of these existing variables are directly impacted by the newly created variables, such as ADLs, IADLs, living in a nursing home, cognitive impairment (classified as a TICS score < 12), and mortality; some are only indirectly impacted, such as BMI status and health conditions.

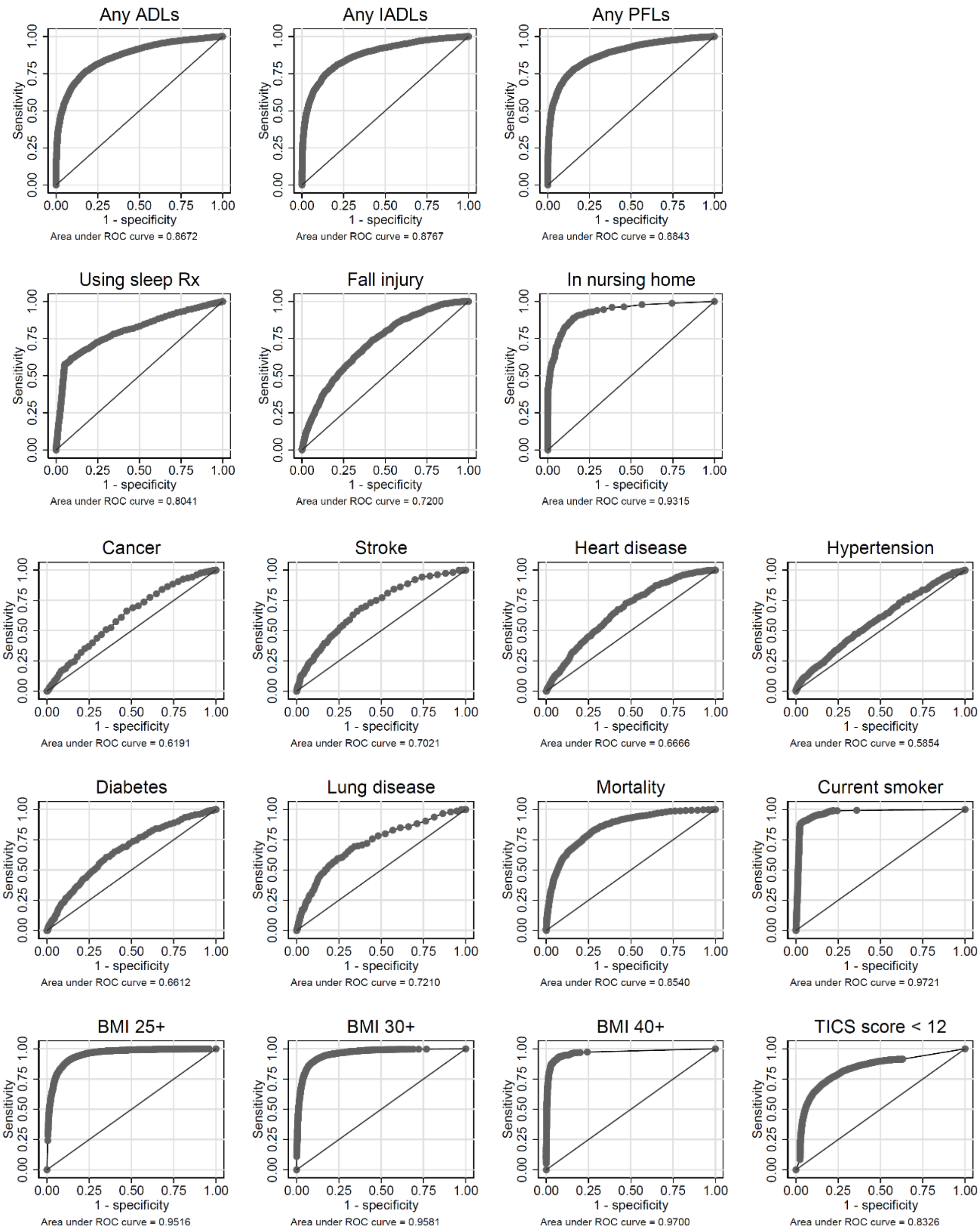

Figure S1: Two-year ROC curves and AUROC for new and existing variables in FEM simulation framework for 2008 cohort, using transition models based on 2010-2018 HRS survey data, and observed outcomes in 2010. ADLs: functional limitations in Activities of Daily Living; BMI: body mass index; IADLs: functional limitations in Instrumental Activities of Daily Living; PFLs: Limitations in Physical Functioning; sleep Rx: prescription sleep medication use; TICS score < 12: cognitive impairment.

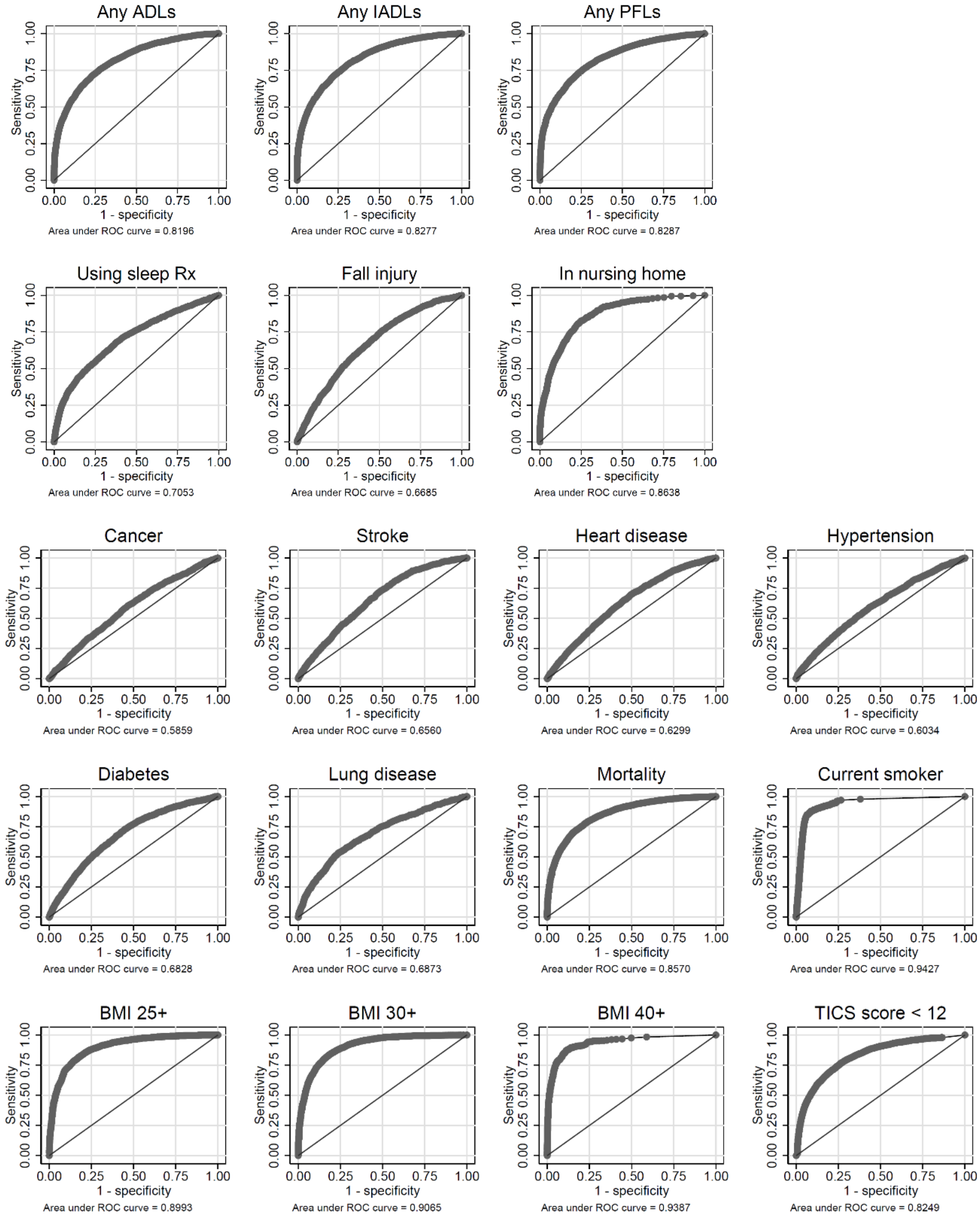

Figure S2: Ten-year ROC curves and AUROC for new and existing variables in FEM simulation framework for 2008 cohort, using transition models based on 2010-2018 HRS survey data, and observed outcomes in 2018. ADLs: functional limitations in Activities of Daily Living; BMI: body mass index; IADLs: functional limitations in Instrumental Activities of Daily Living; PFLs: Limitations in Physical Functioning; sleep Rx: prescription sleep medication use; TICS score < 12: cognitive impairment.

Section 2. Analysis of z-drug subgroup

To establish a simulation cohort of people who specifically use z-drugs among those who report regularly using prescription sleep medication to help sleep in HRS data, we imputed z-drug use with a model based on 2013-2018 data from the Medical Expenditure Panel Survey (MEPS). We used MEPS variables AGE, RACE, HISPANX, SEX, EDU, and BMI; prescription drug use was determined based on the variable RXNAME, with search terms for z-drugs and their substitutes, tricyclic antidepressants, and benzodiazepines as listed in **Table S1**.

Table S1: Prescription medication drug names to categorize z-drugs, tricyclic antidepressants, and benzodiazepines, as value for RXNAME variable in MEPS data.

**Z-drugs**	Ambien, Edluar, Eszopiclone, Intermezzo, Lunesta, Zaleplon, Zolpidem, Zolpimist
**Tricyclic Antidepressants**	Desipramine, Doxepin, Imipramine, Mirtazapine, Norpramin, Nortriptyline, Pamelor, Protriptyline, Tofranil, Trazodone, Trimipramine
**Benzodiazepines**	Alprazolam, Ativan, Chlordiazepoxide, Clonazepam, Diazepam, Doral, Estazolam, Flurazepam, Halcion, Klonopin, Lorazepam, Loreev, Oxazepam, Quazepam, Quazepam, Restoril, Temazepam, Triazolam, Triazolam

The MEPS sample used to create a probit imputation model was restricted to ages 50 and older, with predictors (listed above) common to both HRS and MEPS. Final model specification and coefficients can be found in **Supplementary data 2**. With this model, we imputed the probability of using z-drugs among HRS respondents who reported regularly taking prescription sleep medication to help sleep. **Table S2** contains the demographic characteristics and health status of this imputed assignment of 2016 HRS respondents. This table shows the average for 75 Monte Carlo repetitions, in which we created randomly-drawn probability cutoffs that were compared to the imputed probability of z-drug use, varying for each Monte Carlo repetition. These 75 cohorts form the starting cohorts of the simulation.

Approximately 23% of HRS respondents who regularly take prescription sleep medication to help sleep was predicted to use z-drugs, using the MEPS imputation model. This translates to 3.1% (95% CI: 2.8-3.3%) of the total HRS cohort over age 50. Compared to those taking other sleep medications, individuals who use z-drugs were slightly younger (64.0 $\text{yrs}_{\text{z-drug}}$ vs 66.2 $\text{yrs}_{\text{other drug}}$), and higher educated ($57.4\%_{\text{z-drug}}$ had some college education or more vs $47.3\%_{\text{other drug}}$). The two groups had similar prevalence of health conditions, functional limitations, and nursing home residence, and similar average yearly medical costs (**Table S2**). The lifetime effects of sleep medication elimination for this specific cohort are shown in **Table S5**.

Table S2: Demographic and health characteristics of imputed simulation cohort (in “Z-drugs” column). This cohort included anyone over age 50 who was predicted to take z-drugs at simulation baseline, and is a subset of HRS

respondents who report regularly taking prescription sleep medications to help sleep. Within this sleep medication group, z-drug use was imputed using a model from the Medical Expenditure Panel Survey. The values in parentheses indicate the 95% confidence intervals based on 75 repetitions in the simulation. ADLs: activities of daily living; BMI: body mass index; IADLs instrumental activities of daily living; PFLs: physical functioning limitations; TICS: Telephone Interview for Cognitive Status.

	**Z-drugs**	**Other prescription sleep medications**
Sample size, N	622 (583-661)	2,106 (2,067-2,145)
Sample size weighted, N	3,483,523 (3,150,519-3,816,527)	11,808,731 (11,475,727-12,141,734)
Age*, mean	64.0 (63.1-64.9)	66.2 (65.9-66.4)
Female*, %	60.7 (56.4-65.0)	62.8 (61.5-64.1)
Male*, %	39.3 (35.0-43.6)	37.2 (35.9-38.5)
Black*, %	12.7 (10.7-14.6)	10.2 (9.7-10.8)
Hispanic*, %	9.3 (7.0-11.6)	9.9 (9.2-10.6)
White*, %	78.1 (75.2-80.9)	79.9 (79.0-80.7)
Educational attainment*, %		
Less than high school	16.0 (12.3-19.6)	19.5 (18.4-20.6)
High school graduate	26.6 (22.9-30.3)	33.2 (32.1-34.3)
Some college or more	57.4 (52.4-62.5)	47.3 (45.8-48.8)
BMI*, kg/m^2	29.2 (28.5-29.9)	28.8 (28.6-29.0)
Health conditions (ever), %		
High blood pressure	68.5 (63.9-73.0)	68.9 (67.5-70.2)
Diabetes	31.4 (27.5-35.2)	30.7 (29.6-31.9)
Heart disease	34.0 (29.7-38.2)	34.8 (33.6-36.1)
Myocardial infarction	15.2 (11.5-18.8)	15.7 (14.6-16.8)
Lung disease	20.5 (16.6-24.5)	21.0 (19.8-22.1)
Stroke	14.9 (11.6-18.3)	15.8 (14.8-16.7)
Injured seriously enough from fall to need medical treatment (in last 2 yrs), %	14.7 (10.8-18.6)	16.2 (14.2-18.1)
TICS cognitive score < 12, %	27.0 (23.3-30.7)	31.6 (30.5-32.7)
Any ADLs, %	35.4 (31.1-39.7)	38.7 (37.5-40.0)
Any IADLs, %	33.3 (29.2-37.4)	36.4 (35.2-37.6)
Any PFLs, %	67.6 (63.4-71.9)	70.8 (69.6-72.1)
Living in nursing home, %	4.4 (2.8-6.0)	6.2 (5.7-6.7)
Average medical costs ($2024)	25,603 (23,875-27,332)	27,668 (27,148-28,188)

* Covariates in z-drug imputation model among those taking prescription sleep medication

Section 3. Insomnia

As a sensitivity analysis, we included a measure of insomnia in transition models that assess the probability of fall injuries and cognitive state associated with sleep medications. Insomnia was included as a main effect and as an interaction with sleep medication use (see **Supplementary data 2** for model coefficients).

We quantified insomnia in the HRS with a modified version of the Jenkins Sleep Scale (JSS), based on four questions on a 0-8 point scale, on trouble falling asleep, waking up during the night, waking up too early, or feeling rested in the morning. The answers count as 0 points for “rarely/never”, 1 point for “sometimes”, and 2 points for “most of the time”, with points reversed for the question on whether a respondent feels rested. The unmodified JSS commonly quantifies severity based on the number of days, in which answers range from 0 (“not at all”) to 5 points (“22-31 days”), with a maximum total of 20 points for four questions. This answering scale is not available in the HRS. Analogous to studies by Chen et al. (2024) and Tibubos et al. (2020), we based a cutoff of insomnia on a percentile rank in the population. In the 2016 HRS population over age 50, 50.8% scored 0-2 points, and the 51st percentile is at a score of 3 points; thus, we utilized a score of 3 or more points (out of 8) as a dichotomous indicator of insomnia. In our starting cohort of people who indicated regular sleep medication use, 75.8% scored positively on this scale (see **Table S6**).

To assess the effect of sleep medication elimination in the simulation while accounting for insomnia, we kept the indicator of insomnia in the fall injury and cognition models unchanged in the counterfactual scenario. The outcomes of the simulation are presented in **Table S6**.

Chen, M. Y., Bai, W., Wu, X. D., Sha, S., Su, Z., Cheung, T., Pang, Y., Ng, C. H., Zhang, Q., & Xiang, Y. T. (2024). The network structures of depressive and insomnia symptoms among cancer patients using propensity score matching: Findings from the Health and Retirement Study (HRS). *J Affect Disord*, *356*, 450-458. https://doi.org/10.1016/j.jad.2024.04.035

Tibubos, A. N., Zenger, M., Schmalbach, B., Beutel, M. E., & Brahler, E. (2020). Measurement invariance, validation and normative data of the Jenkins Sleep Scale-4 (JSS-4) in the German general population across the life span. *J Psychosom Res*, *130*, 109933. https://doi.org/10.1016/j.jpsychores.2020.109933

Section 4. Functional limitations

In this study, ADLs are quantified by counting affirmative answers to the questions "Because of a health or memory problem do you have any difficulty with: 1) bathing or showering; 2) dressing, including putting on shoes and socks; 3) eating, such as cutting up your food; 4) getting in or out of bed; 5) walking across a room; and 6) using the toilet, including getting up and down?". Similarly, the number of IADLs were determined by counting affirmative answers to questions "Because of a health or memory problem, do you have any difficulty: 1) making phone calls; 2) shopping for groceries; 3) preparing a hot meal; and 4) managing your money -- such as paying your bills and keeping track of expenses?". We did not include other IADLs (e.g. managing medications, or housekeeping) because the medical cost models required that variables were available in both the HRS and MCBS.

Since injuries from falls might have subtler effects on physical functioning than captured by ADLs and IADLs, we created an additional measure to identify more common mobility issues that could impact quality of life and affect medical costs: limitations in physical functioning (PFLs). For this we used measures that were similar in HRS and MEPS: problems with walking several blocks, climbing one flight of stairs, reaching overhead, lifting a heavy object, or grasping a small item. The PFL variable was implemented as categorical with 0, 1, or 2 or more limitations in physical functioning (indicated as "any difficulty"), aiming for equal distributions of these categories among the relevant HRS and MEPS subsamples (ages 51-64 without Medicare coverage). The goal of having equal distributions is important for the medical cost models.

Medical costs in the simulation were modeled with external data: MEPS for simulants ages 51-64 without Medicare coverage, and MCBS for those covered by Medicare or over age 65. Prior to this study, our FEM medical cost model for simulants ages 51-64 without Medicare coverage (based on MEPS data) did not account for functional limitations, as no corresponding (I)ADL measures were available in MEPS, and the prevalence of limitations in (I)ADLs were generally too low in this age group.

The PFL (limitations in physical functioning) measure filled this gap by using measures that existed in both MEPS and HRS datasets, and were more prevalent in relevant age groups. The variables are listed in **Table S3** below.

Table S3: Survey questions for limitations in physical functioning in HRS and MEPS that were used to create the PFL count variable. A dummy variable for two or more PFLs was included in the medical cost model for those under 65 without Medicare coverage. HRS, Health and Retirement Study; MEPS, Medical Expenditure Panel Survey; PFL, limitations in physical functioning. *In several survey years, the option “Completely unable to walk” was included as an answer to certain questions related to walking (e.g. walking several blocks, or climbing 10 steps). In these cases, the other walking-related questions were automatically set to “completely unable to do it” in the survey.

<table>
<tr><th></th><th colspan="2">HRS (RAND Fat files)</th><th colspan="2">MEPS</th></tr>
<tr><td rowspan="3">Filter question</td><td colspan="2" rowspan="3"></td><td colspan="2">[WLKLIM] Do you have difficulties walking, climbing stairs, grasping objects, reaching overhead, lifting, bending or stooping, or standing for long periods of time (because of an impairment or a physical or mental health problem)?</td></tr>
<tr><td>1 Yes</td><td>Count PFLs listed below</td></tr>
<tr><td>2 No</td><td>No PFLs</td></tr>
<tr><td>Walking several blocks</td><td colspan="2">[G001] Because of a health problem do you have any difficulty with walking several blocks?</td><td colspan="2">[WLKDIF] How much difficulty do you have walking about 3 city blocks or about a quarter of a mile?</td></tr>
<tr><td>Climbing one flight of stairs</td><td colspan="2">[G007] Because of a health problem do you have any difficulty with climbing one flight of stairs without resting?</td><td colspan="2">[STPDIF] How much difficulty do you have walking up 10 steps without resting?</td></tr>
<tr><td>Reaching overhead</td><td colspan="2">[G009] Because of a health problem do you have any difficulty with reaching or extending your arms above shoulder level?</td><td colspan="2">[RCHDIF] How much difficulty do you have reaching overhead, for example to remove something from a shelf?</td></tr>
<tr><td>Lifting a heavy object</td><td colspan="2">[G011] Because of a health problem do you have any difficulty with lifting or carrying weights over 10 pounds, like a heavy bag of groceries?</td><td colspan="2">[LFTDIF] How much difficulty do you have lifting something as heavy as 10 pounds, such as a full bag of groceries?</td></tr>
<tr><td>Grasping a small item</td><td colspan="2">[G012] Because of a health problem do you have any difficulty with picking up a dime from a table?</td><td colspan="2">[FNGRDF] How much difficulty do you have using fingers to grasp or handle something such as picking up a glass from a table or using a pencil to write?</td></tr>
<tr><td rowspan="2">Answers:</td><td>No</td><td>0</td><td>no difficulty</td><td>0</td></tr>
<tr><td>Yes
Can’t do
Don’t do</td><td>1 PFL count for each question</td><td>Some difficulty
A lot of difficulty
Completely unable to do it
[Completely unable to walk]*</td><td>1 PFL count for each question</td></tr>
</table>

To decide how to include PFL counts in the MEPS medical cost model, we compared counts (e.g. any, 0, 1 or 2+) between MEPS and HRS respondents. Although the prevalence of specific PFLs was only slightly different between the surveys, MEPS analyses showed a lower prevalence for any PFLs or having 1 PFL compared to the HRS (**Table S4**). This discrepancy may be attributed to the generalized filter question used in MEPS (**Table S3**), which may have increased the likelihood that respondents with one specific limitation inadvertently skipped the relevant follow-up questions that they would have otherwise endorsed. Despite this discrepancy, the 2+ PFL dummy variable added value by indicating functional limitations under age 65, with a prevalence of 15%, compared to single digit prevalence of (I)ADLs. The newly constructed 2+ PFL dummy is the most significant predictor of medical costs in this younger age group (**Supplementary data 2**). In the simulation, changes in PFL counts over time were modeled with an ordered probit of 0, 1 or 2+ PFLs, with simulants being able to transition between PFL count categories without restrictions (**Supplementary data 2**). Their status in each wave was subsequently used in the medical cost model.

Table S4: Weighted prevalence of physical functional limitations, and limitations in (instrumental) activities of daily living in HRS, MEPS, and MCBS among respondents over 50 in 2010. Absence of a value in a cell means that a measure is not available in that survey. The shaded cells indicate the variables used in medical cost models for different age groups. Means are survey-weighted, and 95% intervals are based on the standard error of the mean.

	MEPS	HRS		MCBS
	**Ages 51-64, Non-Medicare, non-institutionalized**	**Ages 51-64, Non-Medicare, not in nursing home**	**Medicare, or age 65+, not in nursing home**	**Medicare, or age 65+, not in nursing home**
Age (yrs)	57.0 (56.9-57.2)	57.0 (56.8-57.2)	73.3 (73.0-73.7)	73.0 (72.7-73.2)
Any PFLs (%)	16.4 (15.0-17.9)	27.3 (25.8-28.9)		
1 PFL (%)	3.1 (2.4-3.7)	12.6 (11.6-13.5)		
2+ PFLs (%)	13.4 (12.1-14.7)	14.8 (13.6-16.0)		
Any difficulty (%):				
Walking 3 blocks	13.6 (12.2-14.9)	16.4 (15.3-17.5)		
Climbing flight of stairs	11.3 (10.2-12.4)	9.5 (8.5-10.5)		
Reaching overhead	9.3 (8.3-10.4)	11.3 (10.2-12.4)		
Lifting 10 pounds	9.8 (8.7-10.8)	14.2 (13.2-15.3)		
Grasping small item	6.1 (5.3-6.9)	3.9 (3.4-4.4)		
Any ADLs (%)		10.1 (9.1-11.1)	23.3 (22.1-24.5)	28.9 (27.3-30.6)
1 ADL (%)		4.8 (4.2-5.3)	10.5 (9.9-11.1)	13.9 (12.9-14.9)
2 ADLs (%)		2.6 (2.1-3.1)	4.9 (4.4-5.5)	6.4 (5.8-7.1)
3+ ADLs (%)		2.7 (2.2-3.2)	7.9 (7.1-8.7)	8.6 (7.6-9.5)
Any IADLs (%)		7.6 (6.8-8.4)	19.7 (18.5-20.9)	18.8 (17.6-19.9)
1 IADL (%)		5.0 (4.4-5.6)	9.6 (8.8-10.4)	8.7 (8.0-9.4)
2+ IADLs (%)		2.6 (2.3-3.0)	10.1 (9.3-10.8)	10.1 (9.4-10.8)

Medical costs for those covered by Medicare or older than 65 were based on Medicare Current Beneficiary Survey (MCBS) data. For these models we used ADLs and IADLs (specifically dummies for 1, 2, or 3+ ADLs, and 1 or 2+ IADLs), as variables were present and sufficiently prevalent in this age group in both surveys (**Table S4**). The prevalence of limitations in IADLs, as well as limitations in 3 or more ADLs was similar between HRS and MCBS; prevalence of limitations in 1 or 2 ADLs was slightly lower in HRS. Note that for this specific comparison exercise we excluded respondents living in nursing homes, as (I)ADLs are not reported for these respondents in MCBS (and subsequently set to 0 before the estimation of transition models). However, the MCBS cost model contains nursing home status as well as several interaction variables to account for this subgroup (**Supplementary data 2**).

Section 5. Imputation of injury due to a fall

In the HRS survey, the questions “Have you fallen down in the last two years?”, and “In that fall, did you injure yourself seriously enough to need medical treatment?” were only asked of respondents 65 years and older. Because the simulation cohort included everyone over age 50, we imputed injuries due to a fall for respondents ages 50-64 using data from the National Health Interview Survey (NHIS). This nationally-representative survey collected information on injury episodes during the past three months that were serious enough that a medical professional was consulted. Respondents were asked to describe the circumstances or events leading to the injury, and any objects, substances, or other people involved. The interviewer then entered the category that best described the cause, e.g. a fall (IRCAUS value of 5). The NHIS sample used to create a probit imputation model was restricted to ages 50 to 85; age was top-coded at 85 in NHIS. Imputation model specification and coefficients can be found in **Supplementary data 2**.

The NHIS imputation model was subsequently used to impute the probability of injury due to a fall among HRS respondents between ages 51-64. The discrepancy in recall/reporting period between HRS (past two years) and NHIS (past three months) was approached empirically. First, we determined the prevalence of injury due to a fall in the past two years among HRS respondents without missing data between the ages 65 and 75 in 2006-2016, which corresponded to a prevalence of 9.1%. We then predicted the probability of injury due to a fall using the NHIS imputation model for these HRS respondents. The probability threshold that corresponded to a similar prevalence in injury due to a fall for this age group (0.0234) was used in a logistic transformation of the predicted probability calculated with the NHIS model:

$$P_{fallinj,2\,yr} = \frac{1}{1 + e^{(-a \cdot (P_{fallinj,3\,mo} - (1.1 \cdot b))}}$$

where $P_{fallinj,2\,yr}$ is the probability of an injury due to a fall during a two-year period, a represents the steepness of the distribution around the cutoff (here set to 400 to ensure tails approached both 0 and 1 boundaries), $P_{fallinj,3\,mo}$ is the imputed probability of injury due to a fall during a three-month period using the NHIS model, and b is the calculated threshold of 0.0234.

The transformed probability for HRS respondents ages 51-64 was then compared to a uniform random draw to ultimately predict whether a respondent had experienced an injury due to a fall in the past two years. **Figure S3** shows the predicted prevalence for HRS respondents ages 51-74 using the NHIS imputation model averaged by age and survey wave (years 2006-2016), as well as the observed prevalence for HRS respondents ages 65 and older. Imputed values for ages 51-64 were used as a starting point in the simulation for these respondents, and included in the estimation of the transition model for injury due to a fall. Imputed values for ages 65-74 were only used for alignment as described here, and not for simulation or transition models.

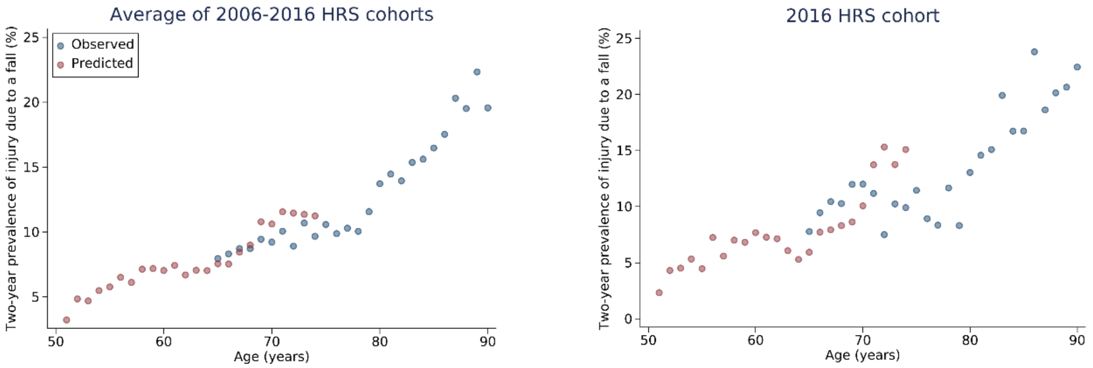

Figure S3: Imputed two-year prevalence of injury due to a fall in HRS data (in red), averaged for all years used (2006-2016; left panel), as well as for the simulation starting cohort (2016; right panel). Observed values are shown in blue, with overlapping data for ages 65-74 that were used to align and transform the predicted three-month probabilities from the imputation model.

Section 6. Supplementary Results

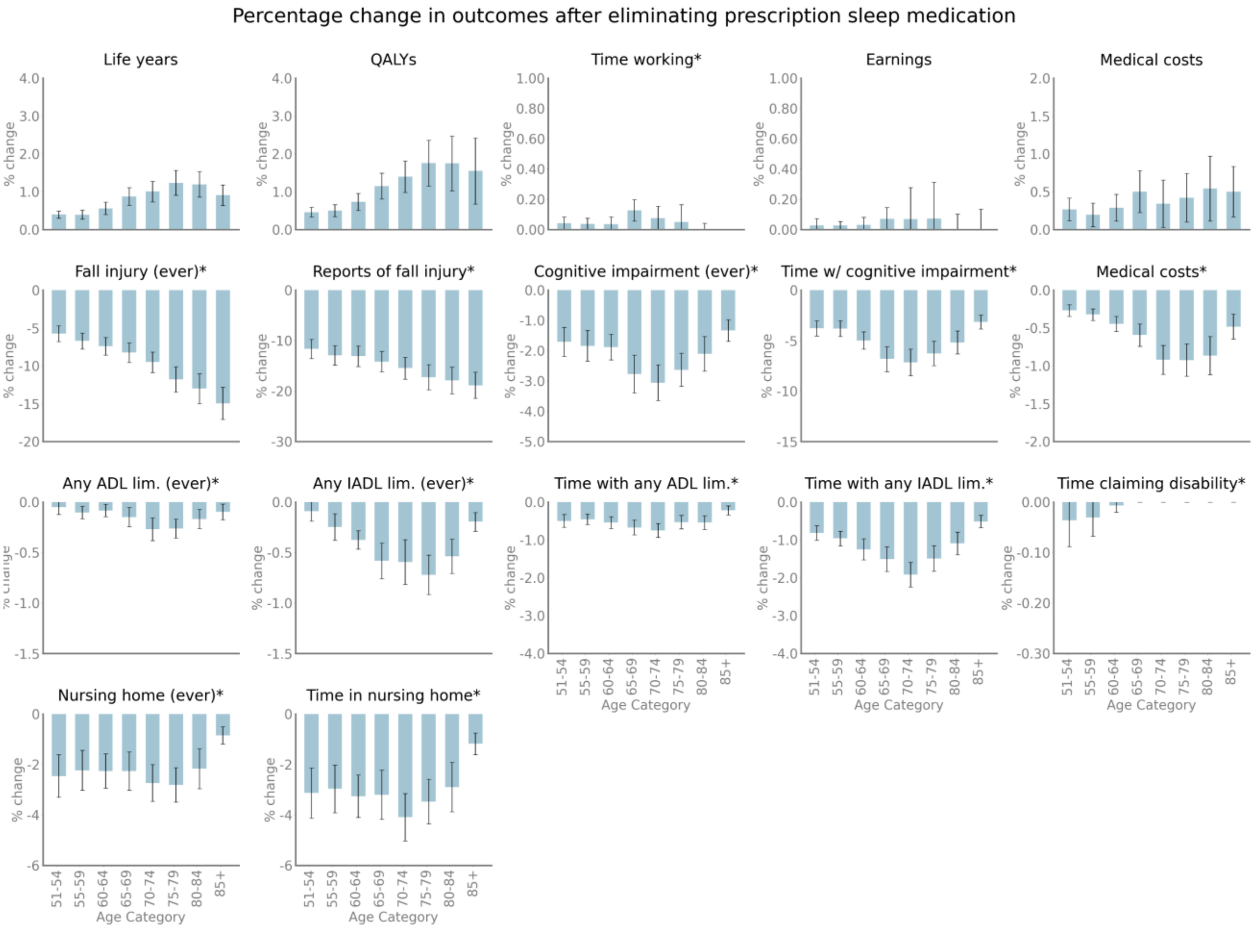

Figure S4: Percentage change in outcomes after eliminating prescription sleep medication for those who were regularly using prescription sleep medication at the start of the simulation. Change is relative to the lifetime outcome for each specific age category at the start of the simulation. All outcomes indicated with an asterisk (*) were based on simulation timepoints (waves) that were in common between the status quo and counterfactual scenarios (i.e. not accounting for extended lifespan). Other outcomes included all simulated waves. ADLs: activities of daily living; IADLs: instrumental activities of daily living; QALYs: quality-adjusted life years.

Table S5: Comparison between lifetime outcomes for all ages in status quo and counterfactual scenario, in which future prescription sleep medications were eliminated. Results are shown for those predicted to specifically use **z-drugs** (see Section 2 in this Supplementary Data file for more details). All outcomes indicated with an asterisk (*) were based on simulation timepoints (waves) that were in common between the status quo and counterfactual scenarios (i.e. not accounting for extended lifespan). The remainder of the outcomes included all simulated waves. Results expressed as reduction in years are not conditional on respective outcomes. Outcomes in the (Percentage) Difference columns are statistically significant when the confidence intervals do not include 0. ADLs: activities of daily living; IADLs: instrumental activities of daily living; QALYs: quality-adjusted life years; TICS: telephone interview for cognitive status.

	Status quo: Using z-drugs at baseline	Counterfactual: No future sleep medication	Difference	Percentage difference
Sleep medication use (% at start)	100 (100 to 100)	100 (100 to 100)	0 (0 to 0)	(0 to 0)
Sleep medication use (avg lifetime %)	49.2 (48.2 to 50.3)	0.0 (0.0 to 0.0)	-49.2 (-50.3 to -48.2)	-100.0 (-100.0 to -100.0)
Injury due to fall (% ever)*	61.8 (60.5 to 63.0)	56.8 (55.5 to 58.0)	-5.01 (-5.74 to -4.27)	-8.09 (-9.27 to -6.92)
TICS cognitive score < 12 (% ever)*	74.6 (73.7 to 75.6)	72.9 (71.8 to 74.0)	-1.72 (-2.08 to -1.35)	-2.30 (-2.79 to -1.80)
Living in nursing home (% ever)*	23.2 (21.7 to 24.7)	22.7 (21.2 to 24.2)	-0.52 (-0.67 to -0.37)	-2.24 (-2.89 to -1.59)
Any limitations in ADLs (% ever)*	80.9 (80.2 to 81.7)	80.9 (80.1 to 81.6)	-0.08 (-0.13 to -0.03)	-0.10 (-0.16 to -0.04)
Any limitations in IADLs (% ever)*	78.5 (77.6 to 79.4)	78.2 (77.3 to 79.1)	-0.27 (-0.37 to -0.17)	-0.34 (-0.47 to -0.22)
Avg nr of survey waves with any injury due to fall*	1.58 (1.50 to 1.65)	1.36 (1.29 to 1.43)	-0.21 (-0.25 to -0.18)	-13.55 (-15.53 to -11.57)
Avg years with TICS cognitive score < 12*	5.63 (5.48 to 5.79)	5.35 (5.18 to 5.52)	-0.28 (-0.33 to -0.23)	-5.06 (-5.98 to -4.14)
Avg years in nursing home*	1.18 (1.07 to 1.29)	1.14 (1.04 to 1.25)	-0.03 (-0.04 to -0.02)	-2.85 (-3.75 to -1.95)
Avg years with limitations in any ADLs*	6.03 (5.86 to 6.19)	5.99 (5.83 to 6.16)	-0.03 (-0.04 to -0.02)	-0.53 (-0.68 to -0.38)
Avg years with limitations in any IADLs*	5.44 (5.30 to 5.58)	5.37 (5.24 to 5.51)	-0.07 (-0.08 to -0.05)	-1.20 (-1.43 to -0.97)
Avg years receiving disability benefits*	1.32 (1.25 to 1.38)	1.32 (1.25 to 1.38)	-0.00 (-0.00 to 0.00)	-0.03 (-0.09 to 0.04)
Avg years working for pay*	3.35 (3.25 to 3.46)	3.36 (3.25 to 3.46)	0.00 (-0.00 to 0.00)	0.04 (-0.01 to 0.08)
Avg earnings (x 1000, $2024, disc.)*	186 (177 to 196)	187 (177 to 196)	0.03 (-0.03 to 0.10)	0.02 (-0.01 to 0.05)
Avg earnings (x 1000, $2024, disc.)	176 (167 to 185)	176 (167 to 185)	0.04 (-0.03 to 0.12)	0.02 (-0.01 to 0.06)
Avg medical costs (x 1000, $2024, disc.)*	366 (354 to 377)	364 (353 to 376)	-1.73 (-2.19 to -1.28)	-0.47 (-0.59 to -0.35)
Avg medical costs (x 1000, $2024, disc.)	377 (365 to 388)	378 (366 to 390)	1.48 (0.64 to 2.31)	0.39 (0.18 to 0.61)
Avg age at death (years)	83.2 (83.0 to 83.5)	83.4 (83.1 to 83.6)	0.12 (0.09 to 0.15)	0.14 (0.11 to 0.18)
Avg life years	19.0 (18.8 to 19.2)	19.1 (18.9 to 19.4)	0.12 (0.09 to 0.15)	0.62 (0.47 to 0.78)
Avg QALYs	11.2 (10.8 to 11.6)	11.3 (10.9 to 11.7)	0.09 (0.06 to 0.11)	0.77 (0.55 to 0.98)
Avg QALYs (x 1000, 2024$, disc.)	1,267 (1,228 to 1,306)	1,275 (1,236 to 1,314)	8.0 (5.8 to 10.1)	0.63 (0.45 to 0.81)
Cohort total life years (x 1000)	66,214 (65,104 to 67,323)	66,629 (65,480 to 67,777)	415 (309 to 521)	0.62 (0.47 to 0.78)
Cohort total QALYs (x 1000)	39,065 (37,667 to 40,464)	39,366 (37,978 to 40,754)	301 (219 to 383)	0.77 (0.55 to 0.98)
Cohort total nursing home years* (x 1000)	3,838 (3,475 to 4,201)	3,728 (3,372 to 4,084)	-110 (-146 to -74)	-2.9 (-3.7 to -2.0)
Cohort total years claiming disability* (x 1000)	4,293 (4,084 to 4,502)	4,292 (4,083 to 4,501)	-1.1 (-4.0 to 1.9)	-0.03 (-0.09 to 0.04)
Cohort total years working for pay* (x 1000)	10,935 (10,561 to 11,310)	10,940 (10,565 to 11,314)	4.3 (-0.4 to 9.1)	0.04 (-0.01 to 0.08)
Cohort total earnings (millions, $2024, disc.)*	608,260 (575,833 to 640,688)	608,368 (575,903 to 640,834)	108 (-101 to 316)	0.02 (-0.01 to 0.05)
Cohort total earnings (millions, $2024, disc.)	612,660 (580,210 to 645,111)	612,809 (580,349 to 645,270)	149 (-113 to 412)	0.02 (-0.01 to 0.06)
Cohort total medical costs (millions, $2024, disc.)*	1,191,832 (1,152,864 to 1,230,800)	1,186,181 (1,147,737 to 1,224,625)	-5,651 (-7,165 to -4,137)	-0.47 (-0.59 to -0.35)
Cohort total medical costs (millions, $2024, disc.)	1,310,627 (1,268,391 to 1,352,863)	1,315,757 (1,273,111 to 1,358,403)	5,130 (2,208 to 8,051)	0.39 (0.18 to 0.61)
Cohort total QALYs (millions, $2024, disc.)	4,409,388 (4,267,517 to 4,551,260)	4,437,147 (4,296,241 to 4,578,053)	27,759 (20,069 to 35,448)	0.63 (0.45 to 0.81)

* Includes only waves in which respondent is alive in both status quo and counterfactual scenarios

Table S6: Comparison between lifetime outcomes for all ages in status quo and counterfactual scenario, in which future prescription sleep medications were eliminated. To account for **ongoing insomnia** problems while eliminating sleep medications, we included an indicator variable for insomnia alongside sleep medication use (see Section 4 in this Supplementary Data file for more details). All outcomes indicated with an asterisk (*) were based on simulation timepoints (waves) that were in common between the status quo and counterfactual scenarios (i.e. not accounting for extended lifespan). The remainder of the outcomes included all simulated waves. Results expressed as reduction in years are not conditional on respective outcomes. Outcomes in the (Percentage) Difference columns are statistically significant when the confidence intervals do not include 0. ADLs: activities of daily living; IADLs: instrumental activities of daily living; JSS: Jenkins Sleep Scale; QALYs: quality-adjusted life years; TICS: telephone interview for cognitive status.

	**Status quo: Using sleep medication at baseline**	**Counterfactual: No future sleep medication**	**Difference**	**Percentage difference**
Sleep medication use (% at start)	100 (100 to 100)	100 (100 to 100)	0 (0 to 0)	(0 to 0)
Sleep medication use (avg lifetime %)	53.0 (52.1 to 53.8)	0.0 (0.0 to 0.0)	-53.0 (-53.8 to -52.1)	-100.0 (-100.0 to -100.0)
Sleep disturbance (JSS of 3 or more, % at start)	75.8 (75.8 to 75.8)	75.8 (75.8 to 75.8)	0.00 (0.00 to 0.00)	0.00 (0.00 to 0.00)
Injury due to fall (% ever)*	60.3 (58.7 to 61.9)	55.5 (54.2 to 56.8)	-4.85 (-5.96 to -3.73)	-8.03 (-9.76 to -6.30)
TICS cognitive score < 12 (% ever)*	75.4 (74.6 to 76.2)	73.6 (72.7 to 74.5)	-1.79 (-2.16 to -1.43)	-2.38 (-2.86 to -1.89)
Living in nursing home (% ever)*	24.6 (23.3 to 25.9)	24.0 (22.7 to 25.3)	-0.61 (-0.74 to -0.48)	-2.48 (-3.03 to -1.93)
Any limitations in ADLs (% ever)*	81.4 (80.8 to 82.0)	81.3 (80.6 to 81.9)	-0.11 (-0.15 to -0.07)	-0.13 (-0.18 to -0.09)
Any limitations in IADLs (% ever)*	79.1 (78.5 to 79.8)	78.8 (78.2 to 79.5)	-0.31 (-0.39 to -0.24)	-0.40 (-0.49 to -0.31)
Avg nr of survey waves with any injury due to fall*	1.53 (1.44 to 1.61)	1.32 (1.25 to 1.39)	-0.20 (-0.25 to -0.15)	-13.22 (-16.24 to -10.21)
Avg years with TICS cognitive score < 12*	5.61 (5.48 to 5.75)	5.29 (5.13 to 5.45)	-0.32 (-0.38 to -0.27)	-5.76 (-6.84 to -4.68)
Avg years in nursing home*	1.22 (1.13 to 1.32)	1.18 (1.09 to 1.28)	-0.04 (-0.05 to -0.03)	-3.33 (-4.06 to -2.59)
Avg years with limitations in any ADLs*	5.95 (5.81 to 6.09)	5.92 (5.77 to 6.06)	-0.03 (-0.04 to -0.02)	-0.53 (-0.68 to -0.39)
Avg years with limitations in any IADLs*	5.38 (5.26 to 5.50)	5.31 (5.20 to 5.43)	-0.07 (-0.08 to -0.06)	-1.28 (-1.51 to -1.06)
Avg years receiving disability benefits*	1.27 (1.23 to 1.31)	1.27 (1.23 to 1.31)	-0.00 (-0.00 to -0.00)	-0.03 (-0.06 to -0.00)
Avg years working for pay*	3.12 (3.04 to 3.19)	3.12 (3.04 to 3.20)	0.00 (0.00 to 0.00)	0.05 (0.03 to 0.07)
Avg earnings (x 1000, $2024, disc.)*	172 (165 to 179)	172 (165 to 179)	0.03 (0.00 to 0.07)	0.02 (0.00 to 0.04)
Avg earnings (x 1000, $2024, disc.)	160 (153 to 166)	160 (154 to 166)	0.06 (0.02 to 0.10)	0.04 (0.01 to 0.06)
Avg medical costs (x 1000, $2024, disc.)*	360 (350 to 371)	358 (348 to 369)	-2.05 (-2.45 to -1.65)	-0.57 (-0.68 to -0.46)
Avg medical costs (x 1000, $2024, disc.)	368 (357 to 378)	369 (358 to 380)	1.25 (0.58 to 1.92)	0.34 (0.16 to 0.52)
Avg age at death (years)	83.8 (83.6 to 83.9)	83.9 (83.7 to 84.0)	0.12 (0.10 to 0.15)	0.15 (0.11 to 0.18)
Avg life years	18.1 (17.9 to 18.2)	18.2 (18.1 to 18.4)	0.12 (0.10 to 0.15)	0.68 (0.53 to 0.83)
Avg QALYs	10.6 (10.2 to 10.9)	10.6 (10.3 to 11.0)	0.09 (0.07 to 0.12)	0.88 (0.61 to 1.15)
Avg QALYs (x 1000, 2024$, disc.)	1,194 (1,154 to 1,233)	1,202 (1,163 to 1,241)	8.5 (6.0 to 11.0)	0.71 (0.49 to 0.93)
Cohort total life years (x 1000)	276,667 (274,258 to 279,076)	278,550 (276,123 to 280,977)	1,883 (1,473 to 2,293)	0.68 (0.53 to 0.83)
Cohort total QALYs (x 1000)	161,414 (155,401 to 167,427)	162,835 (156,914 to 168,756)	1,421 (1,008 to 1,834)	0.88 (0.61 to 1.15)
Cohort total nursing home years* (x 1000)	17,244 (15,893 to 18,594)	16,672 (15,326 to 18,018)	-572 (-695 to -449)	-3.3 (-4.1 to -2.6)
Cohort total years claiming disability* (x 1000)	17,851 (17,284 to 18,418)	17,846 (17,278 to 18,413)	-5.2 (-10.1 to -0.4)	-0.03 (-0.06 to -0.00)
Cohort total years working for pay* (x 1000)	43,930 (42,842 to 45,018)	43,953 (42,865 to 45,041)	23.4 (14.3 to 32.5)	0.05 (0.03 to 0.07)
Cohort total earnings (millions, $2024, disc.)*	2,427,271 (2,329,630 to 2,524,911)	2,427,762 (2,330,087 to 2,525,437)	491 (50 to 933)	0.02 (0.00 to 0.04)
Cohort total earnings (millions, $2024, disc.)	2,445,053 (2,346,679 to 2,543,427)	2,445,954 (2,347,589 to 2,544,320)	901 (252 to 1,551)	0.04 (0.01 to 0.06)
Cohort total medical costs (millions, $2024, disc.)*	5,077,702 (4,928,930 to 5,226,473)	5,048,825 (4,901,172 to 5,196,478)	-28,877 (-34,514 to -23,239)	-0.57 (-0.68 to -0.46)
Cohort total medical costs (millions, $2024, disc.)	5,620,914 (5,459,240 to 5,782,587)	5,640,010 (5,476,153 to 5,803,868)	19,097 (8,832 to 29,361)	0.34 (0.16 to 0.52)
Cohort total QALYs (millions, $2024, disc.)	18,252,132 (17,647,460 to 18,856,804)	18,382,132 (17,786,652 to 18,977,612)	130,000 (91,454 to 168,547)	0.71 (0.49 to 0.93)

* Includes only waves in which respondent is alive in both status quo and counterfactual scenarios

Table S7a: Comparison between lifetime outcomes in status quo and counterfactual scenario, in which future prescription sleep medications were eliminated. The cohort sample consisted of all simulants **ages 51-54** at the start of the simulation who indicated regularly using prescription sleep medication. All outcomes indicated with an asterisk (*) were based on simulation timepoints (waves) that were in common between the status quo and counterfactual scenarios (i.e. not accounting for extended lifespan). The remainder of the outcomes included all simulated waves. Results expressed as reduction in years are not conditional on respective outcomes. Outcomes in the (Percentage) Difference columns are statistically significant when the confidence intervals do not include 0. ADLs: activities of daily living; IADLs: instrumental activities of daily living; QALYs: quality-adjusted life years; TICS: telephone interview for cognitive status

	Status quo: Using sleep medication at baseline	Counterfactual: No future sleep medication	Difference	Percentage difference
Sleep medication use (% at start)	100 (100 to 100)	100 (100 to 100)	0 (0 to 0)	(0 to 0)
Sleep medication use (avg lifetime %)	37.2 (35.8 to 38.6)	0.0 (0.0 to 0.0)	-37.2 (-38.6 to -35.8)	-100.0 (-100.0 to -100.0)
Injury due to fall (% ever)*	69.3 (68.1 to 70.5)	65.3 (64.0 to 66.6)	-3.99 (-4.73 to -3.25)	-5.75 (-6.80 to -4.69)
TICS cognitive score < 12 (% ever)*	68.0 (66.6 to 69.4)	66.9 (65.4 to 68.4)	-1.16 (-1.48 to -0.84)	-1.71 (-2.19 to -1.23)
Living in nursing home (% ever)*	20.0 (18.4 to 21.7)	19.5 (17.9 to 21.2)	-0.49 (-0.66 to -0.33)	-2.46 (-3.30 to -1.62)
Any limitations in ADLs (% ever)*	85.4 (84.5 to 86.4)	85.4 (84.4 to 86.3)	-0.04 (-0.10 to 0.02)	-0.05 (-0.12 to 0.02)
Any limitations in IADLs (% ever)*	84.3 (83.5 to 85.2)	84.2 (83.4 to 85.1)	-0.08 (-0.16 to 0.00)	-0.09 (-0.19 to 0.00)
Avg nr of survey waves with any injury due to fall*	2.13 (2.03 to 2.22)	1.88 (1.79 to 1.97)	-0.25 (-0.29 to -0.20)	-11.65 (-13.58 to -9.73)
Avg years with TICS cognitive score < 12*	6.35 (6.14 to 6.56)	6.11 (5.88 to 6.33)	-0.24 (-0.28 to -0.20)	-3.77 (-4.52 to -3.03)
Avg years in nursing home*	1.06 (0.93 to 1.20)	1.03 (0.90 to 1.17)	-0.03 (-0.04 to -0.02)	-3.13 (-4.12 to -2.14)
Avg years with limitations in any ADLs*	7.82 (7.56 to 8.08)	7.78 (7.53 to 8.04)	-0.04 (-0.05 to -0.03)	-0.50 (-0.67 to -0.33)
Avg years with limitations in any IADLs*	6.92 (6.69 to 7.16)	6.87 (6.64 to 7.10)	-0.06 (-0.07 to -0.04)	-0.82 (-1.01 to -0.62)
Avg years receiving disability benefits*	3.57 (3.40 to 3.73)	3.57 (3.40 to 3.73)	-0.00 (-0.00 to 0.00)	-0.04 (-0.09 to 0.02)
Avg years working for pay*	7.42 (7.21 to 7.63)	7.43 (7.21 to 7.64)	0.00 (-0.00 to 0.01)	0.04 (-0.00 to 0.08)
Avg earnings (x 1000, $2024, disc.)*	414 (392 to 437)	415 (392 to 437)	0.08 (-0.08 to 0.23)	0.02 (-0.02 to 0.05)
Avg earnings (x 1000, $2024, disc.)	417 (395 to 439)	417 (395 to 439)	0.12 (-0.07 to 0.31)	0.03 (-0.02 to 0.07)
Avg medical costs (x 1000, $2024, disc.)*	399 (385 to 412)	398 (384 to 411)	-1.07 (-1.39 to -0.76)	-0.27 (-0.34 to -0.19)
Avg medical costs (x 1000, $2024, disc.)	422 (408 to 436)	424 (409 to 438)	1.13 (0.48 to 1.78)	0.27 (0.12 to 0.42)
Avg age at death (years)	80.1 (79.7 to 80.5)	80.2 (79.8 to 80.6)	0.12 (0.09 to 0.14)	0.14 (0.11 to 0.18)
Avg life years	29.1 (28.7 to 29.5)	29.2 (28.8 to 29.6)	0.12 (0.09 to 0.14)	0.40 (0.30 to 0.50)
Avg QALYs	18.1 (17.4 to 18.8)	18.2 (17.5 to 18.8)	0.08 (0.06 to 0.11)	0.46 (0.33 to 0.59)
Avg QALYs (x 1000, 2024$, disc.)	1,872 (1,809 to 1,934)	1,877 (1,815 to 1,940)	5.6 (4.0 to 7.2)	0.30 (0.21 to 0.38)
Cohort total life years (x 1000)	62,255 (61,402 to 63,108)	62,503 (61,656 to 63,349)	248 (187 to 309)	0.40 (0.30 to 0.50)
Cohort total QALYs (x 1000)	38,730 (37,306 to 40,155)	38,909 (37,493 to 40,326)	179 (131 to 227)	0.46 (0.33 to 0.59)
Cohort total nursing home years* (x 1000)	2,277 (1,982 to 2,572)	2,209 (1,920 to 2,498)	-67 (-92 to -43)	-3.1 (-4.1 to -2.1)
Cohort total years claiming disability* (x 1000)	7,637 (7,286 to 7,988)	7,635 (7,283 to 7,987)	-2.7 (-6.7 to 1.3)	-0.04 (-0.09 to 0.02)
Cohort total years working for pay* (x 1000)	15,887 (15,435 to 16,339)	15,894 (15,440 to 16,347)	6.6 (-0.3 to 13.5)	0.04 (-0.00 to 0.08)
Cohort total earnings (millions, $2024, disc.)*	887,135 (839,625 to 934,644)	887,298 (839,733 to 934,862)	163 (-164 to 490)	0.02 (-0.02 to 0.05)
Cohort total earnings (millions, $2024, disc.)	892,592 (844,844 to 940,339)	892,844 (845,051 to 940,636)	252 (-158 to 662)	0.03 (-0.02 to 0.07)
Cohort total medical costs (millions, $2024, disc.)*	853,586 (824,840 to 882,332)	851,288 (822,641 to 879,935)	-2,298 (-2,977 to -1,619)	-0.27 (-0.34 to -0.19)
Cohort total medical costs (millions, $2024, disc.)	904,111 (874,233 to 933,990)	906,525 (876,345 to 936,706)	2,414 (1,025 to 3,803)	0.27 (0.12 to 0.42)
Cohort total QALYs (millions, $2024, disc.)	4,006,408 (3,872,598 to 4,140,218)	4,018,346 (3,885,065 to 4,151,626)	11,937 (8,536 to 15,339)	0.30 (0.21 to 0.38)

* Includes only waves in which respondent is alive in both status quo and counterfactual scenarios

Table S7b: age 55-59 at the start of the simulation.

	Status quo: Using sleep medication at baseline	Counterfactual: No future sleep medication	Difference	Percentage difference
Sleep medication use (% at start)	100 (100 to 100)	100 (100 to 100)	0 (0 to 0)	(0 to 0)
Sleep medication use (avg lifetime %)	40.7 (39.5 to 41.9)	0.0 (0.0 to 0.0)	-40.7 (-41.9 to -39.5)	-100.0 (-100.0 to -100.0)
Injury due to fall (% ever)*	64.2 (63.0 to 65.4)	59.9 (58.6 to 61.1)	-4.32 (-5.01 to -3.62)	-6.72 (-7.78 to -5.66)
TICS cognitive score < 12 (% ever)*	70.2 (68.9 to 71.5)	68.9 (67.5 to 70.3)	-1.29 (-1.65 to -0.94)	-1.84 (-2.35 to -1.33)
Living in nursing home (% ever)*	19.2 (17.6 to 20.9)	18.8 (17.2 to 20.5)	-0.42 (-0.57 to -0.27)	-2.23 (-3.03 to -1.44)
Any limitations in ADLs (% ever)*	84.0 (83.2 to 84.9)	84.0 (83.1 to 84.8)	-0.09 (-0.14 to -0.03)	-0.10 (-0.17 to -0.04)
Any limitations in IADLs (% ever)*	79.7 (78.9 to 80.6)	79.5 (78.7 to 80.4)	-0.20 (-0.30 to -0.09)	-0.25 (-0.38 to -0.12)
Avg nr of survey waves with any injury due to fall*	1.75 (1.67 to 1.83)	1.52 (1.45 to 1.60)	-0.23 (-0.26 to -0.19)	-12.92 (-14.87 to -10.98)
Avg years with TICS cognitive score < 12*	6.27 (6.08 to 6.47)	6.03 (5.82 to 6.25)	-0.24 (-0.29 to -0.19)	-3.81 (-4.59 to -3.03)
Avg years in nursing home*	0.99 (0.88 to 1.11)	0.96 (0.85 to 1.08)	-0.03 (-0.04 to -0.02)	-2.97 (-3.91 to -2.02)
Avg years with limitations in any ADLs*	7.05 (6.84 to 7.26)	7.02 (6.80 to 7.23)	-0.03 (-0.04 to -0.02)	-0.46 (-0.59 to -0.32)
Avg years with limitations in any IADLs*	6.08 (5.91 to 6.26)	6.02 (5.85 to 6.20)	-0.06 (-0.07 to -0.05)	-0.96 (-1.16 to -0.76)
Avg years receiving disability benefits*	2.60 (2.51 to 2.68)	2.60 (2.51 to 2.68)	-0.00 (-0.00 to 0.00)	-0.03 (-0.07 to 0.01)
Avg years working for pay*	5.21 (5.06 to 5.37)	5.21 (5.06 to 5.37)	0.00 (-0.00 to 0.00)	0.04 (0.00 to 0.07)
Avg earnings (x 1000, $2024, disc.)*	308 (295 to 322)	308 (295 to 322)	0.04 (-0.01 to 0.09)	0.01 (-0.00 to 0.03)
Avg earnings (x 1000, $2024, disc.)	302 (289 to 315)	302 (289 to 315)	0.08 (-0.01 to 0.16)	0.03 (-0.00 to 0.05)
Avg medical costs (x 1000, $2024, disc.)*	382 (370 to 394)	381 (369 to 393)	-1.24 (-1.54 to -0.93)	-0.32 (-0.40 to -0.25)
Avg medical costs (x 1000, $2024, disc.)	398 (385 to 410)	398 (386 to 411)	0.76 (0.13 to 1.39)	0.19 (0.04 to 0.35)
Avg age at death (years)	80.3 (80.1 to 80.6)	80.4 (80.2 to 80.7)	0.10 (0.07 to 0.13)	0.12 (0.09 to 0.16)
Avg life years	24.9 (24.6 to 25.2)	25.0 (24.7 to 25.2)	0.10 (0.07 to 0.13)	0.40 (0.28 to 0.51)
Avg QALYs	15.3 (14.8 to 15.8)	15.4 (14.8 to 15.9)	0.08 (0.05 to 0.10)	0.50 (0.34 to 0.66)
Avg QALYs (x 1000, 2024$, disc.)	1,645 (1,593 to 1,697)	1,651 (1,599 to 1,702)	5.9 (4.1 to 7.6)	0.36 (0.25 to 0.47)
Cohort total life years (x 1000)	68,807 (68,002 to 69,613)	69,079 (68,277 to 69,881)	272 (191 to 353)	0.40 (0.28 to 0.51)
Cohort total QALYs (x 1000)	42,284 (40,827 to 43,740)	42,495 (41,052 to 43,939)	212 (148 to 276)	0.50 (0.34 to 0.66)
Cohort total nursing home years* (x 1000)	2,669 (2,355 to 2,982)	2,588 (2,276 to 2,900)	-81 (-105 to -56)	-3.0 (-3.9 to -2.0)
Cohort total years claiming disability* (x 1000)	6,989 (6,764 to 7,214)	6,987 (6,761 to 7,212)	-2.2 (-4.7 to 0.4)	-0.03 (-0.07 to 0.01)
Cohort total years working for pay* (x 1000)	14,027 (13,608 to 14,447)	14,033 (13,613 to 14,453)	5.1 (-0.1 to 10.4)	0.04 (0.00 to 0.07)
Cohort total earnings (millions, $2024, disc.)*	829,879 (794,218 to 865,540)	829,996 (794,298 to 865,694)	117 (-20 to 254)	0.01 (-0.00 to 0.03)
Cohort total earnings (millions, $2024, disc.)	835,266 (799,488 to 871,044)	835,482 (799,701 to 871,263)	216 (-19 to 451)	0.03 (-0.00 to 0.05)
Cohort total medical costs (millions, $2024, disc.)*	1,027,768 (994,970 to 1,060,566)	1,024,443 (991,745 to 1,057,141)	-3,325 (-4,137 to -2,513)	-0.32 (-0.40 to -0.25)
Cohort total medical costs (millions, $2024, disc.)	1,100,343 (1,065,835 to 1,134,852)	1,102,456 (1,067,662 to 1,137,251)	2,113 (370 to 3,856)	0.19 (0.04 to 0.35)
Cohort total QALYs (millions, $2024, disc.)	4,552,416 (4,409,117 to 4,695,716)	4,568,667 (4,426,405 to 4,710,929)	16,251 (11,367 to 21,135)	0.36 (0.25 to 0.47)

* Includes only waves in which respondent is alive in both status quo and counterfactual scenarios

Table S7c: age 60-64 at the start of the simulation.

	Status quo: Using sleep medication at baseline	Counterfactual: No future sleep medication	Difference	Percentage difference
Sleep medication use (% at start)	100 (100 to 100)	100 (100 to 100)	0 (0 to 0)	(0 to 0)
Sleep medication use (avg lifetime %)	45.7 (44.4 to 47.0)	0.0 (0.0 to 0.0)	-45.7 (-47.0 to -44.4)	-100.0 (-100.0 to -100.0)
Injury due to fall (% ever)*	61.0 (59.6 to 62.4)	56.5 (55.0 to 58.0)	-4.52 (-5.24 to -3.80)	-7.41 (-8.59 to -6.24)
TICS cognitive score < 12 (% ever)*	74.5 (73.6 to 75.3)	73.0 (72.1 to 74.0)	-1.41 (-1.72 to -1.09)	-1.88 (-2.31 to -1.46)
Living in nursing home (% ever)*	20.3 (18.8 to 21.7)	19.8 (18.4 to 21.2)	-0.46 (-0.61 to -0.32)	-2.26 (-2.94 to -1.57)
Any limitations in ADLs (% ever)*	82.8 (82.0 to 83.6)	82.7 (81.9 to 83.6)	-0.07 (-0.12 to -0.02)	-0.08 (-0.14 to -0.02)
Any limitations in IADLs (% ever)*	77.7 (76.9 to 78.6)	77.5 (76.6 to 78.4)	-0.29 (-0.36 to -0.22)	-0.37 (-0.47 to -0.28)
Avg nr of survey waves with any injury due to fall*	1.59 (1.51 to 1.67)	1.38 (1.31 to 1.46)	-0.21 (-0.24 to -0.17)	-13.08 (-15.12 to -11.04)
Avg years with TICS cognitive score < 12*	6.02 (5.87 to 6.16)	5.72 (5.55 to 5.88)	-0.30 (-0.35 to -0.25)	-4.99 (-5.85 to -4.12)
Avg years in nursing home*	1.02 (0.91 to 1.13)	0.99 (0.88 to 1.10)	-0.03 (-0.04 to -0.02)	-3.26 (-4.10 to -2.42)
Avg years with limitations in any ADLs*	6.38 (6.19 to 6.57)	6.34 (6.15 to 6.53)	-0.03 (-0.04 to -0.02)	-0.54 (-0.70 to -0.38)
Avg years with limitations in any IADLs*	5.55 (5.40 to 5.70)	5.48 (5.33 to 5.63)	-0.07 (-0.09 to -0.05)	-1.25 (-1.53 to -0.97)
Avg years receiving disability benefits*	1.29 (1.27 to 1.31)	1.29 (1.27 to 1.31)	-0.00 (-0.00 to 0.00)	-0.01 (-0.02 to 0.01)
Avg years working for pay*	3.25 (3.13 to 3.37)	3.25 (3.14 to 3.37)	0.00 (-0.00 to 0.00)	0.04 (-0.01 to 0.08)
Avg earnings (x 1000, $2024, disc.)*	184 (175 to 194)	184 (175 to 194)	0.04 (-0.00 to 0.08)	0.02 (0.00 to 0.04)
Avg earnings (x 1000, $2024, disc.)	177 (167 to 186)	177 (167 to 186)	0.05 (-0.03 to 0.14)	0.03 (-0.02 to 0.08)
Avg medical costs (x 1000, $2024, disc.)*	372 (359 to 385)	370 (357 to 383)	-1.67 (-2.06 to -1.28)	-0.45 (-0.55 to -0.35)
Avg medical costs (x 1000, $2024, disc.)	384 (371 to 397)	385 (372 to 398)	1.11 (0.41 to 1.81)	0.29 (0.11 to 0.47)
Avg age at death (years)	80.8 (80.6 to 81.1)	81.0 (80.7 to 81.2)	0.12 (0.08 to 0.15)	0.14 (0.10 to 0.18)
Avg life years	20.5 (20.2 to 20.7)	20.6 (20.3 to 20.8)	0.12 (0.08 to 0.15)	0.56 (0.40 to 0.72)
Avg QALYs	12.1 (11.7 to 12.6)	12.2 (11.8 to 12.6)	0.09 (0.06 to 0.11)	0.73 (0.51 to 0.95)
Avg QALYs (x 1000, 2024$, disc.)	1,375 (1,330 to 1,419)	1,383 (1,339 to 1,427)	7.9 (5.5 to 10.2)	0.57 (0.40 to 0.75)
Cohort total life years (x 1000)	52,188 (51,600 to 52,776)	52,482 (51,889 to 53,075)	294 (211 to 377)	0.56 (0.40 to 0.72)
Cohort total QALYs (x 1000)	30,948 (29,849 to 32,047)	31,175 (30,089 to 32,260)	227 (160 to 293)	0.73 (0.51 to 0.95)
Cohort total nursing home years* (x 1000)	2,470 (2,196 to 2,744)	2,391 (2,123 to 2,659)	-79 (-101 to -57)	-3.3 (-4.1 to -2.4)
Cohort total years claiming disability* (x 1000)	3,127 (3,070 to 3,184)	3,127 (3,069 to 3,184)	-0.2 (-0.6 to 0.2)	-0.01 (-0.02 to 0.01)
Cohort total years working for pay* (x 1000)	7,880 (7,599 to 8,161)	7,883 (7,603 to 8,163)	3.0 (-0.6 to 6.6)	0.04 (-0.01 to 0.08)
Cohort total earnings (millions, $2024, disc.)*	447,040 (423,250 to 470,830)	447,142 (423,335 to 470,948)	102 (-1 to 205)	0.02 (0.00 to 0.04)
Cohort total earnings (millions, $2024, disc.)	450,435 (426,308 to 474,561)	450,575 (426,483 to 474,666)	140 (-88 to 368)	0.03 (-0.02 to 0.08)
Cohort total medical costs (millions, $2024, disc.)*	901,831 (870,522 to 933,140)	897,782 (866,748 to 928,816)	-4,049 (-5,004 to -3,093)	-0.45 (-0.55 to -0.35)
Cohort total medical costs (millions, $2024, disc.)	979,879 (946,841 to 1,012,917)	982,714 (949,579 to 1,015,850)	2,835 (1,042 to 4,628)	0.29 (0.11 to 0.47)
Cohort total QALYs (millions, $2024, disc.)	3,507,426 (3,394,291 to 3,620,561)	3,527,506 (3,415,624 to 3,639,389)	20,080 (14,100 to 26,061)	0.57 (0.40 to 0.75)

* Includes only waves in which respondent is alive in both status quo and counterfactual scenarios

Table S7d: age 65-69 at the start of the simulation.

	Status quo: Using sleep medication at baseline	Counterfactual: No future sleep medication	Difference	Percentage difference
Sleep medication use (% at start)	100 (100 to 100)	100 (100 to 100)	0 (0 to 0)	(0 to 0)
Sleep medication use (avg lifetime %)	47.8 (46.8 to 48.8)	0.0 (0.0 to 0.0)	-47.8 (-48.8 to -46.8)	-100.0 (-100.0 to -100.0)
Injury due to fall (% ever)*	60.8 (59.2 to 62.4)	55.8 (54.4 to 57.2)	-5.02 (-5.86 to -4.18)	-8.24 (-9.54 to -6.94)
TICS cognitive score < 12 (% ever)*	77.7 (76.8 to 78.5)	75.5 (74.5 to 76.5)	-2.16 (-2.64 to -1.67)	-2.77 (-3.40 to -2.15)
Living in nursing home (% ever)*	22.1 (20.6 to 23.7)	21.6 (20.1 to 23.1)	-0.51 (-0.69 to -0.33)	-2.26 (-3.02 to -1.50)
Any limitations in ADLs (% ever)*	78.7 (77.7 to 79.7)	78.6 (77.6 to 79.6)	-0.12 (-0.19 to -0.04)	-0.15 (-0.24 to -0.05)
Any limitations in IADLs (% ever)*	75.5 (74.3 to 76.8)	75.1 (73.8 to 76.4)	-0.44 (-0.57 to -0.30)	-0.58 (-0.76 to -0.40)
Avg nr of survey waves with any injury due to fall*	1.48 (1.40 to 1.56)	1.27 (1.20 to 1.34)	-0.21 (-0.25 to -0.17)	-14.19 (-16.22 to -12.15)
Avg years with TICS cognitive score < 12*	5.60 (5.44 to 5.75)	5.21 (5.04 to 5.39)	-0.38 (-0.45 to -0.31)	-6.83 (-8.07 to -5.58)
Avg years in nursing home*	1.13 (1.02 to 1.24)	1.09 (0.98 to 1.20)	-0.04 (-0.05 to -0.02)	-3.20 (-4.17 to -2.23)
Avg years with limitations in any ADLs*	5.59 (5.42 to 5.77)	5.56 (5.38 to 5.73)	-0.04 (-0.05 to -0.03)	-0.67 (-0.87 to -0.48)
Avg years with limitations in any IADLs*	5.03 (4.86 to 5.21)	4.96 (4.79 to 5.12)	-0.08 (-0.09 to -0.06)	-1.51 (-1.83 to -1.18)
Avg years receiving disability benefits*	0.05 (0.05 to 0.05)	0.05 (0.05 to 0.05)	0.00 (0.00 to 0.00)	0.00 (0.00 to 0.00)
Avg years working for pay*	1.85 (1.77 to 1.92)	1.85 (1.77 to 1.92)	0.00 (0.00 to 0.00)	0.13 (0.06 to 0.20)
Avg earnings (x 1000, $2024, disc.)*	93 (90 to 97)	93 (90 to 97)	0.03 (-0.01 to 0.06)	0.03 (-0.01 to 0.07)
Avg earnings (x 1000, $2024, disc.)	90 (87 to 94)	90 (87 to 94)	0.07 (0.00 to 0.13)	0.07 (-0.00 to 0.14)
Avg medical costs (x 1000, $2024, disc.)*	367 (354 to 381)	365 (352 to 378)	-2.18 (-2.76 to -1.60)	-0.59 (-0.74 to -0.44)
Avg medical costs (x 1000, $2024, disc.)	387 (374 to 400)	389 (375 to 403)	1.92 (0.81 to 3.04)	0.50 (0.22 to 0.78)
Avg age at death (years)	83.4 (83.2 to 83.6)	83.6 (83.4 to 83.8)	0.16 (0.11 to 0.20)	0.19 (0.14 to 0.24)
Avg life years	17.9 (17.7 to 18.1)	18.0 (17.8 to 18.2)	0.16 (0.11 to 0.20)	0.88 (0.64 to 1.11)
Avg QALYs	10.5 (10.1 to 10.9)	10.6 (10.2 to 11.0)	0.12 (0.09 to 0.15)	1.15 (0.81 to 1.49)
Avg QALYs (x 1000, 2024$, disc.)	1,248 (1,208 to 1,288)	1,260 (1,221 to 1,299)	12.0 (8.6 to 15.4)	0.96 (0.68 to 1.25)
Cohort total life years (x 1000)	39,792 (39,356 to 40,228)	40,139 (39,678 to 40,600)	347 (254 to 440)	0.88 (0.64 to 1.11)
Cohort total QALYs (x 1000)	23,301 (22,423 to 24,180)	23,569 (22,705 to 24,433)	268 (191 to 344)	1.15 (0.81 to 1.49)
Cohort total nursing home years* (x 1000)	2,398 (2,158 to 2,639)	2,321 (2,087 to 2,554)	-78 (-103 to -52)	-3.2 (-4.2 to -2.2)
Cohort total years claiming disability* (x 1000)	98 (98 to 98)	98 (98 to 98)	0.0 (0.0 to 0.0)	0.00 (0.00 to 0.00)
Cohort total years working for pay* (x 1000)	3,923 (3,765 to 4,082)	3,928 (3,770 to 4,086)	4.8 (2.0 to 7.6)	0.13 (0.06 to 0.20)
Cohort total earnings (millions, $2024, disc.)*	198,658 (190,996 to 206,320)	198,715 (191,059 to 206,371)	57 (-17 to 130)	0.03 (-0.01 to 0.07)
Cohort total earnings (millions, $2024, disc.)	200,629 (192,947 to 208,310)	200,774 (193,147 to 208,401)	146 (4 to 287)	0.07 (-0.00 to 0.14)
Cohort total medical costs (millions, $2024, disc.)*	780,902 (752,809 to 808,996)	776,273 (748,556 to 803,989)	-4,629 (-5,867 to -3,392)	-0.59 (-0.74 to -0.44)
Cohort total medical costs (millions, $2024, disc.)	861,590 (831,886 to 891,294)	865,874 (835,425 to 896,322)	4,284 (1,806 to 6,761)	0.50 (0.22 to 0.78)
Cohort total QALYs (millions, $2024, disc.)	2,778,070 (2,689,419 to 2,866,722)	2,804,736 (2,717,629 to 2,891,842)	26,665 (19,050 to 34,280)	0.96 (0.68 to 1.25)

* Includes only waves in which respondent is alive in both status quo and counterfactual scenarios

Table S7e: age 70-74 at the start of the simulation.

	Status quo: Using sleep medication at baseline	Counterfactual: No future sleep medication	Difference	Percentage difference
Sleep medication use (% at start)	100 (100 to 100)	100 (100 to 100)	0 (0 to 0)	(0 to 0)
Sleep medication use (avg lifetime %)	53.0 (52.0 to 54.1)	0.0 (0.0 to 0.0)	-53.0 (-54.1 to -52.0)	-100.0 (-100.0 to -100.0)
Injury due to fall (% ever)*	60.1 (58.4 to 61.9)	54.4 (52.9 to 55.9)	-5.73 (-6.61 to -4.85)	-9.51 (-10.88 to -8.15)
TICS cognitive score < 12 (% ever)*	77.6 (76.3 to 78.8)	75.2 (73.8 to 76.6)	-2.38 (-2.82 to -1.93)	-3.06 (-3.65 to -2.47)
Living in nursing home (% ever)*	26.9 (25.1 to 28.6)	26.1 (24.4 to 27.8)	-0.74 (-0.95 to -0.53)	-2.74 (-3.47 to -2.01)
Any limitations in ADLs (% ever)*	75.8 (74.8 to 76.9)	75.6 (74.6 to 76.7)	-0.20 (-0.29 to -0.12)	-0.27 (-0.38 to -0.16)
Any limitations in IADLs (% ever)*	73.7 (72.4 to 74.9)	73.2 (72.0 to 74.5)	-0.44 (-0.60 to -0.27)	-0.59 (-0.82 to -0.37)
Avg nr of survey waves with any injury due to fall*	1.38 (1.30 to 1.46)	1.17 (1.10 to 1.24)	-0.21 (-0.25 to -0.18)	-15.46 (-17.62 to -13.30)
Avg years with TICS cognitive score < 12*	5.29 (5.12 to 5.45)	4.91 (4.73 to 5.09)	-0.38 (-0.45 to -0.31)	-7.16 (-8.47 to -5.85)
Avg years in nursing home*	1.39 (1.27 to 1.52)	1.33 (1.21 to 1.45)	-0.06 (-0.07 to -0.04)	-4.09 (-5.02 to -3.15)
Avg years with limitations in any ADLs*	5.05 (4.91 to 5.20)	5.01 (4.87 to 5.16)	-0.04 (-0.05 to -0.03)	-0.75 (-0.93 to -0.57)
Avg years with limitations in any IADLs*	4.69 (4.54 to 4.84)	4.60 (4.46 to 4.74)	-0.09 (-0.11 to -0.07)	-1.92 (-2.25 to -1.59)
Avg years receiving disability benefits*	0.00 (0.00 to 0.00)	0.00 (0.00 to 0.00)	0.00 (0.00 to 0.00)	0.00 (0.00 to 0.00)
Avg years working for pay*	1.05 (1.01 to 1.09)	1.05 (1.01 to 1.09)	0.00 (-0.00 to 0.00)	0.07 (-0.01 to 0.15)
Avg earnings (x 1000, $2024, disc.)*	30 (29 to 32)	30 (29 to 32)	0.01 (-0.03 to 0.04)	0.03 (-0.07 to 0.13)
Avg earnings (x 1000, $2024, disc.)	29 (27 to 31)	29 (27 to 31)	0.02 (-0.04 to 0.08)	0.07 (-0.14 to 0.28)
Avg medical costs (x 1000, $2024, disc.)*	364 (350 to 378)	361 (347 to 375)	-3.36 (-4.11 to -2.61)	-0.92 (-1.11 to -0.73)
Avg medical costs (x 1000, $2024, disc.)	381 (367 to 394)	382 (368 to 396)	1.27 (0.06 to 2.48)	0.34 (0.03 to 0.65)
Avg age at death (years)	85.1 (84.9 to 85.3)	85.2 (85.0 to 85.4)	0.15 (0.11 to 0.19)	0.17 (0.13 to 0.22)
Avg life years	14.7 (14.5 to 14.9)	14.9 (14.7 to 15.1)	0.15 (0.11 to 0.19)	1.00 (0.73 to 1.28)
Avg QALYs	8.2 (7.9 to 8.6)	8.4 (8.0 to 8.7)	0.11 (0.08 to 0.15)	1.40 (0.98 to 1.81)
Avg QALYs (x 1000, 2024$, disc.)	1,032 (991 to 1,073)	1,044 (1,004 to 1,084)	12.1 (8.6 to 15.5)	1.17 (0.82 to 1.52)
Cohort total life years (x 1000)	24,341 (24,029 to 24,654)	24,585 (24,258 to 24,912)	243 (177 to 310)	1.00 (0.73 to 1.28)
Cohort total QALYs (x 1000)	13,629 (12,994 to 14,264)	13,819 (13,196 to 14,442)	190 (136 to 243)	1.40 (0.98 to 1.81)
Cohort total nursing home years* (x 1000)	2,157 (1,963 to 2,351)	2,068 (1,883 to 2,254)	-89 (-110 to -67)	-4.1 (-5.0 to -3.2)
Cohort total years claiming disability* (x 1000)	0 (0 to 0)	0 (0 to 0)	0.0 (0.0 to 0.0)	0.00 (0.00 to 0.00)
Cohort total years working for pay* (x 1000)	1,627 (1,564 to 1,690)	1,628 (1,565 to 1,691)	1.2 (-0.1 to 2.5)	0.07 (-0.01 to 0.15)
Cohort total earnings (millions, $2024, disc.)*	46,968 (44,271 to 49,665)	46,982 (44,293 to 49,672)	15 (-40 to 69)	0.03 (-0.07 to 0.13)
Cohort total earnings (millions, $2024, disc.)	47,776 (45,006 to 50,546)	47,809 (45,033 to 50,584)	33 (-71 to 136)	0.07 (-0.14 to 0.28)
Cohort total medical costs (millions, $2024, disc.)*	564,893 (543,172 to 586,613)	559,680 (538,386 to 580,974)	-5,212 (-6,370 to -4,055)	-0.92 (-1.11 to -0.73)
Cohort total medical costs (millions, $2024, disc.)	629,357 (606,686 to 652,028)	631,460 (608,840 to 654,080)	2,103 (105 to 4,100)	0.34 (0.03 to 0.65)
Cohort total QALYs (millions, $2024, disc.)	1,705,870 (1,637,781 to 1,773,959)	1,725,793 (1,659,114 to 1,792,471)	19,923 (14,173 to 25,672)	1.17 (0.82 to 1.52)

* Includes only waves in which respondent is alive in both status quo and counterfactual scenarios

Table S7f: age 75-79 at the start of the simulation.

	Status quo: Using sleep medication at baseline	Counterfactual: No future sleep medication	Difference	Percentage difference
Sleep medication use (% at start)	100 (100 to 100)	100 (100 to 100)	0 (0 to 0)	(0 to 0)
Sleep medication use (avg lifetime %)	64.2 (63.4 to 65.0)	0.0 (0.0 to 0.0)	-64.2 (-65.0 to -63.4)	-100.0 (-100.0 to -100.0)
Injury due to fall (% ever)*	55.3 (53.3 to 57.3)	48.8 (47.0 to 50.5)	-6.53 (-7.54 to -5.52)	-11.77 (-13.44 to -10.10)
TICS cognitive score < 12 (% ever)*	80.4 (79.5 to 81.3)	78.3 (77.3 to 79.2)	-2.12 (-2.57 to -1.68)	-2.63 (-3.18 to -2.08)
Living in nursing home (% ever)*	29.1 (27.6 to 30.6)	28.3 (26.8 to 29.7)	-0.81 (-1.02 to -0.61)	-2.81 (-3.49 to -2.13)
Any limitations in ADLs (% ever)*	77.7 (76.7 to 78.6)	77.5 (76.5 to 78.4)	-0.20 (-0.28 to -0.13)	-0.26 (-0.36 to -0.17)
Any limitations in IADLs (% ever)*	77.0 (76.1 to 77.9)	76.5 (75.5 to 77.4)	-0.56 (-0.71 to -0.41)	-0.72 (-0.92 to -0.53)
Avg nr of survey waves with any injury due to fall*	1.14 (1.07 to 1.22)	0.95 (0.88 to 1.01)	-0.20 (-0.23 to -0.16)	-17.26 (-19.78 to -14.74)
Avg years with TICS cognitive score < 12*	5.15 (4.99 to 5.31)	4.83 (4.66 to 4.99)	-0.32 (-0.38 to -0.26)	-6.27 (-7.47 to -5.07)
Avg years in nursing home*	1.51 (1.41 to 1.62)	1.46 (1.36 to 1.56)	-0.05 (-0.07 to -0.04)	-3.47 (-4.35 to -2.59)
Avg years with limitations in any ADLs*	4.75 (4.61 to 4.89)	4.72 (4.58 to 4.86)	-0.03 (-0.03 to -0.02)	-0.53 (-0.71 to -0.35)
Avg years with limitations in any IADLs*	4.66 (4.53 to 4.80)	4.59 (4.47 to 4.72)	-0.07 (-0.09 to -0.05)	-1.49 (-1.82 to -1.15)
Avg years receiving disability benefits*	0.00 (0.00 to 0.00)	0.00 (0.00 to 0.00)	0.00 (0.00 to 0.00)	0.00 (0.00 to 0.00)
Avg years working for pay*	0.39 (0.37 to 0.41)	0.39 (0.37 to 0.41)	0.00 (-0.00 to 0.00)	0.05 (-0.06 to 0.16)
Avg earnings (x 1000, $2024, disc.)*	9 (9 to 10)	9 (9 to 10)	0.00 (-0.01 to 0.01)	0.03 (-0.05 to 0.10)
Avg earnings (x 1000, $2024, disc.)	8 (8 to 9)	8 (8 to 9)	0.01 (-0.02 to 0.03)	0.07 (-0.16 to 0.31)
Avg medical costs (x 1000, $2024, disc.)*	333 (319 to 347)	330 (316 to 343)	-3.09 (-3.85 to -2.33)	-0.93 (-1.14 to -0.71)
Avg medical costs (x 1000, $2024, disc.)	329 (316 to 342)	330 (317 to 344)	1.38 (0.32 to 2.45)	0.42 (0.10 to 0.74)
Avg age at death (years)	85.7 (85.5 to 85.8)	85.8 (85.6 to 86.0)	0.13 (0.09 to 0.16)	0.15 (0.11 to 0.19)
Avg life years	10.2 (10.1 to 10.4)	10.4 (10.2 to 10.5)	0.13 (0.09 to 0.16)	1.23 (0.91 to 1.56)
Avg QALYs	5.0 (4.6 to 5.4)	5.1 (4.7 to 5.4)	0.09 (0.06 to 0.12)	1.76 (1.15 to 2.36)
Avg QALYs (x 1000, 2024$, disc.)	660 (618 to 701)	669 (628 to 710)	9.8 (6.6 to 12.9)	1.48 (0.97 to 1.99)
Cohort total life years (x 1000)	11,709 (11,522 to 11,896)	11,853 (11,662 to 12,044)	144 (106 to 183)	1.23 (0.91 to 1.56)
Cohort total QALYs (x 1000)	5,724 (5,314 to 6,134)	5,825 (5,421 to 6,228)	100 (68 to 133)	1.76 (1.15 to 2.36)
Cohort total nursing home years* (x 1000)	1,478 (1,373 to 1,582)	1,426 (1,326 to 1,526)	-52 (-65 to -38)	-3.5 (-4.4 to -2.6)
Cohort total years claiming disability* (x 1000)	0 (0 to 0)	0 (0 to 0)	0.0 (0.0 to 0.0)	0.00 (0.00 to 0.00)
Cohort total years working for pay* (x 1000)	381 (363 to 399)	381 (364 to 399)	0.2 (-0.3 to 0.6)	0.05 (-0.06 to 0.16)
Cohort total earnings (millions, $2024, disc.)*	9,228 (8,476 to 9,980)	9,231 (8,479 to 9,982)	2 (-6 to 10)	0.03 (-0.05 to 0.10)
Cohort total earnings (millions, $2024, disc.)	9,553 (8,784 to 10,322)	9,560 (8,789 to 10,331)	7 (-18 to 32)	0.07 (-0.16 to 0.31)
Cohort total medical costs (millions, $2024, disc.)*	325,067 (311,544 to 338,590)	322,050 (308,756 to 335,344)	-3,017 (-3,760 to -2,274)	-0.93 (-1.14 to -0.71)
Cohort total medical costs (millions, $2024, disc.)	376,365 (361,343 to 391,387)	377,948 (362,995 to 392,902)	1,584 (364 to 2,803)	0.42 (0.10 to 0.74)
Cohort total QALYs (millions, $2024, disc.)	754,321 (706,797 to 801,845)	765,478 (718,720 to 812,237)	11,157 (7,549 to 14,765)	1.48 (0.97 to 1.99)

* Includes only waves in which respondent is alive in both status quo and counterfactual scenarios

Table S7g: age 80-84 at the start of the simulation.

	Status quo: Using sleep medication at baseline	Counterfactual: No future sleep medication	Difference	Percentage difference
Sleep medication use (% at start)	100 (100 to 100)	100 (100 to 100)	0 (0 to 0)	(0 to 0)
Sleep medication use (avg lifetime %)	70.1 (69.1 to 71.1)	0.0 (0.0 to 0.0)	-70.1 (-71.1 to -69.1)	-100.0 (-100.0 to -100.0)
Injury due to fall (% ever)*	52.3 (50.2 to 54.4)	45.5 (43.7 to 47.4)	-6.80 (-7.93 to -5.68)	-13.02 (-14.99 to -11.04)
TICS cognitive score < 12 (% ever)*	82.6 (81.5 to 83.6)	80.8 (79.9 to 81.8)	-1.74 (-2.22 to -1.25)	-2.10 (-2.68 to -1.53)
Living in nursing home (% ever)*	31.2 (29.9 to 32.5)	30.5 (29.2 to 31.8)	-0.67 (-0.92 to -0.43)	-2.17 (-2.96 to -1.37)
Any limitations in ADLs (% ever)*	78.3 (77.5 to 79.2)	78.2 (77.4 to 79.0)	-0.13 (-0.20 to -0.06)	-0.17 (-0.26 to -0.07)
Any limitations in IADLs (% ever)*	80.1 (79.1 to 81.1)	79.7 (78.7 to 80.7)	-0.43 (-0.57 to -0.30)	-0.54 (-0.71 to -0.37)
Avg nr of survey waves with any injury due to fall*	0.99 (0.92 to 1.06)	0.81 (0.75 to 0.86)	-0.18 (-0.21 to -0.14)	-17.91 (-20.58 to -15.23)
Avg years with TICS cognitive score < 12*	4.56 (4.43 to 4.70)	4.33 (4.18 to 4.47)	-0.24 (-0.29 to -0.18)	-5.17 (-6.31 to -4.04)
Avg years in nursing home*	1.48 (1.40 to 1.56)	1.44 (1.36 to 1.52)	-0.04 (-0.06 to -0.03)	-2.90 (-3.88 to -1.92)
Avg years with limitations in any ADLs*	4.10 (3.99 to 4.21)	4.08 (3.97 to 4.18)	-0.02 (-0.03 to -0.01)	-0.54 (-0.73 to -0.36)
Avg years with limitations in any IADLs*	4.23 (4.11 to 4.35)	4.19 (4.07 to 4.31)	-0.05 (-0.06 to -0.03)	-1.09 (-1.39 to -0.79)
Avg years receiving disability benefits*	0.00 (0.00 to 0.00)	0.00 (0.00 to 0.00)	0.00 (0.00 to 0.00)	0.00 (0.00 to 0.00)
Avg years working for pay*	0.12 (0.12 to 0.13)	0.12 (0.12 to 0.13)	0.00 (-0.00 to 0.00)	0.00 (-0.04 to 0.04)
Avg earnings (x 1000, $2024, disc.)*	5 (5 to 6)	5 (5 to 6)	0.00 (-0.00 to 0.00)	0.00 (-0.01 to 0.02)
Avg earnings (x 1000, $2024, disc.)	4 (4 to 5)	4 (4 to 5)	0.00 (-0.00 to 0.00)	0.00 (-0.09 to 0.10)
Avg medical costs (x 1000, $2024, disc.)*	299 (287 to 310)	296 (285 to 307)	-2.59 (-3.38 to -1.80)	-0.86 (-1.12 to -0.61)
Avg medical costs (x 1000, $2024, disc.)	294 (283 to 305)	296 (285 to 307)	1.57 (0.25 to 2.89)	0.54 (0.11 to 0.97)
Avg age at death (years)	88.1 (87.9 to 88.2)	88.1 (88.0 to 88.3)	0.09 (0.07 to 0.12)	0.11 (0.08 to 0.14)
Avg life years	7.8 (7.7 to 8.0)	7.9 (7.8 to 8.1)	0.09 (0.07 to 0.12)	1.19 (0.86 to 1.53)
Avg QALYs	3.4 (3.1 to 3.7)	3.4 (3.1 to 3.8)	0.06 (0.04 to 0.08)	1.75 (1.03 to 2.47)
Avg QALYs (x 1000, 2024$, disc.)	466 (425 to 507)	473 (432 to 513)	6.9 (4.3 to 9.4)	1.48 (0.87 to 2.08)
Cohort total life years (x 1000)	8,468 (8,311 to 8,624)	8,569 (8,405 to 8,732)	101 (72 to 129)	1.19 (0.86 to 1.53)
Cohort total QALYs (x 1000)	3,670 (3,311 to 4,029)	3,734 (3,379 to 4,089)	64 (40 to 88)	1.75 (1.03 to 2.47)
Cohort total nursing home years* (x 1000)	1,321 (1,250 to 1,392)	1,283 (1,213 to 1,353)	-39 (-52 to -25)	-2.9 (-3.9 to -1.9)
Cohort total years claiming disability* (x 1000)	0 (0 to 0)	0 (0 to 0)	0.0 (0.0 to 0.0)	0.00 (0.00 to 0.00)
Cohort total years working for pay* (x 1000)	109 (105 to 113)	109 (105 to 113)	0.0 (-0.0 to 0.0)	0.00 (-0.04 to 0.04)
Cohort total earnings (millions, $2024, disc.)*	4,639 (4,322 to 4,956)	4,639 (4,322 to 4,956)	0 (-1 to 1)	0.00 (-0.01 to 0.02)
Cohort total earnings (millions, $2024, disc.)	4,810 (4,490 to 5,129)	4,810 (4,490 to 5,130)	0 (-5 to 5)	0.00 (-0.09 to 0.10)
Cohort total medical costs (millions, $2024, disc.)*	266,521 (256,205 to 276,838)	264,209 (254,005 to 274,414)	-2,312 (-3,020 to -1,604)	-0.86 (-1.12 to -0.61)
Cohort total medical costs (millions, $2024, disc.)	318,626 (306,940 to 330,312)	320,324 (308,211 to 332,438)	1,698 (271 to 3,125)	0.54 (0.11 to 0.97)
Cohort total QALYs (millions, $2024, disc.)	504,476 (460,152 to 548,799)	511,914 (468,138 to 555,691)	7,439 (4,669 to 10,208)	1.48 (0.87 to 2.08)

* Includes only waves in which respondent is alive in both status quo and counterfactual scenarios

Table S7h: age 85+ at the start of the simulation.

	Status quo: Using sleep medication at baseline	Counterfactual: No future sleep medication	Difference	Percentage difference
Sleep medication use (% at start)	100 (100 to 100)	100 (100 to 100)	0 (0 to 0)	(0 to 0)
Sleep medication use (avg lifetime %)	80.2 (79.1 to 81.2)	0.0 (0.0 to 0.0)	-80.2 (-81.2 to -79.1)	-100.0 (-100.0 to -100.0)
Injury due to fall (% ever)*	50.1 (47.7 to 52.5)	42.6 (40.4 to 44.7)	-7.49 (-8.66 to -6.32)	-14.95 (-17.07 to -12.84)
TICS cognitive score < 12 (% ever)*	86.8 (85.9 to 87.6)	85.6 (84.8 to 86.4)	-1.16 (-1.48 to -0.85)	-1.34 (-1.69 to -0.98)
Living in nursing home (% ever)*	44.9 (43.7 to 46.1)	44.5 (43.4 to 45.7)	-0.38 (-0.54 to -0.23)	-0.84 (-1.19 to -0.50)
Any limitations in ADLs (% ever)*	82.3 (81.4 to 83.2)	82.2 (81.3 to 83.2)	-0.08 (-0.14 to -0.02)	-0.10 (-0.17 to -0.02)
Any limitations in IADLs (% ever)*	85.2 (84.4 to 86.0)	85.0 (84.2 to 85.9)	-0.17 (-0.25 to -0.09)	-0.19 (-0.29 to -0.10)
Avg nr of survey waves with any injury due to fall*	0.85 (0.79 to 0.91)	0.69 (0.64 to 0.74)	-0.16 (-0.19 to -0.13)	-18.86 (-21.47 to -16.26)
Avg years with TICS cognitive score < 12*	3.95 (3.84 to 4.07)	3.83 (3.72 to 3.94)	-0.12 (-0.15 to -0.10)	-3.15 (-3.84 to -2.45)
Avg years in nursing home*	1.91 (1.82 to 1.99)	1.88 (1.80 to 1.97)	-0.02 (-0.03 to -0.01)	-1.18 (-1.62 to -0.74)
Avg years with limitations in any ADLs*	3.53 (3.42 to 3.64)	3.52 (3.41 to 3.63)	-0.01 (-0.01 to -0.00)	-0.22 (-0.34 to -0.10)
Avg years with limitations in any IADLs*	3.80 (3.69 to 3.92)	3.78 (3.67 to 3.89)	-0.02 (-0.03 to -0.01)	-0.51 (-0.68 to -0.35)
Avg years receiving disability benefits*	0.00 (0.00 to 0.00)	0.00 (0.00 to 0.00)	0.00 (0.00 to 0.00)	0.00 (0.00 to 0.00)
Avg years working for pay*	0.08 (0.08 to 0.08)	0.08 (0.08 to 0.08)	0.00 (0.00 to 0.00)	0.00 (0.00 to 0.00)
Avg earnings (x 1000, $2024, disc.)*	3 (3 to 3)	3 (3 to 3)	-0.00 (-0.00 to 0.00)	-0.00 (-0.00 to 0.00)
Avg earnings (x 1000, $2024, disc.)	2 (2 to 3)	2 (2 to 3)	0.00 (-0.00 to 0.00)	0.00 (-0.14 to 0.14)
Avg medical costs (x 1000, $2024, disc.)*	274 (263 to 285)	273 (262 to 284)	-1.32 (-1.79 to -0.85)	-0.48 (-0.65 to -0.32)
Avg medical costs (x 1000, $2024, disc.)	259 (249 to 269)	260 (251 to 270)	1.30 (0.42 to 2.17)	0.50 (0.17 to 0.84)
Avg age at death (years)	93.2 (93.1 to 93.3)	93.2 (93.1 to 93.3)	0.05 (0.03 to 0.06)	0.05 (0.04 to 0.07)
Avg life years	5.2 (5.1 to 5.3)	5.2 (5.1 to 5.3)	0.05 (0.03 to 0.06)	0.91 (0.63 to 1.18)
Avg QALYs	1.7 (1.5 to 2.0)	1.8 (1.5 to 2.0)	0.03 (0.01 to 0.04)	1.55 (0.68 to 2.41)
Avg QALYs (x 1000, 2024$, disc.)	250 (212 to 288)	253 (216 to 290)	3.3 (1.8 to 4.7)	1.30 (0.58 to 2.02)
Cohort total life years (x 1000)	8,932 (8,758 to 9,106)	9,014 (8,830 to 9,197)	82 (57 to 106)	0.91 (0.63 to 1.18)
Cohort total QALYs (x 1000)	3,013 (2,524 to 3,502)	3,060 (2,579 to 3,541)	47 (25 to 68)	1.55 (0.68 to 2.41)
Cohort total nursing home years* (x 1000)	2,464 (2,352 to 2,576)	2,435 (2,327 to 2,543)	-29 (-40 to -18)	-1.2 (-1.6 to -0.7)
Cohort total years claiming disability* (x 1000)	0 (0 to 0)	0 (0 to 0)	0.0 (0.0 to 0.0)	0.00 (0.00 to 0.00)
Cohort total years working for pay* (x 1000)	97 (97 to 97)	97 (97 to 97)	0.0 (0.0 to 0.0)	0.00 (0.00 to 0.00)
Cohort total earnings (millions, $2024, disc.)*	3,799 (3,513 to 4,086)	3,799 (3,513 to 4,086)	0 (0 to 0)	0.00 (0.00 to 0.00)
Cohort total earnings (millions, $2024, disc.)	4,060 (3,761 to 4,360)	4,060 (3,760 to 4,361)	0 (-5 to 5)	0.00 (-0.14 to 0.14)
Cohort total medical costs (millions, $2024, disc.)*	353,972 (339,602 to 368,342)	352,270 (338,053 to 366,486)	-1,702 (-2,312 to -1,092)	-0.48 (-0.65 to -0.32)
Cohort total medical costs (millions, $2024, disc.)	447,667 (430,890 to 464,445)	449,908 (432,835 to 466,980)	2,240 (723 to 3,757)	0.50 (0.17 to 0.84)
Cohort total QALYs (millions, $2024, disc.)	432,049 (367,003 to 497,096)	437,666 (373,613 to 501,719)	5,617 (3,035 to 8,199)	1.30 (0.58 to 2.02)

* Includes only waves in which respondent is alive in both status quo and counterfactual scenarios

Table S7i: Lifetime effects of sleep medication elimination for **women** (age 51+).

	**Status quo: Using sleep medication at baseline**	**Counterfactual: No future sleep medication**	**Difference**	**Percentage difference**
Sleep medication use (% at start)	100 (100 to 100)	100 (100 to 100)	0 (0 to 0)	(0 to 0)
Sleep medication use (avg lifetime %)	52.2 (51.1 to 53.4)	0.0 (0.0 to 0.0)	-52.2 (-53.4 to -51.1)	-100.0 (-100.0 to -100.0)
Sleep disturbance (JSS of 3 or more, % at start)	76.3 (76.3 to 76.3)	76.3 (76.3 to 76.3)	0.00 (0.00 to 0.00)	0.00 (0.00 to 0.00)
Injury due to fall (% ever)*	70.7 (69.3 to 72.1)	65.5 (64.2 to 66.7)	-5.20 (-6.00 to -4.40)	-7.35 (-8.43 to -6.27)
TICS cognitive score < 12 (% ever)*	77.0 (75.9 to 78.0)	75.3 (74.1 to 76.4)	-1.70 (-2.06 to -1.35)	-2.21 (-2.68 to -1.75)
Living in nursing home (% ever)*	28.3 (26.5 to 30.1)	27.7 (25.9 to 29.4)	-0.63 (-0.79 to -0.48)	-2.24 (-2.79 to -1.69)
Any limitations in ADLs (% ever)*	83.5 (82.6 to 84.4)	83.3 (82.5 to 84.2)	-0.12 (-0.16 to -0.08)	-0.14 (-0.19 to -0.09)
Any limitations in IADLs (% ever)*	82.8 (81.9 to 83.7)	82.5 (81.6 to 83.3)	-0.33 (-0.41 to -0.25)	-0.40 (-0.49 to -0.31)
Avg nr of survey waves with any injury due to fall*	1.96 (1.86 to 2.06)	1.70 (1.61 to 1.78)	-0.26 (-0.31 to -0.22)	-13.45 (-15.47 to -11.43)
Avg years with TICS cognitive score < 12*	5.87 (5.66 to 6.07)	5.55 (5.34 to 5.76)	-0.32 (-0.37 to -0.26)	-5.42 (-6.40 to -4.44)
Avg years in nursing home*	1.47 (1.34 to 1.60)	1.42 (1.30 to 1.55)	-0.05 (-0.06 to -0.04)	-3.11 (-3.81 to -2.41)
Avg years with limitations in any ADLs*	6.53 (6.32 to 6.73)	6.49 (6.28 to 6.69)	-0.04 (-0.05 to -0.03)	-0.62 (-0.77 to -0.47)
Avg years with limitations in any IADLs*	5.99 (5.79 to 6.19)	5.92 (5.72 to 6.11)	-0.07 (-0.09 to -0.06)	-1.23 (-1.46 to -0.99)
Avg years receiving disability benefits*	1.16 (1.12 to 1.21)	1.16 (1.12 to 1.21)	-0.00 (-0.00 to -0.00)	-0.04 (-0.08 to -0.00)
Avg years working for pay*	2.93 (2.84 to 3.03)	2.93 (2.84 to 3.03)	0.00 (0.00 to 0.00)	0.06 (0.03 to 0.10)
Avg earnings (x 1000, $2024, disc.)*	137 (131 to 143)	137 (131 to 143)	0.04 (0.00 to 0.08)	0.03 (0.00 to 0.05)
Avg earnings (x 1000, $2024, disc.)	129 (123 to 134)	129 (123 to 134)	0.05 (0.00 to 0.10)	0.04 (0.00 to 0.08)
Avg medical costs (x 1000, $2024, disc.)*	377 (364 to 391)	375 (361 to 389)	-2.30 (-2.79 to -1.82)	-0.61 (-0.73 to -0.49)
Avg medical costs (x 1000, $2024, disc.)	387 (373 to 400)	388 (374 to 402)	1.42 (0.56 to 2.27)	0.37 (0.15 to 0.58)
Avg age at death (years)	85.3 (85.0 to 85.5)	85.4 (85.1 to 85.7)	0.13 (0.10 to 0.16)	0.16 (0.12 to 0.19)
Avg life years	19.1 (18.9 to 19.4)	19.3 (19.0 to 19.5)	0.13 (0.10 to 0.16)	0.69 (0.53 to 0.86)
Avg QALYs	11.0 (10.6 to 11.5)	11.1 (10.7 to 11.6)	0.10 (0.07 to 0.13)	0.88 (0.61 to 1.15)
Avg QALYs (x 1000, 2024$, disc.)	1,235 (1,191 to 1,279)	1,244 (1,200 to 1,288)	8.9 (6.2 to 11.6)	0.72 (0.50 to 0.94)
Cohort total life years (x 1000)	182,319 (179,907 to 184,731)	183,583 (181,110 to 186,055)	1,264 (957 to 1,570)	0.69 (0.53 to 0.86)
Cohort total QALYs (x 1000)	105,059 (100,779 to 109,339)	105,986 (101,750 to 110,223)	927 (653 to 1,201)	0.88 (0.61 to 1.15)
Cohort total nursing home years* (x 1000)	13,039 (11,894 to 14,184)	12,634 (11,503 to 13,765)	-405 (-496 to -313)	-3.1 (-3.8 to -2.4)
Cohort total years claiming disability* (x 1000)	10,328 (9,914 to 10,743)	10,324 (9,909 to 10,739)	-4.1 (-8.1 to -0.0)	-0.04 (-0.08 to -0.00)
Cohort total years working for pay* (x 1000)	26,038 (25,206 to 26,871)	26,055 (25,222 to 26,889)	16.8 (8.4 to 25.2)	0.06 (0.03 to 0.10)
Cohort total earnings (millions, $2024, disc.)*	1,218,883 (1,167,282 to 1,270,484)	1,219,221 (1,167,570 to 1,270,872)	338 (4 to 673)	0.03 (0.00 to 0.05)
Cohort total earnings (millions, $2024, disc.)	1,225,766 (1,174,059 to 1,277,474)	1,226,253 (1,174,521 to 1,277,984)	486 (30 to 942)	0.04 (0.00 to 0.08)
Cohort total medical costs (millions, $2024, disc.)*	3,351,253 (3,228,999 to 3,473,507)	3,330,810 (3,210,005 to 3,451,616)	-20,443 (-24,754 to -16,132)	-0.61 (-0.73 to -0.49)
Cohort total medical costs (millions, $2024, disc.)	3,686,623 (3,556,125 to 3,817,121)	3,700,124 (3,566,989 to 3,833,258)	13,501 (5,358 to 21,644)	0.37 (0.15 to 0.58)
Cohort total QALYs (millions, $2024, disc.)	11,771,318 (11,350,105 to 12,192,531)	11,855,987 (11,439,235 to 12,272,739)	84,668 (59,215 to 110,122)	0.72 (0.50 to 0.94)

* Includes only waves in which respondent is alive in both status quo and counterfactual scenarios

Table S7j: Lifetime effects of sleep medication elimination for **men** (age 51+).

	Status quo: Using sleep medication at baseline	Counterfactual: No future sleep medication	Difference	Percentage difference
Sleep medication use (% at start)	100 (100 to 100)	100 (100 to 100)	0 (0 to 0)	(0 to 0)
Sleep medication use (avg lifetime %)	50.9 (49.8 to 51.9)	0.0 (0.0 to 0.0)	-50.9 (-51.9 to -49.8)	-100.0 (-100.0 to -100.0)
Sleep disturbance (JSS of 3 or more, % at start)	74.8 (74.8 to 74.8)	74.8 (74.8 to 74.8)	0.00 (0.00 to 0.00)	0.00 (0.00 to 0.00)
Injury due to fall (% ever)*	44.0 (42.2 to 45.7)	38.9 (37.1 to 40.6)	-5.10 (-5.87 to -4.34)	-11.60 (-13.30 to -9.90)
TICS cognitive score < 12 (% ever)*	73.1 (71.8 to 74.5)	71.7 (70.2 to 73.1)	-1.46 (-1.78 to -1.15)	-2.00 (-2.44 to -1.57)
Living in nursing home (% ever)*	18.3 (16.9 to 19.7)	18.0 (16.6 to 19.3)	-0.35 (-0.44 to -0.25)	-1.89 (-2.37 to -1.41)
Any limitations in ADLs (% ever)*	77.8 (76.8 to 78.8)	77.7 (76.7 to 78.7)	-0.08 (-0.12 to -0.04)	-0.10 (-0.15 to -0.06)
Any limitations in IADLs (% ever)*	72.9 (71.4 to 74.4)	72.7 (71.2 to 74.1)	-0.23 (-0.31 to -0.16)	-0.32 (-0.42 to -0.22)
Avg nr of survey waves with any injury due to fall*	0.79 (0.74 to 0.84)	0.67 (0.62 to 0.71)	-0.12 (-0.14 to -0.10)	-15.54 (-17.69 to -13.39)
Avg years with TICS cognitive score < 12*	5.23 (5.04 to 5.42)	5.01 (4.81 to 5.22)	-0.22 (-0.26 to -0.18)	-4.21 (-5.01 to -3.40)
Avg years in nursing home*	0.80 (0.71 to 0.90)	0.78 (0.69 to 0.88)	-0.02 (-0.03 to -0.01)	-2.56 (-3.22 to -1.90)
Avg years with limitations in any ADLs*	4.95 (4.77 to 5.13)	4.93 (4.75 to 5.12)	-0.02 (-0.02 to -0.01)	-0.34 (-0.43 to -0.25)
Avg years with limitations in any IADLs*	4.34 (4.16 to 4.52)	4.30 (4.12 to 4.48)	-0.04 (-0.05 to -0.04)	-1.02 (-1.24 to -0.80)
Avg years receiving disability benefits*	1.44 (1.39 to 1.49)	1.44 (1.39 to 1.49)	-0.00 (-0.00 to 0.00)	-0.01 (-0.04 to 0.02)
Avg years working for pay*	3.43 (3.34 to 3.52)	3.43 (3.34 to 3.52)	0.00 (-0.00 to 0.00)	0.02 (-0.00 to 0.05)
Avg earnings (x 1000, $2024, disc.)*	232 (221 to 243)	232 (221 to 243)	0.02 (-0.02 to 0.06)	0.01 (-0.01 to 0.03)
Avg earnings (x 1000, $2024, disc.)	212 (202 to 222)	212 (202 to 222)	0.05 (-0.01 to 0.12)	0.03 (-0.00 to 0.06)
Avg medical costs (x 1000, $2024, disc.)*	331 (318 to 343)	329 (317 to 342)	-1.17 (-1.46 to -0.88)	-0.35 (-0.44 to -0.27)
Avg medical costs (x 1000, $2024, disc.)	335 (323 to 348)	336 (324 to 348)	1.00 (0.53 to 1.47)	0.30 (0.16 to 0.44)
Avg age at death (years)	81.2 (80.9 to 81.5)	81.3 (81.0 to 81.6)	0.08 (0.06 to 0.10)	0.10 (0.08 to 0.12)
Avg life years	16.3 (16.1 to 16.6)	16.4 (16.2 to 16.7)	0.08 (0.06 to 0.10)	0.50 (0.37 to 0.62)
Avg QALYs	9.8 (9.4 to 10.1)	9.8 (9.5 to 10.2)	0.06 (0.05 to 0.08)	0.64 (0.47 to 0.81)
Avg QALYs (x 1000, 2024$, disc.)	1,123 (1,086 to 1,160)	1,129 (1,092 to 1,166)	6.0 (4.4 to 7.5)	0.53 (0.38 to 0.68)
Cohort total life years (x 1000)	94,173 (92,567 to 95,779)	94,640 (93,039 to 96,242)	467 (353 to 581)	0.50 (0.37 to 0.62)
Cohort total QALYs (x 1000)	56,241 (54,175 to 58,307)	56,600 (54,565 to 58,635)	359 (268 to 450)	0.64 (0.47 to 0.81)
Cohort total nursing home years* (x 1000)	4,194 (3,684 to 4,704)	4,087 (3,596 to 4,577)	-107 (-142 to -73)	-2.6 (-3.2 to -1.9)
Cohort total years claiming disability* (x 1000)	7,522 (7,254 to 7,791)	7,521 (7,253 to 7,790)	-1.0 (-3.4 to 1.4)	-0.01 (-0.04 to 0.02)
Cohort total years working for pay* (x 1000)	17,894 (17,432 to 18,356)	17,898 (17,436 to 18,361)	4.1 (-0.4 to 8.7)	0.02 (-0.00 to 0.05)
Cohort total earnings (millions, $2024, disc.)*	1,208,464 (1,151,470 to 1,265,457)	1,208,581 (1,151,587 to 1,265,575)	117 (-80 to 314)	0.01 (-0.01 to 0.03)
Cohort total earnings (millions, $2024, disc.)	1,219,353 (1,161,761 to 1,276,946)	1,219,660 (1,162,115 to 1,277,205)	307 (-61 to 675)	0.03 (-0.00 to 0.06)
Cohort total medical costs (millions, $2024, disc.)*	1,723,286 (1,655,904 to 1,790,668)	1,717,184 (1,650,191 to 1,784,178)	-6,102 (-7,607 to -4,598)	-0.35 (-0.44 to -0.27)
Cohort total medical costs (millions, $2024, disc.)	1,931,317 (1,860,708 to 2,001,926)	1,937,087 (1,866,870 to 2,007,303)	5,770 (3,042 to 8,497)	0.30 (0.16 to 0.44)
Cohort total QALYs (millions, $2024, disc.)	6,469,717 (6,256,141 to 6,683,293)	6,504,118 (6,293,730 to 6,714,507)	34,401 (25,403 to 43,399)	0.53 (0.38 to 0.68)

* Includes only waves in which respondent is alive in both status quo and counterfactual scenarios

Table S7k: Lifetime effects of sleep medication elimination for the **Non-Hispanic Black** population (age 51+).

	Status quo: Using sleep medication at baseline	Counterfactual: No future sleep medication	Difference	Percentage difference
Sleep medication use (% at start)	100 (100 to 100)	100 (100 to 100)	0 (0 to 0)	(0 to 0)
Sleep medication use (avg lifetime %)	52.2 (50.7 to 53.6)	0.0 (0.0 to 0.0)	-52.2 (-53.6 to -50.7)	-100.0 (-100.0 to -100.0)
Sleep disturbance (JSS of 3 or more, % at start)	86.0 (86.0 to 86.0)	86.0 (86.0 to 86.0)	0.00 (0.00 to 0.00)	0.00 (0.00 to 0.00)
Injury due to fall (% ever)*	44.2 (41.7 to 46.7)	39.5 (37.0 to 42.0)	-4.72 (-5.43 to -4.01)	-10.67 (-12.29 to -9.06)
TICS cognitive score < 12 (% ever)*	84.5 (83.6 to 85.5)	83.7 (82.7 to 84.7)	-0.82 (-1.03 to -0.60)	-0.96 (-1.22 to -0.71)
Living in nursing home (% ever)*	20.6 (17.7 to 23.6)	20.2 (17.3 to 23.1)	-0.42 (-0.56 to -0.28)	-2.01 (-2.67 to -1.36)
Any limitations in ADLs (% ever)*	87.3 (86.3 to 88.3)	87.3 (86.2 to 88.3)	-0.08 (-0.12 to -0.04)	-0.09 (-0.14 to -0.04)
Any limitations in IADLs (% ever)*	84.4 (83.5 to 85.2)	84.1 (83.2 to 85.0)	-0.25 (-0.33 to -0.18)	-0.30 (-0.39 to -0.22)
Avg nr of survey waves with any injury due to fall*	0.87 (0.80 to 0.95)	0.74 (0.67 to 0.81)	-0.13 (-0.15 to -0.11)	-15.01 (-17.14 to -12.88)
Avg years with TICS cognitive score < 12*	8.13 (7.81 to 8.44)	7.88 (7.56 to 8.20)	-0.25 (-0.29 to -0.20)	-3.04 (-3.63 to -2.46)
Avg years in nursing home*	0.97 (0.78 to 1.16)	0.94 (0.76 to 1.13)	-0.03 (-0.04 to -0.02)	-3.22 (-4.04 to -2.40)
Avg years with limitations in any ADLs*	7.11 (6.81 to 7.41)	7.08 (6.78 to 7.39)	-0.03 (-0.04 to -0.02)	-0.41 (-0.52 to -0.30)
Avg years with limitations in any IADLs*	6.34 (6.07 to 6.61)	6.27 (6.01 to 6.53)	-0.07 (-0.08 to -0.05)	-1.05 (-1.23 to -0.88)
Avg years receiving disability benefits*	2.34 (2.26 to 2.42)	2.34 (2.26 to 2.42)	-0.00 (-0.00 to -0.00)	-0.03 (-0.04 to -0.01)
Avg years working for pay*	1.96 (1.86 to 2.07)	1.97 (1.86 to 2.08)	0.00 (-0.00 to 0.00)	0.06 (-0.01 to 0.13)
Avg earnings (x 1000, $2024, disc.)*	68 (64 to 72)	68 (64 to 72)	0.02 (-0.01 to 0.04)	0.02 (-0.02 to 0.06)
Avg earnings (x 1000, $2024, disc.)	64 (60 to 67)	64 (60 to 67)	0.03 (-0.02 to 0.08)	0.05 (-0.03 to 0.13)
Avg medical costs (x 1000, $2024, disc.)*	382 (357 to 406)	380 (356 to 404)	-1.69 (-2.14 to -1.25)	-0.44 (-0.55 to -0.34)
Avg medical costs (x 1000, $2024, disc.)	392 (368 to 416)	393 (369 to 417)	1.42 (0.66 to 2.18)	0.36 (0.18 to 0.55)
Avg age at death (years)	79.5 (79.2 to 79.9)	79.7 (79.3 to 80.1)	0.11 (0.08 to 0.14)	0.14 (0.11 to 0.17)
Avg life years	16.7 (16.3 to 17.1)	16.8 (16.4 to 17.2)	0.11 (0.08 to 0.14)	0.66 (0.51 to 0.82)
Avg QALYs	8.5 (7.9 to 9.0)	8.5 (8.0 to 9.1)	0.08 (0.06 to 0.10)	0.93 (0.64 to 1.21)
Avg QALYs (x 1000, 2024$, disc.)	974 (916 to 1,032)	981 (924 to 1,039)	7.4 (5.2 to 9.6)	0.76 (0.52 to 1.01)
Cohort total life years (x 1000)	27,581 (26,937 to 28,226)	27,764 (27,119 to 28,409)	183 (139 to 226)	0.66 (0.51 to 0.82)
Cohort total QALYs (x 1000)	13,952 (13,037 to 14,867)	14,081 (13,179 to 14,984)	129 (93 to 165)	0.93 (0.64 to 1.21)
Cohort total nursing home years* (x 1000)	1,483 (1,194 to 1,772)	1,435 (1,153 to 1,717)	-48 (-62 to -34)	-3.2 (-4.0 to -2.4)
Cohort total years claiming disability* (x 1000)	3,569 (3,444 to 3,695)	3,568 (3,443 to 3,694)	-0.9 (-1.5 to -0.3)	-0.03 (-0.04 to -0.01)
Cohort total years working for pay* (x 1000)	2,996 (2,830 to 3,163)	2,998 (2,831 to 3,165)	2.0 (-0.1 to 4.0)	0.06 (-0.01 to 0.13)
Cohort total earnings (millions, $2024, disc.)*	103,992 (97,860 to 110,125)	104,015 (97,883 to 110,148)	23 (-21 to 67)	0.02 (-0.02 to 0.06)
Cohort total earnings (millions, $2024, disc.)	104,955 (98,757 to 111,153)	105,009 (98,809 to 111,209)	53 (-27 to 134)	0.05 (-0.03 to 0.13)
Cohort total medical costs (millions, $2024, disc.)*	582,080 (545,088 to 619,071)	579,497 (542,752 to 616,243)	-2,583 (-3,259 to -1,906)	-0.44 (-0.55 to -0.34)
Cohort total medical costs (millions, $2024, disc.)	646,956 (607,580 to 686,333)	649,300 (609,551 to 689,050)	2,344 (1,093 to 3,596)	0.36 (0.18 to 0.55)
Cohort total QALYs (millions, $2024, disc.)	1,607,721 (1,512,272 to 1,703,171)	1,619,949 (1,525,725 to 1,714,173)	12,228 (8,611 to 15,845)	0.76 (0.52 to 1.01)

* Includes only waves in which respondent is alive in both status quo and counterfactual scenarios

Table S7l: Lifetime effects of sleep medication elimination for the **Hispanic** population (age 51+).

	Status quo: Using sleep medication at baseline	Counterfactual: No future sleep medication	Difference	Percentage difference
Sleep medication use (% at start)	100 (100 to 100)	100 (100 to 100)	0 (0 to 0)	(0 to 0)
Sleep medication use (avg lifetime %)	53.4 (51.3 to 55.4)	0.0 (0.0 to 0.0)	-53.4 (-55.4 to -51.3)	-100.0 (-100.0 to -100.0)
Sleep disturbance (JSS of 3 or more, % at start)	88.3 (88.3 to 88.3)	88.3 (88.3 to 88.3)	0.00 (0.00 to 0.00)	0.00 (0.00 to 0.00)
Injury due to fall (% ever)*	58.1 (55.3 to 60.8)	52.5 (49.7 to 55.2)	-5.58 (-6.61 to -4.54)	-9.59 (-11.34 to -7.85)
TICS cognitive score < 12 (% ever)*	87.6 (86.2 to 88.9)	86.6 (85.1 to 88.0)	-1.00 (-1.22 to -0.78)	-1.14 (-1.40 to -0.88)
Living in nursing home (% ever)*	17.6 (14.4 to 20.9)	17.1 (13.9 to 20.3)	-0.52 (-0.68 to -0.35)	-2.91 (-3.83 to -1.99)
Any limitations in ADLs (% ever)*	92.7 (91.5 to 93.9)	92.7 (91.5 to 93.8)	-0.04 (-0.08 to 0.01)	-0.04 (-0.09 to 0.01)
Any limitations in IADLs (% ever)*	90.3 (88.9 to 91.7)	90.1 (88.7 to 91.5)	-0.21 (-0.31 to -0.11)	-0.23 (-0.35 to -0.12)
Avg nr of survey waves with any injury due to fall*	1.33 (1.19 to 1.47)	1.12 (1.00 to 1.25)	-0.21 (-0.25 to -0.17)	-15.69 (-18.24 to -13.14)
Avg years with TICS cognitive score < 12*	9.14 (8.64 to 9.65)	8.79 (8.29 to 9.29)	-0.35 (-0.42 to -0.28)	-3.82 (-4.57 to -3.07)
Avg years in nursing home*	0.82 (0.60 to 1.04)	0.79 (0.58 to 1.00)	-0.03 (-0.04 to -0.02)	-3.51 (-4.61 to -2.42)
Avg years with limitations in any ADLs*	8.97 (8.22 to 9.71)	8.93 (8.19 to 9.67)	-0.04 (-0.05 to -0.02)	-0.40 (-0.53 to -0.27)
Avg years with limitations in any IADLs*	7.81 (7.18 to 8.45)	7.73 (7.09 to 8.36)	-0.09 (-0.11 to -0.06)	-1.10 (-1.39 to -0.81)
Avg years receiving disability benefits*	1.18 (1.13 to 1.23)	1.18 (1.13 to 1.23)	-0.00 (-0.00 to 0.00)	-0.06 (-0.13 to 0.01)
Avg years working for pay*	2.10 (1.99 to 2.22)	2.11 (1.99 to 2.22)	0.00 (0.00 to 0.00)	0.14 (0.06 to 0.22)
Avg earnings (x 1000, $2024, disc.)*	70 (66 to 74)	70 (66 to 74)	0.01 (-0.03 to 0.06)	0.02 (-0.05 to 0.09)
Avg earnings (x 1000, $2024, disc.)	66 (62 to 70)	66 (62 to 70)	0.02 (-0.05 to 0.09)	0.03 (-0.07 to 0.13)
Avg medical costs (x 1000, $2024, disc.)*	362 (332 to 393)	360 (330 to 391)	-1.82 (-2.37 to -1.27)	-0.50 (-0.63 to -0.37)
Avg medical costs (x 1000, $2024, disc.)	374 (344 to 403)	375 (346 to 405)	1.73 (0.74 to 2.72)	0.46 (0.21 to 0.71)
Avg age at death (years)	83.2 (82.6 to 83.8)	83.4 (82.8 to 84.0)	0.14 (0.10 to 0.18)	0.17 (0.12 to 0.22)
Avg life years	18.1 (17.5 to 18.7)	18.3 (17.7 to 18.8)	0.14 (0.10 to 0.18)	0.78 (0.55 to 1.00)
Avg QALYs	8.6 (8.0 to 9.2)	8.7 (8.1 to 9.3)	0.10 (0.06 to 0.13)	1.12 (0.69 to 1.56)
Avg QALYs (x 1000, 2024$, disc.)	985 (924 to 1,047)	995 (934 to 1,055)	9.2 (5.9 to 12.4)	0.93 (0.57 to 1.29)
Cohort total life years (x 1000)	26,983 (26,108 to 27,858)	27,193 (26,317 to 28,069)	210 (150 to 271)	0.78 (0.55 to 1.00)
Cohort total QALYs (x 1000)	12,849 (11,933 to 13,765)	12,994 (12,092 to 13,895)	144 (93 to 196)	1.12 (0.69 to 1.56)
Cohort total nursing home years* (x 1000)	1,141 (841 to 1,441)	1,100 (810 to 1,389)	-41 (-58 to -25)	-3.5 (-4.6 to -2.4)
Cohort total years claiming disability* (x 1000)	1,645 (1,575 to 1,716)	1,644 (1,574 to 1,715)	-0.9 (-2.0 to 0.2)	-0.06 (-0.13 to 0.01)
Cohort total years working for pay* (x 1000)	2,927 (2,765 to 3,089)	2,931 (2,768 to 3,093)	3.8 (1.5 to 6.0)	0.14 (0.06 to 0.22)
Cohort total earnings (millions, $2024, disc.)*	97,769 (91,953 to 103,586)	97,788 (91,989 to 103,588)	19 (-43 to 81)	0.02 (-0.05 to 0.09)
Cohort total earnings (millions, $2024, disc.)	98,477 (92,587 to 104,366)	98,508 (92,612 to 104,404)	31 (-73 to 134)	0.03 (-0.07 to 0.13)
Cohort total medical costs (millions, $2024, disc.)*	503,907 (461,500 to 546,314)	501,372 (459,294 to 543,451)	-2,534 (-3,297 to -1,771)	-0.50 (-0.63 to -0.37)
Cohort total medical costs (millions, $2024, disc.)	556,525 (512,208 to 600,842)	559,096 (514,626 to 603,566)	2,571 (1,098 to 4,043)	0.46 (0.21 to 0.71)
Cohort total QALYs (millions, $2024, disc.)	1,467,354 (1,375,518 to 1,559,189)	1,481,009 (1,390,726 to 1,571,292)	13,655 (8,788 to 18,523)	0.93 (0.57 to 1.29)

* Includes only waves in which respondent is alive in both status quo and counterfactual scenarios

Table S7m: Lifetime effects of sleep medication elimination for the **Non-Hispanic White** population (age 51+).

	**Status quo:** **Using sleep medication at baseline**	**Counterfactual:** **No future sleep medication**	**Difference**	**Percentage difference**
Sleep medication use (% at start)	100 (100 to 100)	100 (100 to 100)	0 (0 to 0)	(0 to 0)
Sleep medication use (avg lifetime %)	51.5 (50.5 to 52.4)	0.0 (0.0 to 0.0)	-51.5 (-52.4 to -50.5)	-100.0 (-100.0 to -100.0)
Sleep disturbance (JSS of 3 or more, % at start)	72.8 (72.8 to 72.8)	72.8 (72.8 to 72.8)	0.00 (0.00 to 0.00)	0.00 (0.00 to 0.00)
Injury due to fall (% ever)*	63.4 (62.1 to 64.7)	58.2 (57.1 to 59.4)	-5.17 (-5.95 to -4.40)	-8.16 (-9.31 to -7.01)
TICS cognitive score < 12 (% ever)*	72.8 (72.0 to 73.6)	71.0 (70.1 to 72.0)	-1.80 (-2.18 to -1.42)	-2.47 (-3.00 to -1.95)
Living in nursing home (% ever)*	26.0 (24.7 to 27.3)	25.5 (24.2 to 26.7)	-0.54 (-0.68 to -0.41)	-2.09 (-2.59 to -1.59)
Any limitations in ADLs (% ever)*	79.1 (78.4 to 79.9)	79.0 (78.3 to 79.7)	-0.12 (-0.16 to -0.08)	-0.15 (-0.20 to -0.10)
Any limitations in IADLs (% ever)*	77.0 (76.3 to 77.8)	76.7 (76.0 to 77.5)	-0.31 (-0.39 to -0.23)	-0.40 (-0.51 to -0.30)
Avg nr of survey waves with any injury due to fall*	1.64 (1.56 to 1.71)	1.42 (1.35 to 1.48)	-0.22 (-0.26 to -0.19)	-13.58 (-15.56 to -11.60)
Avg years with TICS cognitive score < 12*	4.85 (4.73 to 4.97)	4.58 (4.44 to 4.72)	-0.28 (-0.33 to -0.23)	-5.73 (-6.79 to -4.67)
Avg years in nursing home*	1.31 (1.21 to 1.40)	1.27 (1.18 to 1.36)	-0.04 (-0.05 to -0.03)	-2.90 (-3.56 to -2.24)
Avg years with limitations in any ADLs*	5.41 (5.27 to 5.54)	5.38 (5.24 to 5.51)	-0.03 (-0.04 to -0.02)	-0.58 (-0.73 to -0.44)
Avg years with limitations in any IADLs*	4.95 (4.83 to 5.07)	4.89 (4.78 to 5.00)	-0.06 (-0.07 to -0.05)	-1.20 (-1.43 to -0.97)
Avg years receiving disability benefits*	1.13 (1.09 to 1.17)	1.13 (1.09 to 1.17)	-0.00 (-0.00 to 0.00)	-0.03 (-0.06 to 0.01)
Avg years working for pay*	3.40 (3.31 to 3.49)	3.40 (3.32 to 3.49)	0.00 (0.00 to 0.00)	0.04 (0.01 to 0.07)
Avg earnings (x 1000, $2024, disc.)*	199 (191 to 208)	199 (191 to 208)	0.04 (-0.00 to 0.08)	0.02 (0.00 to 0.04)
Avg earnings (x 1000, $2024, disc.)	184 (177 to 192)	185 (177 to 192)	0.06 (0.01 to 0.11)	0.03 (0.00 to 0.06)
Avg medical costs (x 1000, $2024, disc.)*	357 (347 to 367)	355 (345 to 365)	-1.92 (-2.33 to -1.51)	-0.54 (-0.65 to -0.43)
Avg medical costs (x 1000, $2024, disc.)	363 (353 to 373)	364 (354 to 375)	1.18 (0.47 to 1.89)	0.33 (0.13 to 0.52)
Avg age at death (years)	84.4 (84.2 to 84.5)	84.5 (84.3 to 84.6)	0.11 (0.08 to 0.14)	0.13 (0.10 to 0.16)
Avg life years	18.3 (18.1 to 18.4)	18.4 (18.2 to 18.5)	0.11 (0.08 to 0.14)	0.60 (0.46 to 0.75)
Avg QALYs	11.1 (10.7 to 11.4)	11.2 (10.8 to 11.5)	0.08 (0.06 to 0.11)	0.75 (0.54 to 0.97)
Avg QALYs (x 1000, 2024$, disc.)	1,248 (1,211 to 1,285)	1,256 (1,219 to 1,292)	7.7 (5.5 to 9.8)	0.61 (0.43 to 0.79)
Cohort total life years (x 1000)	221,928 (220,040 to 223,817)	223,266 (221,353 to 225,180)	1,338 (1,014 to 1,661)	0.60 (0.46 to 0.75)
Cohort total QALYs (x 1000)	134,498 (129,956 to 139,040)	135,511 (131,023 to 139,999)	1,013 (731 to 1,295)	0.75 (0.54 to 0.97)
Cohort total nursing home years* (x 1000)	14,609 (13,534 to 15,684)	14,186 (13,139 to 15,232)	-423 (-526 to -321)	-2.9 (-3.6 to -2.2)
Cohort total years claiming disability* (x 1000)	12,636 (12,150 to 13,122)	12,633 (12,146 to 13,119)	-3.2 (-7.9 to 1.4)	-0.03 (-0.06 to 0.01)
Cohort total years working for pay* (x 1000)	38,009 (37,054 to 38,965)	38,024 (37,067 to 38,982)	15.2 (5.4 to 25.0)	0.04 (0.01 to 0.07)
Cohort total earnings (millions, $2024, disc.)*	2,225,585 (2,131,340 to 2,319,830)	2,225,998 (2,131,706 to 2,320,290)	413 (-16 to 843)	0.02 (0.00 to 0.04)
Cohort total earnings (millions, $2024, disc.)	2,241,688 (2,146,669 to 2,336,706)	2,242,396 (2,147,451 to 2,337,341)	709 (79 to 1,339)	0.03 (0.00 to 0.06)
Cohort total medical costs (millions, $2024, disc.)*	3,988,553 (3,875,172 to 4,101,934)	3,967,125 (3,854,859 to 4,079,390)	-21,428 (-26,017 to -16,839)	-0.54 (-0.65 to -0.43)
Cohort total medical costs (millions, $2024, disc.)	4,414,459 (4,291,224 to 4,537,694)	4,428,814 (4,303,950 to 4,553,679)	14,355 (5,715 to 22,996)	0.33 (0.13 to 0.52)
Cohort total QALYs (millions, $2024, disc.)	15,165,962 (14,712,592 to 15,619,332)	15,259,147 (14,811,236 to 15,707,058)	93,187 (66,712 to 119,661)	0.61 (0.43 to 0.79)

* Includes only waves in which respondent is alive in both status quo and counterfactual scenarios

Table S7n: Lifetime effects of sleep medication elimination for those with **less than high school** education (age 51+).

	Status quo: Using sleep medication at baseline	Counterfactual: No future sleep medication	Difference	Percentage difference
Sleep medication use (% at start)	100 (100 to 100)	100 (100 to 100)	0 (0 to 0)	(0 to 0)
Sleep medication use (avg lifetime %)	64.2 (62.8 to 65.7)	0.0 (0.0 to 0.0)	-64.2 (-65.7 to -62.8)	-100.0 (-100.0 to -100.0)
Sleep disturbance (JSS of 3 or more, % at start)	83.1 (83.1 to 83.1)	83.1 (83.1 to 83.1)	0.00 (0.00 to 0.00)	0.00 (0.00 to 0.00)
Injury due to fall (% ever)*	53.0 (50.8 to 55.2)	46.7 (44.7 to 48.7)	-6.32 (-7.35 to -5.29)	-11.93 (-13.71 to -10.16)
TICS cognitive score < 12 (% ever)*	90.7 (90.2 to 91.3)	89.8 (89.2 to 90.4)	-0.96 (-1.13 to -0.78)	-1.05 (-1.25 to -0.86)
Living in nursing home (% ever)*	24.1 (22.1 to 26.1)	23.5 (21.6 to 25.4)	-0.56 (-0.71 to -0.41)	-2.32 (-2.90 to -1.74)
Any limitations in ADLs (% ever)*	87.4 (86.7 to 88.2)	87.3 (86.5 to 88.1)	-0.10 (-0.14 to -0.05)	-0.11 (-0.16 to -0.06)
Any limitations in IADLs (% ever)*	87.3 (86.3 to 88.3)	87.0 (86.0 to 88.1)	-0.28 (-0.37 to -0.20)	-0.33 (-0.43 to -0.23)
Avg nr of survey waves with any injury due to fall*	1.14 (1.04 to 1.23)	0.93 (0.86 to 1.01)	-0.20 (-0.24 to -0.17)	-17.81 (-20.30 to -15.31)
Avg years with TICS cognitive score < 12*	8.16 (7.92 to 8.39)	7.87 (7.63 to 8.12)	-0.28 (-0.34 to -0.23)	-3.45 (-4.13 to -2.78)
Avg years in nursing home*	1.11 (0.97 to 1.24)	1.07 (0.94 to 1.20)	-0.04 (-0.05 to -0.03)	-3.35 (-4.15 to -2.54)
Avg years with limitations in any ADLs*	6.71 (6.41 to 7.02)	6.68 (6.38 to 6.98)	-0.03 (-0.04 to -0.02)	-0.45 (-0.60 to -0.30)
Avg years with limitations in any IADLs*	6.33 (6.03 to 6.62)	6.25 (5.96 to 6.54)	-0.08 (-0.10 to -0.06)	-1.22 (-1.52 to -0.92)
Avg years receiving disability benefits*	1.23 (1.19 to 1.28)	1.23 (1.19 to 1.28)	-0.00 (-0.00 to 0.00)	-0.01 (-0.05 to 0.04)
Avg years working for pay*	0.92 (0.85 to 0.98)	0.92 (0.85 to 0.98)	0.00 (-0.00 to 0.00)	0.10 (-0.03 to 0.22)
Avg earnings (x 1000, $2024, disc.)*	22 (20 to 24)	22 (20 to 24)	0.01 (-0.01 to 0.03)	0.05 (-0.05 to 0.14)
Avg earnings (x 1000, $2024, disc.)	20 (18 to 21)	20 (18 to 21)	0.01 (-0.01 to 0.04)	0.08 (-0.03 to 0.19)
Avg medical costs (x 1000, $2024, disc.)*	322 (305 to 339)	320 (303 to 337)	-2.21 (-2.72 to -1.69)	-0.68 (-0.83 to -0.54)
Avg medical costs (x 1000, $2024, disc.)	331 (315 to 347)	333 (317 to 348)	1.56 (0.64 to 2.49)	0.48 (0.20 to 0.75)
Avg age at death (years)	82.2 (81.9 to 82.5)	82.4 (82.1 to 82.6)	0.13 (0.09 to 0.16)	0.15 (0.11 to 0.20)
Avg life years	12.9 (12.6 to 13.2)	13.0 (12.7 to 13.3)	0.13 (0.09 to 0.16)	0.99 (0.72 to 1.25)
Avg QALYs	5.6 (5.1 to 6.0)	5.6 (5.2 to 6.1)	0.08 (0.05 to 0.11)	1.49 (0.89 to 2.09)
Avg QALYs (x 1000, 2024$, disc.)	673 (626 to 721)	682 (636 to 728)	8.4 (5.3 to 11.5)	1.24 (0.74 to 1.75)
Cohort total life years (x 1000)	36,803 (35,995 to 37,611)	37,166 (36,366 to 37,967)	363 (266 to 460)	0.99 (0.72 to 1.25)
Cohort total QALYs (x 1000)	15,885 (14,638 to 17,132)	16,123 (14,912 to 17,333)	238 (152 to 323)	1.49 (0.89 to 2.09)
Cohort total nursing home years* (x 1000)	2,829 (2,481 to 3,177)	2,734 (2,397 to 3,071)	-95 (-122 to -69)	-3.3 (-4.2 to -2.5)
Cohort total years claiming disability* (x 1000)	3,154 (3,042 to 3,265)	3,154 (3,042 to 3,265)	-0.2 (-1.7 to 1.3)	-0.01 (-0.05 to 0.04)
Cohort total years working for pay* (x 1000)	2,343 (2,175 to 2,512)	2,346 (2,177 to 2,515)	2.6 (-0.4 to 5.6)	0.10 (-0.03 to 0.22)
Cohort total earnings (millions, $2024, disc.)*	55,817 (51,415 to 60,219)	55,840 (51,428 to 60,253)	23 (-27 to 74)	0.05 (-0.05 to 0.14)
Cohort total earnings (millions, $2024, disc.)	56,517 (52,034 to 60,999)	56,557 (52,064 to 61,051)	40 (-21 to 102)	0.08 (-0.03 to 0.19)
Cohort total medical costs (millions, $2024, disc.)*	824,465 (781,150 to 867,779)	818,820 (775,921 to 861,718)	-5,645 (-6,969 to -4,322)	-0.68 (-0.83 to -0.54)
Cohort total medical costs (millions, $2024, disc.)	944,902 (899,431 to 990,374)	949,370 (904,015 to 994,725)	4,468 (1,837 to 7,098)	0.48 (0.20 to 0.75)
Cohort total QALYs (millions, $2024, disc.)	1,922,597 (1,786,836 to 2,058,358)	1,946,537 (1,814,777 to 2,078,297)	23,940 (15,098 to 32,782)	1.24 (0.74 to 1.75)

* Includes only waves in which respondent is alive in both status quo and counterfactual scenarios

Table S7o: Lifetime effects of sleep medication elimination for **high school graduates** (age 51+).

	Status quo: Using sleep medication at baseline	Counterfactual: No future sleep medication	Difference	Percentage difference
Sleep medication use (% at start)	100 (100 to 100)	100 (100 to 100)	0 (0 to 0)	(0 to 0)
Sleep medication use (avg lifetime %)	54.1 (53.0 to 55.1)	0.0 (0.0 to 0.0)	-54.1 (-55.1 to -53.0)	-100.0 (-100.0 to -100.0)
Sleep disturbance (JSS of 3 or more, % at start)	74.6 (74.6 to 74.6)	74.6 (74.6 to 74.6)	0.00 (0.00 to 0.00)	0.00 (0.00 to 0.00)
Injury due to fall (% ever)*	57.1 (55.6 to 58.6)	51.6 (50.1 to 53.0)	-5.51 (-6.39 to -4.62)	-9.64 (-11.12 to -8.16)
TICS cognitive score < 12 (% ever)*	75.4 (74.3 to 76.4)	73.9 (72.7 to 75.1)	-1.49 (-1.84 to -1.14)	-1.98 (-2.45 to -1.50)
Living in nursing home (% ever)*	24.1 (22.8 to 25.4)	23.6 (22.4 to 24.9)	-0.50 (-0.64 to -0.35)	-2.03 (-2.59 to -1.47)
Any limitations in ADLs (% ever)*	81.4 (80.5 to 82.2)	81.2 (80.4 to 82.1)	-0.11 (-0.16 to -0.06)	-0.13 (-0.19 to -0.07)
Any limitations in IADLs (% ever)*	78.0 (77.0 to 79.0)	77.6 (76.6 to 78.7)	-0.36 (-0.45 to -0.26)	-0.46 (-0.58 to -0.33)
Avg nr of survey waves with any injury due to fall*	1.31 (1.24 to 1.37)	1.11 (1.06 to 1.17)	-0.19 (-0.23 to -0.16)	-14.73 (-17.02 to -12.43)
Avg years with TICS cognitive score < 12*	5.56 (5.36 to 5.75)	5.27 (5.05 to 5.50)	-0.28 (-0.33 to -0.24)	-5.09 (-6.04 to -4.14)
Avg years in nursing home*	1.19 (1.10 to 1.28)	1.15 (1.07 to 1.24)	-0.03 (-0.04 to -0.03)	-2.89 (-3.55 to -2.22)
Avg years with limitations in any ADLs*	5.82 (5.63 to 6.00)	5.79 (5.60 to 5.97)	-0.03 (-0.04 to -0.02)	-0.54 (-0.69 to -0.40)
Avg years with limitations in any IADLs*	5.15 (5.00 to 5.31)	5.09 (4.93 to 5.25)	-0.06 (-0.08 to -0.05)	-1.23 (-1.46 to -1.00)
Avg years receiving disability benefits*	1.43 (1.37 to 1.49)	1.43 (1.37 to 1.49)	-0.00 (-0.00 to -0.00)	-0.06 (-0.10 to -0.01)
Avg years working for pay*	2.40 (2.31 to 2.49)	2.40 (2.31 to 2.50)	0.00 (0.00 to 0.00)	0.06 (0.03 to 0.10)
Avg earnings (x 1000, $2024, disc.)*	102 (96 to 108)	102 (96 to 108)	0.04 (0.01 to 0.07)	0.04 (0.01 to 0.07)
Avg earnings (x 1000, $2024, disc.)	94 (88 to 99)	94 (88 to 99)	0.06 (0.01 to 0.11)	0.06 (0.01 to 0.12)
Avg medical costs (x 1000, $2024, disc.)*	349 (338 to 359)	347 (336 to 357)	-1.92 (-2.35 to -1.49)	-0.55 (-0.67 to -0.43)
Avg medical costs (x 1000, $2024, disc.)	353 (343 to 363)	354 (344 to 365)	1.33 (0.60 to 2.06)	0.38 (0.18 to 0.58)
Avg age at death (years)	82.6 (82.4 to 82.9)	82.7 (82.5 to 83.0)	0.11 (0.09 to 0.14)	0.13 (0.10 to 0.16)
Avg life years	16.4 (16.1 to 16.6)	16.5 (16.2 to 16.7)	0.11 (0.09 to 0.14)	0.68 (0.53 to 0.83)
Avg QALYs	9.3 (8.9 to 9.7)	9.4 (9.0 to 9.8)	0.08 (0.06 to 0.10)	0.89 (0.65 to 1.14)
Avg QALYs (x 1000, 2024$, disc.)	1,072 (1,031 to 1,113)	1,080 (1,040 to 1,120)	7.9 (5.8 to 10.0)	0.74 (0.53 to 0.95)
Cohort total life years (x 1000)	79,433 (78,221 to 80,645)	79,972 (78,797 to 81,147)	539 (421 to 658)	0.68 (0.53 to 0.83)
Cohort total QALYs (x 1000)	45,098 (43,139 to 47,058)	45,500 (43,571 to 47,429)	402 (298 to 505)	0.89 (0.65 to 1.14)
Cohort total nursing home years* (x 1000)	5,223 (4,835 to 5,610)	5,071 (4,697 to 5,445)	-152 (-190 to -114)	-2.9 (-3.6 to -2.2)
Cohort total years claiming disability* (x 1000)	6,289 (6,032 to 6,546)	6,285 (6,028 to 6,543)	-3.4 (-6.0 to -0.9)	-0.06 (-0.10 to -0.01)
Cohort total years working for pay* (x 1000)	10,552 (10,140 to 10,963)	10,558 (10,146 to 10,971)	6.9 (2.7 to 11.1)	0.06 (0.03 to 0.10)
Cohort total earnings (millions, $2024, disc.)*	450,128 (423,867 to 476,389)	450,301 (424,029 to 476,573)	173 (38 to 308)	0.04 (0.01 to 0.07)
Cohort total earnings (millions, $2024, disc.)	454,325 (427,906 to 480,744)	454,607 (428,179 to 481,035)	282 (40 to 524)	0.06 (0.01 to 0.12)
Cohort total medical costs (millions, $2024, disc.)*	1,533,018 (1,486,292 to 1,579,744)	1,524,569 (1,478,326 to 1,570,811)	-8,449 (-10,348 to -6,550)	-0.55 (-0.67 to -0.43)
Cohort total medical costs (millions, $2024, disc.)	1,712,498 (1,662,873 to 1,762,124)	1,718,962 (1,668,844 to 1,769,080)	6,463 (2,909 to 10,018)	0.38 (0.18 to 0.58)
Cohort total QALYs (millions, $2024, disc.)	5,202,743 (5,004,213 to 5,401,273)	5,241,184 (5,045,722 to 5,436,645)	38,441 (28,270 to 48,611)	0.74 (0.53 to 0.95)

* Includes only waves in which respondent is alive in both status quo and counterfactual scenarios

Table S7p: Lifetime effects of sleep medication elimination for those with **some college education or more** (age 51+).

	Status quo: Using sleep medication at baseline	Counterfactual: No future sleep medication	Difference	Percentage difference
Sleep medication use (% at start)	100 (100 to 100)	100 (100 to 100)	0 (0 to 0)	(0 to 0)
Sleep medication use (avg lifetime %)	45.5 (44.4 to 46.7)	0.0 (0.0 to 0.0)	-45.5 (-46.7 to -44.4)	-100.0 (-100.0 to -100.0)
Sleep disturbance (JSS of 3 or more, % at start)	73.8 (73.8 to 73.8)	73.8 (73.8 to 73.8)	0.00 (0.00 to 0.00)	0.00 (0.00 to 0.00)
Injury due to fall (% ever)*	65.9 (64.6 to 67.1)	61.3 (60.1 to 62.6)	-4.54 (-5.21 to -3.87)	-6.89 (-7.88 to -5.90)
TICS cognitive score < 12 (% ever)*	70.2 (68.9 to 71.5)	68.3 (66.9 to 69.6)	-1.93 (-2.33 to -1.52)	-2.75 (-3.32 to -2.17)
Living in nursing home (% ever)*	25.1 (23.1 to 27.0)	24.6 (22.6 to 26.5)	-0.54 (-0.67 to -0.40)	-2.14 (-2.68 to -1.61)
Any limitations in ADLs (% ever)*	79.2 (78.1 to 80.3)	79.1 (78.0 to 80.2)	-0.11 (-0.15 to -0.06)	-0.13 (-0.19 to -0.08)
Any limitations in IADLs (% ever)*	76.9 (76.0 to 77.8)	76.6 (75.8 to 77.5)	-0.26 (-0.33 to -0.19)	-0.34 (-0.43 to -0.25)
Avg nr of survey waves with any injury due to fall*	1.80 (1.70 to 1.90)	1.57 (1.49 to 1.66)	-0.23 (-0.26 to -0.19)	-12.58 (-14.42 to -10.74)
Avg years with TICS cognitive score < 12*	4.77 (4.58 to 4.97)	4.49 (4.30 to 4.69)	-0.28 (-0.33 to -0.23)	-5.88 (-6.98 to -4.79)
Avg years in nursing home*	1.29 (1.14 to 1.43)	1.25 (1.10 to 1.39)	-0.04 (-0.05 to -0.03)	-2.88 (-3.59 to -2.18)
Avg years with limitations in any ADLs*	5.74 (5.51 to 5.98)	5.71 (5.48 to 5.95)	-0.03 (-0.04 to -0.02)	-0.56 (-0.70 to -0.42)
Avg years with limitations in any IADLs*	5.18 (5.00 to 5.37)	5.13 (4.94 to 5.31)	-0.06 (-0.07 to -0.04)	-1.10 (-1.32 to -0.87)
Avg years receiving disability benefits*	1.18 (1.13 to 1.22)	1.18 (1.13 to 1.22)	-0.00 (-0.00 to 0.00)	-0.02 (-0.06 to 0.02)
Avg years working for pay*	4.35 (4.22 to 4.47)	4.35 (4.23 to 4.47)	0.00 (0.00 to 0.00)	0.04 (0.01 to 0.06)
Avg earnings (x 1000, $2024, disc.)*	269 (258 to 280)	269 (258 to 280)	0.04 (-0.01 to 0.09)	0.01 (-0.00 to 0.03)
Avg earnings (x 1000, $2024, disc.)	255 (245 to 265)	255 (245 to 266)	0.06 (-0.02 to 0.14)	0.02 (-0.01 to 0.05)
Avg medical costs (x 1000, $2024, disc.)*	380 (367 to 394)	379 (366 to 392)	-1.74 (-2.14 to -1.35)	-0.46 (-0.56 to -0.36)
Avg medical costs (x 1000, $2024, disc.)	390 (377 to 404)	391 (378 to 405)	1.10 (0.44 to 1.76)	0.28 (0.12 to 0.45)
Avg age at death (years)	85.0 (84.8 to 85.3)	85.1 (84.9 to 85.4)	0.11 (0.08 to 0.14)	0.13 (0.10 to 0.16)
Avg life years	21.1 (20.9 to 21.4)	21.2 (21.0 to 21.5)	0.11 (0.08 to 0.14)	0.52 (0.39 to 0.65)
Avg QALYs	13.2 (12.8 to 13.6)	13.3 (12.9 to 13.7)	0.09 (0.06 to 0.11)	0.65 (0.46 to 0.84)
Avg QALYs (x 1000, 2024$, disc.)	1,466 (1,425 to 1,506)	1,473 (1,433 to 1,513)	7.5 (5.3 to 9.6)	0.51 (0.36 to 0.66)
Cohort total life years (x 1000)	160,256 (158,150 to 162,363)	161,085 (158,901 to 163,269)	828 (617 to 1,040)	0.52 (0.39 to 0.65)
Cohort total QALYs (x 1000)	100,316 (97,198 to 103,434)	100,963 (97,867 to 104,059)	647 (461 to 833)	0.65 (0.46 to 0.84)
Cohort total nursing home years* (x 1000)	9,181 (8,115 to 10,247)	8,916 (7,873 to 9,959)	-265 (-334 to -196)	-2.9 (-3.6 to -2.2)
Cohort total years claiming disability* (x 1000)	8,408 (8,082 to 8,734)	8,406 (8,080 to 8,733)	-1.4 (-4.7 to 1.9)	-0.02 (-0.06 to 0.02)
Cohort total years working for pay* (x 1000)	31,038 (30,170 to 31,905)	31,049 (30,181 to 31,916)	11.5 (3.5 to 19.4)	0.04 (0.01 to 0.06)
Cohort total earnings (millions, $2024, disc.)*	1,921,401 (1,842,944 to 1,999,859)	1,921,660 (1,843,167 to 2,000,153)	259 (-104 to 621)	0.01 (-0.00 to 0.03)
Cohort total earnings (millions, $2024, disc.)	1,934,278 (1,855,193 to 2,013,363)	1,934,749 (1,855,764 to 2,013,734)	471 (-119 to 1,061)	0.02 (-0.01 to 0.05)
Cohort total medical costs (millions, $2024, disc.)*	2,717,057 (2,622,669 to 2,811,445)	2,704,606 (2,611,151 to 2,798,062)	-12,451 (-15,269 to -9,633)	-0.46 (-0.56 to -0.36)
Cohort total medical costs (millions, $2024, disc.)	2,960,539 (2,859,030 to 3,062,049)	2,968,878 (2,865,671 to 3,072,085)	8,339 (3,319 to 13,358)	0.28 (0.12 to 0.45)
Cohort total QALYs (millions, $2024, disc.)	11,115,695 (10,809,962 to 11,421,428)	11,172,384 (10,868,664 to 11,476,104)	56,689 (40,242 to 73,136)	0.51 (0.36 to 0.66)

* Includes only waves in which respondent is alive in both status quo and counterfactual scenarios

Table S8: Prevalence of prescription sleep medication use in simulation starting cohort (age 51+) based on survey-weighted 2016 HRS data.

	**Mean (%)**	**95% CI**
Women	15.7	14.8-16.6
Men	10.9	10.0-11.7
Non-Hispanic Black	13.9	12.5-15.3
Hispanic	13.3	11.6-15.1
Non-Hispanic White	13.4	12.7-14.1
Less than high school	20.3	18.2-22.4
High school graduate	13.6	12.5-14.6
Some college or more	11.8	11.0-12.6
Age 51-54	12.1	10.3-14.0
Age 55-59	12.5	10.9-14.1
Age 60-64	12.8	11.5-14.2
Age 65-69	12.8	11.2-14.5
Age 70-74	13.3	11.6-15.1
Age 75-79	12.6	11.0-14.2
Age 80-84	16.3	14.3-18.2
Age 85+	20.0	18.1-21.0
