# Supplementary material for: Lifetime burden of prescription medication for insomnia in middle-aged and older adults in the US: a microsimulation study
Source: Lancet Reg Health Am. 2025 Oct 24;52:101284. doi: 10.1016/j.lana.2025.101284 (PMC12593590; doi:10.1016/j.lana.2025.101284)
Supplement: Supplementary data 2 [file mmc2.pdf]

This file provides supplementary details for:
Title: Lifetime Burden of Prescription Medication for Insomnia in Middle-Aged and Older Adults in the U.S.: A Microsimulation Study
Authors: Hanke Heun-Johnson, Johanna Thunell, Jonathan N. Cloughesy, Jeffrey A. Linder, Stephen D. Persell, Mark D. Sullivan, Bryan Tysinger, and Jason Doctor

The following sheets contain transition model estimates for relevant variables in the Future Elderly Model. The worksheets marked with Sleep Rx are expansions of the existing FEM framework.

Sleep Rx - Binaries
This worksheet reports estimates of the probability of an injury due to a fall, dying, living in a nursing home, and taking prescription sleep medication to help sleep

Sleep Rx - Ordered Probits
This worksheet reports estimates of the probability of changing ADL, IADL, PFL, and cognitive status

Sleep Rx - OLS
This worksheet reports estimates for HUI3 (Health Utilities Index Mark 3) score for QALYs

Sleep Rx - Imputations
This worksheet contains imputation models; one for imputing probability of an injury due to a fall for those under 65, using NHIS data, and one for predicting z-drug use among those who take prescription sleep medication to help sleep, using MEPS data

Sleep Rx + insomnia - Binaries
Insomnia is included in these models. This worksheet reports estimates of the probability of an injury due to a fall, and taking prescription sleep medication to help sleep.

Sleep Rx + insomnia - Ordered Probits
Insomnia is included in these models. This worksheet reports estimates of the probability of changing cognitive status

FEM - Binaries
This worksheet reports estimates of the probability of developing a chronic condition (stroke, heart disease, cancer, hypertension diabetes, lung disease), of having a heart attack, of claiming SSDI, SSI, OASI,working for pay, medicaid eligibility, and medicare enrollment

FEM - Ordered Probits
This worksheet reports estimates of the probability of changing smoking status

FEM - OLS
This worksheet reports estimates of how BMI and earnings are updated in the microsimulation.

Med costs
This worksheet contains models for medical costs based on MCBS and MEPS data

	Injury due to a fall (fallinj) coefficients		marginal effects		Died (died) coefficients		marginal effects		R living in nursing home (nhmliv) coefficients		marginal effects		Taking prescription sleep medication to help sleep (sleeprx) coefficients		marginal effects	
	coef	se	coef	se	coef	se	coef	se	coef	se	coef	se	coef	se	coef	se
Two-year lag of Injury due to a fall	0.763***	0.018	0.166		0.061***	0.023	0.005		0.069*	0.041	0.001					
Regularly taking Rx meds to help sleep	0.223***	0.017	0.036													
Two-year lag of Regularly taking Rx meds to help sleep													1.912***	0.015	0.578	
Male	-0.327***	0.026	-0.046		-0.667	0.642	-0.048		-0.111**	0.056	-0.001		-0.144***	0.025	-0.024	
Non-Hispanic Black	-0.306***	0.024	-0.039		-0.072**	0.030	-0.005		-0.482***	0.059	-0.003		-0.115***	0.023	-0.018	
Hispanic	-0.153***	0.029	-0.020		-0.209***	0.042	-0.014		-0.664***	0.084	-0.004		0.025	0.028	0.004	
Less than high school	0.064***	0.023	0.010		-0.117***	0.028	-0.008		-0.170***	0.050	-0.002		0.099***	0.024	0.017	
Some college and above	0.091***	0.018	0.013		-0.048*	0.025	-0.004		0.024	0.044	0.000		-0.014	0.019	-0.002	
Male AND Less than high school	-0.029	0.043	-0.004		0.092**	0.042	0.008		0.004	0.082	0.000		-0.024	0.040	-0.004	
Male AND Non-Hispanic Black	0.000	0.048	0.000		0.025	0.045	0.002		0.414***	0.087	0.007		0.111***	0.039	0.020	
Male AND Hispanic	0.045	0.052	0.007		0.041	0.061	0.003		0.259*	0.134	0.004		-0.041	0.046	-0.007	
Male AND Some college and above	-0.093***	0.033	-0.013		0.014	0.037	0.001		0.008	0.072	0.000		-0.008	0.031	-0.001	
Min(63, two-year lag of age)	0.014***	0.003	0.002		0.018**	0.007	0.001		0.024*	0.013	0.000		-0.007***	0.003	-0.001	
Min(Max(0, two-year lag age - 63), 73 - 63)	0.014***	0.002	0.002		0.027***	0.005	0.002		0.044***	0.007	0.000		-0.001	0.002	-0.000	
Max(0, two-year lag age - 73)	0.025***	0.002	0.004						0.045***	0.003	0.000		0.008***	0.002	0.001	
Two-year lag of Heart disease	0.139***	0.016	0.021		0.151***	0.027	0.012		0.011	0.033	0.000		0.134***	0.016	0.024	
Two-year lag of Stroke	0.107***	0.023	0.017		0.130***	0.030	0.011		0.208***	0.038	0.003		0.110***	0.023	0.019	
Two-year lag of Cancer	0.049***	0.018	0.007		0.387***	0.026	0.038		0.006	0.038	0.000		0.070***	0.018	0.012	
Two-year lag of Hypertension	0.041***	0.015	0.006		0.060**	0.025	0.005		-0.025	0.033	-0.000		0.134***	0.015	0.022	
Two-year lag of Diabetes	0.058***	0.016	0.009		0.116***	0.026	0.009		0.067*	0.036	0.001		0.041**	0.016	0.007	
Two-year lag of Lung disease	0.116***	0.021	0.018		0.222***	0.029	0.020		-0.025	0.046	-0.000		0.224***	0.021	0.042	
Two-year lag of R had heart attack since last wave	-0.005	0.049	-0.001		0.075	0.065	0.006						0.008	0.048	0.001	
Two-year lag of Current smoking	-0.041*	0.023	-0.006		0.178***	0.037	0.015						0.110***	0.020	0.019	
Splined two-year lag of BMI <= log(30)	-0.126**	0.056	-0.018										-0.095*	0.057	-0.016	
Splined two-year lag of BMI > log(30)	1.647***	0.065	0.240										0.036	0.072	0.006	
Log of years between current interview and previous	0.299***	0.037	0.044						0.921***	0.080	0.009		0.115***	0.036	0.019	
Min(Max(0, two-year lag age - 73), 83 - 73)					0.031***	0.004	0.002									
Max(0, two-year lag age - 83)					0.050***	0.004	0.004									
Two-year lag of TICS score < 7 (dementia)					0.559***	0.027	0.065		0.731***	0.046	0.019					
Two-year lag of TICS score 7-11 (mild cognitive impairment					0.270***	0.020	0.024		0.433***	0.036	0.007					
Two-year lag of Has exactly 1 IADL					0.262***	0.034	0.024		0.261***	0.045	0.004					
Two-year lag of Has 2 or more IADLs					0.347***	0.036	0.034		0.416***	0.047	0.007					
Two-year lag of Has exactly 1 ADL					0.166***	0.034	0.014		0.094**	0.046	0.001					
Two-year lag of Has exactly 2 ADLs					0.253***	0.043	0.024		0.233***	0.058	0.003					
Two-year lag of Has 3 or more ADLs					0.445***	0.037	0.048		0.321***	0.051	0.005					
Two-year lag of Widowed					0.056**	0.025	0.004		0.086**	0.035	0.001					
Heart problem status at age 50 (1/0)-imputed					0.001	0.096	0.000		0.262**	0.114	0.004					
Stroke status at age 50 (1/0)-imputed					-0.253*	0.139	-0.015		0.151	0.176	0.002					
Cancer status at age 50 (1/0)-imputed					-0.093*	0.053	-0.007		0.115	0.095	0.001					
Diabetes status at age 50 (imputed)					0.110**	0.045	0.009		0.078	0.069	0.001					
Init. of Ever smoked					0.054*	0.030	0.004		0.056	0.039	0.001					
Smoking status at age 50 (imputed)					0.181***	0.033	0.015		-0.000	0.041	-0.000					
Two-year lag of Ever had congestive heart failure					0.258***	0.037	0.024									
male_l2age65l					0.013	0.011	0.001									
male_l2age6574					0.000	0.007	0.000									
male_l2age7584					0.002	0.006	0.000									
male_l2age85p					0.024***	0.007	0.002									
Male AND Two-year lag of Ever had congestive heart failure					0.015	0.053	0.001									
Male AND Two-year lag of Heart disease					-0.073*	0.040	-0.005									
Male AND Two-year lag of Stroke					-0.092**	0.044	-0.007									
Male AND Two-year lag of Cancer					-0.077**	0.038	-0.006									
Male AND Two-year lag of Hypertension					0.047	0.036	0.004									
Male AND Two-year lag of Diabetes					0.011	0.038	0.001									
Male AND Two-year lag of Lung disease					0.112***	0.043	0.009									
Male AND Two-year lag of R had heart attack since last wave					0.021	0.088	0.002									
Male AND Two-year lag of Has exactly 1 IADL					-0.133***	0.051	-0.009									
Male AND Two-year lag of Has 2 or more IADLs					-0.081	0.055	-0.006									
Male AND Two-year lag of Has exactly 1 ADL					0.055	0.050	0.004									
Male AND Two-year lag of Has exactly 2 ADLs					0.063	0.066	0.005									
Male AND Two-year lag of Has 3 or more ADLs					0.103*	0.058	0.009									
Male AND Two-year lag of Current smoking					0.035	0.053	0.003									
Male AND Two-year lag of Widowed					0.010	0.042	0.001									
Male AND Heart problem status at age 50 (1/0)-imputed					0.171	0.125	0.015									
Male AND Stroke status at age 50 (1/0)-imputed					0.204	0.219	0.019									
Male AND Cancer status at age 50 (1/0)-imputed					-0.059	0.118	-0.004									
Male AND Diabetes status at age 50 (imputed)					0.068	0.067	0.006									
Male AND Init. of Ever smoked					-0.043	0.043	-0.003									
Male AND Smoking status at age 50 (imputed)					0.005	0.045	0.000									
Two-year lag of Non-pension wlth(hatotb) not zero									-0.188***	0.062	-0.002					
Two-year lag of (IHT of hh wlth in 1000s)/100, zero otherwise									-3.639***	0.602	-0.037					
Two-year lag of R living in nursing home									2.339***	0.063	0.343					
_cons	-2.296***	0.273			-3.657***	0.418			-4.887***	0.800			-1.037***	0.242		
Number of observations	76,410		76,410		82,810		82,810		76,953		76,953		78,202		78,202	
Pseudo R2	0.129		0.129		0.264		0.264		0.517		0.517		0.310		0.310	

note: .01 - ***; .05 - **; .1 - *;

Sleep Rx - Ordered probits

	ADL status (adlstat) coefficients		ADL status (adlstat) marginal effects				IADL status (iadlstat) coefficients		IADL status (iadlstat) marginal effects			PFL status (pflstat) coefficients		PFL status (pflstat) marginal effects			Cognitive state (cogstate) coefficients		Cognitive state (cogstate) marginal effects		
	coef	se	coef	coef	coef	coef	coef	se	coef	coef	coef	coef	se	coef	coef	coef	coef	se	coef	coef	coef
Two-year lag of TICS score < 7 (dementia)							0.740***	0.026	-0.196	0.105	0.091						-2.160***	0.023	0.431	0.289	-0.719
Two-year lag of TICS score 7-11 (mild cognitive impairment							0.322***	0.016	-0.069	0.042	0.027						-1.047***	0.013	0.084	0.262	-0.346
Two-year lag of Has exactly 1 PFL												0.934***	0.012	-0.337	0.006	0.331					
Two-year lag of Has 2 or more PFLs												1.702***	0.014	-0.561	-0.016	0.577					
Regularly taking Rx meds to help sleep																	-0.200***	0.014	0.008	0.048	-0.056
Two-year lag of Injury due to a fall	0.108***	0.019	-0.026	0.014	0.006	0.006	0.097***	0.020	-0.019	0.012	0.007	0.027	0.018	-0.011	0.002	0.008					
Non-Hispanic Black	0.134***	0.020	-0.032	0.017	0.008	0.007	0.027	0.022	-0.005	0.003	0.002	0.088***	0.018	-0.035	0.007	0.028	-0.435***	0.018	0.021	0.107	-0.128
Hispanic	0.220***	0.024	-0.055	0.029	0.014	0.013	0.066**	0.027	-0.013	0.008	0.005	0.085***	0.022	-0.034	0.007	0.027	-0.330***	0.021	0.015	0.080	-0.096
Less than high school	0.116***	0.020	-0.028	0.015	0.007	0.006	0.045**	0.022	-0.009	0.005	0.003	0.112***	0.019	-0.044	0.009	0.035	-0.336***	0.018	0.015	0.081	-0.096
Some college and above	-0.050***	0.017	0.011	-0.006	-0.003	-0.002	-0.010	0.019	0.002	-0.001	-0.001	-0.093***	0.014	0.037	-0.009	-0.028	0.262***	0.016	-0.009	-0.059	0.068
Male	-0.011	0.022	0.002	-0.001	-0.001	-0.001	-0.044*	0.024	0.008	-0.005	-0.003	-0.198***	0.018	0.079	-0.019	-0.060	-0.129***	0.020	0.005	0.029	-0.034
Male AND Less than high school	0.011	0.033	-0.003	0.001	0.001	0.001	0.050	0.035	-0.010	0.006	0.003	-0.041	0.030	0.016	-0.004	-0.012	0.001	0.028	-0.000	-0.000	0.000
Male AND Non-Hispanic Black	-0.045	0.032	0.010	-0.006	-0.002	-0.002	0.040	0.035	-0.008	0.005	0.003	-0.021	0.028	0.008	-0.002	-0.006	0.051*	0.027	-0.002	-0.011	0.013
Male AND Hispanic	-0.086**	0.038	0.019	-0.011	-0.005	-0.004	0.048	0.042	-0.009	0.006	0.003	-0.056*	0.034	0.022	-0.005	-0.017	0.116***	0.032	-0.004	-0.025	0.029
Male AND Some college and above	-0.014	0.027	0.003	-0.002	-0.001	-0.001	-0.080***	0.030	0.014	-0.009	-0.005	-0.027	0.022	0.011	-0.002	-0.008	0.039	0.025	-0.001	-0.009	0.010
Min(63, two-year lag of age)	0.005*	0.003	-0.001	0.001	0.000	0.000	-0.009***	0.003	0.002	-0.001	-0.001	0.009***	0.002	-0.004	0.001	0.003	-0.006***	0.002	0.000	0.001	-0.002
Min(Max(0, two-year lag age - 63), 73 - 63)	0.016***	0.002	-0.004	0.002	0.001	0.001	0.018***	0.002	-0.003	0.002	0.001	0.023***	0.002	-0.009	0.002	0.007	-0.028***	0.002	0.001	0.006	-0.007
Max(0, two-year lag age - 73)	0.036***	0.002	-0.008	0.005	0.002	0.002	0.043***	0.002	-0.008	0.005	0.003	0.033***	0.002	-0.013	0.003	0.010	-0.049***	0.001	0.002	0.011	-0.013
Two-year lag of Heart disease	0.106***	0.014	-0.025	0.014	0.006	0.005	0.106***	0.015	-0.021	0.013	0.008	0.172***	0.013	-0.068	0.014	0.054	0.001	0.014	-0.000	-0.000	0.000
Two-year lag of Stroke	0.243***	0.019	-0.062	0.032	0.015	0.014	0.226***	0.020	-0.047	0.029	0.018	0.153***	0.020	-0.061	0.012	0.049	-0.245***	0.018	0.011	0.059	-0.070
Two-year lag of Cancer	0.061***	0.017	-0.014	0.008	0.004	0.003	0.060***	0.018	-0.012	0.007	0.004	0.047***	0.015	-0.019	0.004	0.015	0.018	0.016	-0.001	-0.004	0.005
Two-year lag of Hypertension	0.045***	0.013	-0.010	0.006	0.003	0.002	0.065***	0.014	-0.012	0.008	0.004	0.115***	0.011	-0.046	0.011	0.035	-0.032***	0.012	0.001	0.007	-0.008
Two-year lag of Diabetes	0.056***	0.015	-0.013	0.007	0.003	0.003	0.063***	0.016	-0.012	0.008	0.004	0.076***	0.013	-0.030	0.007	0.024	-0.087***	0.014	0.003	0.020	-0.023
Two-year lag of Lung disease	0.209***	0.018	-0.053	0.028	0.013	0.012	0.233***	0.019	-0.049	0.030	0.019	0.307***	0.018	-0.120	0.018	0.102	-0.080***	0.018	0.003	0.018	-0.021
Two-year lag of R had heart attack since last wave	0.042	0.040	-0.010	0.005	0.002	0.002	0.057	0.043	-0.011	0.007	0.004	0.016	0.040	-0.006	0.001	0.005	-0.077**	0.039	0.003	0.018	-0.021
Two-year lag of Has exactly 1 IADL	0.423***	0.019	-0.116	0.057	0.029	0.029	0.989***	0.018	-0.281	0.139	0.141	0.289***	0.021	-0.113	0.017	0.096					
Two-year lag of Has 2 or more IADLs	0.729***	0.022	-0.221	0.098	0.057	0.067	1.606***	0.024	-0.517	0.188	0.329	0.440***	0.030	-0.169	0.017	0.152					
Two-year lag of Has exactly 1 ADL	0.982***	0.017	-0.313	0.125	0.080	0.107	0.443***	0.019	-0.102	0.060	0.042	0.363***	0.019	-0.141	0.018	0.122					
Two-year lag of Has exactly 2 ADLs	1.386***	0.024	-0.478	0.143	0.118	0.217	0.538***	0.027	-0.133	0.075	0.057	0.499***	0.032	-0.189	0.014	0.175					
Two-year lag of Has 3 or more ADLs	1.913***	0.024	-0.652	0.125	0.141	0.387	0.738***	0.026	-0.196	0.105	0.091	0.796***	0.036	-0.285	-0.005	0.290					
Two-year lag of Current smoking	0.094***	0.021	-0.023	0.012	0.006	0.005	0.149***	0.022	-0.030	0.019	0.011	0.148***	0.018	-0.059	0.011	0.047	-0.078***	0.019	0.003	0.018	-0.021
Two-year lag of Widowed	0.038**	0.016	-0.009	0.005	0.002	0.002	0.007	0.017	-0.001	0.001	0.000	0.044***	0.014	-0.017	0.004	0.014	-0.046***	0.015	0.002	0.010	-0.012
Heart problem status at age 50 (1/0)-imputed	0.042	0.052	-0.010	0.005	0.002	0.002	-0.019	0.058	0.004	-0.002	-0.001	0.077	0.049	-0.030	0.006	0.024	-0.080	0.049	0.003	0.019	-0.022
Stroke status at age 50 (1/0)-imputed	0.085	0.070	-0.020	0.011	0.005	0.004	0.016	0.075	-0.003	0.002	0.001	0.125*	0.073	-0.049	0.009	0.040	-0.134**	0.057	0.005	0.032	-0.037
Cancer status at age 50 (1/0)-imputed	0.049	0.034	-0.011	0.006	0.003	0.002	-0.002	0.038	0.000	-0.000	-0.000	0.019	0.030	-0.007	0.002	0.006	0.019	0.034	-0.001	-0.004	0.005
Diabetes status at age 50 (imputed)	0.149***	0.024	-0.037	0.020	0.009	0.008	0.094***	0.027	-0.018	0.012	0.007	0.131***	0.023	-0.052	0.010	0.042	-0.048**	0.023	0.002	0.011	-0.013
Init. of Ever smoked	0.004	0.015	-0.001	0.000	0.000	0.000	0.022	0.016	-0.004	0.003	0.002	0.009	0.012	-0.004	0.001	0.003	0.019	0.014	-0.001	-0.004	0.005
Smoking status at age 50 (imputed)	0.078***	0.017	-0.018	0.010	0.004	0.004	0.024	0.019	-0.004	0.003	0.002	0.125***	0.015	-0.050	0.011	0.039	-0.073***	0.016	0.003	0.017	-0.019
Splined two-year lag of BMI <= log(30)	-0.358***	0.065	0.083	-0.046	-0.020	-0.017	-0.681***	0.069	0.127	-0.082	-0.046	-0.135**	0.058	0.054	-0.012	-0.041	0.886***	0.060	-0.031	-0.201	0.232
Splined two-year lag of BMI > log(30)	0.541***	0.077	-0.125	0.069	0.030	0.026	0.273***	0.085	-0.051	0.033	0.018	0.615***	0.077	-0.245	0.056	0.189	0.155**	0.079	-0.005	-0.035	0.040
Splined init of BMI age 50 <= log(30)	0.593***	0.068	-0.137	0.075	0.033	0.028	0.482***	0.073	-0.090	0.058	0.032	0.466***	0.059	-0.186	0.043	0.143	-0.502***	0.062	0.017	0.114	-0.131
Splined init of BMI age 50 > log(30)	0.335***	0.084	-0.077	0.043	0.019	0.016	0.334***	0.092	-0.063	0.040	0.023	0.399***	0.083	-0.159	0.037	0.122	-0.332***	0.084	0.012	0.075	-0.087
Log of years between current interview and previous	0.331***	0.031	-0.076	0.042	0.019	0.016	0.391***	0.034	-0.073	0.047	0.026	0.226***	0.027	-0.090	0.021	0.069	-0.256***	0.026	0.009	0.058	-0.067
cut1	3.077***	0.253					1.044***	0.273				2.877***	0.208				-2.179***	0.200			
cut2	3.672***	0.253					1.700***	0.273				3.666***	0.208				-0.890***	0.200			
cut3	4.092***	0.253																			
Number of observations	76,953						76,953								76,953		86,348				
Pseudo R2	0.277						0.325								0.328		0.305				

note: .01 - ***; .05 - **; .1 - *;

	QALYs (HUI3) (hui3ou_i) coefficients		QALYs (HUI3) (hui3ou_i) marginal effects
	coef	se	coef
Has exactly 1 IADL	-0.226***	0.028	-0.226
Has 2 or more IADLs	-0.321***	0.042	-0.321
Has exactly 1 ADL	-0.247***	0.024	-0.247
Has exactly 2 ADLs	-0.285***	0.034	-0.285
Has 3 or more ADLs	-0.270***	0.043	-0.270
Cancer	0.003	0.019	0.003
Diabetes	-0.036*	0.019	-0.036
Heart disease	-0.053***	0.018	-0.053
Hypertension	0.001	0.013	0.001
Lung disease	-0.082***	0.023	-0.082
Stroke	-0.095***	0.025	-0.095
R had heart attack since last wave	0.011	0.044	0.011
Current smoking	-0.015	0.017	-0.015
Obese	-0.005	0.015	-0.005
Single	-0.011	0.020	-0.011
Widowed	-0.006	0.016	-0.006
TICS score < 7 (dementia)	-0.066*	0.034	-0.066
TICS score 7-11 (mild cognitive impairment)	-0.077***	0.018	-0.077
_cons	0.865***	0.011	
Number of observations	1,001		
Adjusted R2	0.444		

note: .01 - ***; .05 - **; .1 - *;

	Injury due to fall (imputation 51-64 yrs) (fallinj) coefficients		Injury due to fall (imputation 51-64 yrs) (fallinj) marginal effects	
	coef	se	coef	se
age: (.,55)	0.017	0.011	0.001	
age: (55,60)	0.006	0.009	0.000	
age: (60,65)	-0.003	0.009	-0.000	
age: (65,70)	0.010	0.010	0.000	
age: (70,75)	0.004	0.011	0.000	
age: (75,80)	0.023**	0.012	0.001	
age: (80,.)	0.023	0.018	0.001	
male	-0.233***	0.020	-0.008	
black	-0.133***	0.029	-0.004	
hispan	-0.141***	0.033	-0.005	
hsless	0.052*	0.029	0.002	
college	0.023	0.022	0.001	
logbmi: (.,3.4011974)	0.104	0.076	0.004	
logbmi: (3.401197381662155,.)	0.610***	0.088	0.022	
_cons	-3.433***	0.614		
Number of observations	121,146		121,146	
Pseudo R2	0.017		0.017	

note: .01 - ***; .05 - **; .1 - *;

	Z-drug use (z_drug) coefficients		Z-drug use (z_drug) marginal effects	
	coef	se	coef	se
age: (.,55)	0.006	0.021	0.002	
age: (55,60)	0.008	0.017	0.002	
age: (60,65)	-0.006	0.018	-0.002	
age: (65,70)	-0.022	0.019	-0.006	
age: (70,75)	0.009	0.023	0.003	
age: (75,80)	-0.004	0.028	-0.001	
age: (80,85)	-0.008	0.048	-0.002	
age: (85,.)	-0.085	0.203	-0.024	
male	0.040	0.038	0.012	
black	0.175***	0.057	0.054	
hispan	0.043	0.053	0.013	
Less than HS	0.023	0.051	0.007	
college	0.232***	0.041	0.070	
logbmi: (.,3.4011974)	0.052	0.071	0.015	
logbmi: (3.401197381662155,.)	0.083	0.158	0.024	
_cons	-1.367	1.123		
Number of observations	6,140		6,140	
Pseudo R2	0.009		0.009	

note: .01 - ***; .05 - **; .1 - *;

Sleep Rx - Imputations

	Injury due to a fall (fallinj) coefficients		marginal effects		Taking prescription sleep medication to help sleep (sleeprx) coefficients		marginal effects	
	coef	se	coef	se	coef	se	coef	se
Two-year lag of Injury due to a fall	0.779***	0.022	0.167					
Regularly taking Rx meds to help sleep	0.200***	0.042	0.031					
Two-year lag of Regularly taking Rx meds to help sleep					1.854***	0.019	0.544	
Sleep disturbance (score of 3+ on Jenkins Sleep Scale [0-8])	0.074***	0.018	0.010		0.496***	0.018	0.079	
Regularly taking Rx meds to help sleep AND Sleep disturbance (score of 3+ on Jen	0.004	0.049	0.001					
Male	-0.359***	0.033	-0.049		-0.102***	0.032	-0.016	
Non-Hispanic Black	-0.313***	0.029	-0.038		-0.117***	0.029	-0.017	
Hispanic	-0.177***	0.035	-0.023		-0.022	0.034	-0.003	
Less than high school	0.064**	0.030	0.009		0.107***	0.030	0.018	
Some college and above	0.094***	0.022	0.013		0.031	0.024	0.005	
Male AND Less than high school	-0.002	0.055	-0.000		-0.036	0.051	-0.005	
Male AND Non-Hispanic Black	0.016	0.057	0.002		0.153***	0.047	0.026	
Male AND Hispanic	0.048	0.063	0.007		-0.037	0.056	-0.006	
Male AND Some college and above	-0.053	0.041	-0.007		-0.014	0.039	-0.002	
Min(63, two-year lag of age)	0.014***	0.004	0.002		-0.003	0.003	-0.000	
Min(Max(0, two-year lag age - 63), 73 - 63)	0.014***	0.003	0.002		-0.002	0.003	-0.000	
Max(0, two-year lag age - 73)	0.026***	0.002	0.004		0.008***	0.002	0.001	
Two-year lag of Heart disease	0.138***	0.020	0.021		0.123***	0.021	0.020	
Two-year lag of Stroke	0.104***	0.028	0.016		0.085***	0.029	0.014	
Two-year lag of Cancer	0.052**	0.022	0.008		0.071***	0.023	0.012	
Two-year lag of Hypertension	0.031*	0.019	0.004		0.126***	0.019	0.020	
Two-year lag of Diabetes	0.044**	0.020	0.006		0.014	0.020	0.002	
Two-year lag of Lung disease	0.102***	0.027	0.015		0.163***	0.026	0.028	
Two-year lag of R had heart attack since last wave	0.002	0.062	0.000		-0.053	0.062	-0.008	
Two-year lag of Current smoking	-0.001	0.028	-0.000		0.105***	0.025	0.017	
Splined two-year lag of BMI <= log(30)	-0.093	0.071	-0.013		-0.091	0.071	-0.014	
Splined two-year lag of BMI > log(30)	1.732***	0.079	0.245		-0.017	0.088	-0.003	
Log of years between current interview and previous	0.278***	0.047	0.039		0.154***	0.046	0.024	
_cons	-2.447***	0.340			-1.606***	0.304		
Number of observations	50,433		50,433		51,627		51,627	
Pseudo R2	0.136		0.136		0.335		0.335	

note: .01 - ***; .05 - **; .1 - *;

	Cognitive state (cogstate) coefficients		marginal effects		
	coef	se	coef	coef	coef
Regularly taking Rx meds to help sleep	-0.283***	0.035	0.012	0.068	-0.080
Two-year lag of TICS score < 7 (dementia)	-2.159***	0.029	0.426	0.293	-0.719
Two-year lag of TICS score 7-11 (mild cognitive impairment	-1.053***	0.016	0.082	0.264	-0.346
Sleep disturbance (score of 3+ on Jenkins Sleep Scale [0-8])	-0.018	0.015	0.001	0.004	-0.005
Regularly taking Rx meds to help sleep AND Sleep disturbance (score of 3+ on Jen	0.081**	0.041	-0.003	-0.018	0.020
Non-Hispanic Black	-0.441***	0.022	0.020	0.107	-0.128
Hispanic	-0.337***	0.027	0.015	0.082	-0.097
Less than high school	-0.312***	0.023	0.013	0.075	-0.088
Some college and above	0.258***	0.021	-0.009	-0.058	0.067
Male	-0.145***	0.025	0.005	0.033	-0.038
Male AND Less than high school	-0.008	0.036	0.000	0.002	-0.002
Male AND Non-Hispanic Black	0.065**	0.033	-0.002	-0.014	0.016
Male AND Hispanic	0.128***	0.040	-0.004	-0.027	0.031
Male AND Some college and above	0.044	0.031	-0.001	-0.010	0.011
Min(63, two-year lag of age)	-0.008***	0.003	0.000	0.002	-0.002
Min(Max(0, two-year lag age - 63), 73 - 63)	-0.028***	0.002	0.001	0.006	-0.007
Max(0, two-year lag age - 73)	-0.049***	0.002	0.002	0.011	-0.013
Two-year lag of Heart disease	0.002	0.017	-0.000	-0.000	0.001
Two-year lag of Stroke	-0.248***	0.023	0.011	0.060	-0.070
Two-year lag of Cancer	0.019	0.020	-0.001	-0.004	0.005
Two-year lag of Hypertension	-0.024	0.015	0.001	0.005	-0.006
Two-year lag of Diabetes	-0.057***	0.017	0.002	0.013	-0.015
Two-year lag of Lung disease	-0.099***	0.022	0.004	0.023	-0.026
Two-year lag of R had heart attack since last wave	-0.118**	0.050	0.005	0.028	-0.032
Two-year lag of Current smoking	-0.088***	0.024	0.003	0.020	-0.023
Two-year lag of Widowed	-0.061***	0.018	0.002	0.014	-0.016
Heart problem status at age 50 (1/0)-imputed	-0.094	0.063	0.004	0.022	-0.025
Stroke status at age 50 (1/0)-imputed	-0.158**	0.070	0.006	0.037	-0.044
Cancer status at age 50 (1/0)-imputed	0.026	0.042	-0.001	-0.006	0.007
Diabetes status at age 50 (imputed)	-0.087***	0.028	0.003	0.020	-0.023
Init. of Ever smoked	0.032*	0.017	-0.001	-0.007	0.008
Smoking status at age 50 (imputed)	-0.055***	0.020	0.002	0.013	-0.014
Splined two-year lag of BMI <= log(30)	0.851***	0.075	-0.029	-0.191	0.220
Splined two-year lag of BMI > log(30)	0.205**	0.097	-0.007	-0.046	0.053
Splined init of BMI age 50 <= log(30)	-0.518***	0.078	0.017	0.116	-0.134
Splined init of BMI age 50 > log(30)	-0.380***	0.103	0.013	0.085	-0.098
Log of years between current interview and previous	-0.335***	0.034	0.011	0.075	-0.086
cut1	-2.583***	0.258			
cut2	-1.291***	0.258			
Number of observations	54,563			54,563	
Pseudo R2	0.302			0.302	

note: .01 - ***; .05 - **; .1 - *;

	Died (died) coefficients		Died (died) marginal effects		Heart disease (hearte) coefficients		Heart disease (hearte) marginal effects		Stroke (stroke) coefficients		Stroke (stroke) marginal effects		Cancer (cancre) coefficients		Cancer (cancre) marginal effects		Hypertension (hibpe) coefficients		Hypertension (hibpe) marginal effects		Diabetes (diabe) coefficients		Diabetes (diabe) marginal effects		Lung disease (lunge) coefficients		Lung disease (lunge) marginal effects		Heart Attack since last wave (hearta) coefficients		Heart Attack since last wave (hearta) marginal effects	
	coef	se	coef	se	coef	se	coef	se	coef	se	coef	se	coef	se	coef	se	coef	se	coef	se	coef	se	coef	se	coef	se	coef	se	coef	se	coef	se
Non-Hispanic Black	0.009	0.022	0.001		-0.078***	0.025	-0.006		0.082***	0.030	0.003		-0.119***	0.030	-0.006		0.185***	0.027	0.032		0.131***	0.025	0.009		-0.066**	0.031	-0.002		-0.086*	0.049	-0.012	
Hispanic	-0.144***	0.031	-0.009		-0.119***	0.032	-0.009		-0.067	0.042	-0.002		-0.169***	0.041	-0.008		0.053*	0.030	0.009		0.322***	0.030	0.026		-0.121***	0.042	-0.004		0.155**	0.066	0.024	
Less than high school	0.021	0.020	0.002		0.044*	0.024	0.003		0.040	0.030	0.001		-0.013	0.030	-0.001		0.110***	0.026	0.018		0.081***	0.027	0.005		0.135***	0.030	0.005		0.100**	0.044	0.014	
Some college and above	-0.064***	0.019	-0.005		-0.060***	0.021	-0.005		-0.032	0.026	-0.001		0.052**	0.024	0.003		-0.058***	0.020	-0.009		-0.028	0.022	-0.002		-0.047*	0.027	-0.002		-0.063*	0.038	-0.009	
Male	-0.439	0.358	-0.030		0.157***	0.025	0.013		0.045	0.032	0.002		0.129***	0.027	0.007		0.001	0.025	0.000		0.089***	0.027	0.006		0.066**	0.031	0.002		0.248***	0.040	0.035	
Male AND Less than high school	0.003	0.030	0.000		-0.048	0.038	-0.004		0.052	0.046	0.002		0.052	0.043	0.003		-0.075*	0.040	-0.011		-0.050	0.041	-0.003		-0.096**	0.046	-0.003		-0.113*	0.065	-0.015	
Male AND Non-Hispanic Black	0.058*	0.033	0.004		-0.136***	0.040	-0.010		-0.031	0.047	-0.001		0.145***	0.043	0.009		0.049	0.042	0.008		0.019	0.040	0.001		-0.036	0.049	-0.001		0.065	0.074	0.009	
Male AND Hispanic	0.067	0.045	0.005		-0.074	0.049	-0.005		0.014	0.063	0.000		-0.037	0.058	-0.002		0.080*	0.045	0.013		-0.127***	0.046	-0.007		-0.063	0.064	-0.002		-0.061	0.096	-0.008	
Male AND Some college and above	-0.017	0.028	-0.001		0.040	0.031	0.003		0.034	0.040	0.001		-0.033	0.034	-0.002		0.001	0.031	0.000		0.024	0.034	0.002		-0.103**	0.040	-0.003		-0.077	0.048	-0.010	
Min(63, two-year lag of age)	0.022***	0.004	0.002		0.018***	0.003	0.001		0.018***	0.004	0.001		0.023***	0.003	0.001		0.010***	0.002	0.002		0.014***	0.002	0.001		0.012***	0.003	0.000		-0.007	0.005	-0.001	
Min(Max(0, two-year lag age - 63), 73 - 63)	0.029***	0.003	0.002		0.021***	0.002	0.002		0.021***	0.003	0.001		0.017***	0.003	0.001		0.007***	0.002	0.001		-0.003	0.003	-0.000		0.018***	0.003	0.001		-0.013***	0.004	-0.002	
Min(Max(0, two-year lag age - 73), 83 - 73)	0.042***	0.003	0.003																													
Max(0, two-year lag age - 83)	0.057***	0.003	0.004																													
Two-year lag of Heart disease	0.131***	0.021	0.010						0.189***	0.020	0.008																					
Two-year lag of Stroke	0.168***	0.023	0.014																										0.064**	0.029	0.009	
Two-year lag of Cancer	0.407***	0.020	0.038						-0.027	0.026	-0.001																					
Two-year lag of Hypertension	0.086***	0.018	0.006		0.192***	0.014	0.015		0.146***	0.019	0.005																		-0.032	0.028	-0.005	
Two-year lag of Diabetes	0.167***	0.020	0.013		0.120***	0.019	0.010		0.123***	0.023	0.005						0.135***	0.024	0.023										0.067**	0.032	0.010	
Two-year lag of Lung disease	0.283***	0.023	0.025																													
Two-year lag of R had heart attack since last wave	0.055	0.048	0.004						0.148***	0.053	0.006																		0.600***	0.037	0.118	
Two-year lag of Has exactly 1 IADL	0.283***	0.026	0.025																													
Two-year lag of Has 2 or more IADLs	0.543***	0.026	0.058																													
Two-year lag of Has exactly 1 ADL	0.156***	0.025	0.012																										0.042	0.032	0.006	
Two-year lag of Has exactly 2 ADLs	0.211***	0.032	0.018																										0.082*	0.043	0.012	
Two-year lag of Has 3 or more ADLs	0.438***	0.028	0.044																										0.081**	0.037	0.012	
Two-year lag of Current smoking	0.279***	0.027	0.024		0.111***	0.023	0.009		0.208***	0.029	0.009		0.072***	0.026	0.004		0.088***	0.023	0.015		-0.043*	0.026	-0.003		0.334***	0.025	0.015		0.333***	0.041	0.056	
Two-year lag of Widowed	0.047**	0.018	0.003		0.024	0.019	0.002		0.059***	0.023	0.002		-0.025	0.022	-0.001		0.089***	0.021	0.015		0.040*	0.022	0.003		0.038	0.024	0.001		0.031	0.034	0.004	
Heart problem status at age 50 (1/0)-imputed	-0.042	0.073	-0.003						0.251***	0.065	0.011		0.070	0.065	0.004		0.067	0.071	0.011		0.166***	0.062	0.012		0.232***	0.067	0.010		-0.394***	0.110	-0.042	
Stroke status at age 50 (1/0)-imputed	-0.229**	0.104	-0.013		0.241**	0.094	0.023						0.033	0.115	0.002		-0.153	0.126	-0.022		0.080	0.094	0.005		0.485***	0.088	0.028		0.025	0.132	0.004	
Cancer status at age 50 (1/0)-imputed	-0.150***	0.043	-0.009		0.141***	0.040	0.012		0.140***	0.054	0.006						0.044	0.039	0.007		-0.000	0.043	-0.000		0.225***	0.045	0.010		0.075	0.072	0.011	
Diabetes status at age 50 (imputed)	0.072**	0.035	0.005		0.168***	0.031	0.015		0.116***	0.037	0.005		0.044	0.034	0.002		0.172***	0.038	0.030						0.154***	0.035	0.006		0.146***	0.051	0.022	
Init. of Ever smoked	0.209***	0.020	0.014		0.042**	0.017	0.003		0.030	0.022	0.001		0.087***	0.019	0.005		-0.012	0.017	-0.002		-0.032*	0.019	-0.002		0.207***	0.025	0.007		0.025	0.032	0.003	
Smoking status at age 50 (imputed)	-0.157***	0.024	-0.011		0.075***	0.020	0.006		-0.003	0.025	-0.000		-0.002	0.022	-0.000		0.018	0.021	0.003		0.134***	0.022	0.009		0.250***	0.024	0.010		0.052	0.035	0.007	
Two-year lag of Ever had congestive heart failure	0.254***	0.029	0.022																													
male_l2age65l	0.010*	0.006	0.001																													
male_l2age6574	0.001	0.005	0.000																													
male_l2age7584	-0.003	0.005	-0.000																													
male_l2age85p	0.011**	0.006	0.001																													
Male AND Two-year lag of Ever had congestive heart failure	0.015	0.042	0.001																													
Male AND Two-year lag of Heart disease	-0.047	0.029	-0.003																													
Male AND Two-year lag of Stroke	-0.054	0.033	-0.004																													
Male AND Two-year lag of Cancer	-0.067**	0.029	-0.004																													
Male AND Two-year lag of Hypertension	-0.010	0.026	-0.001																													
Male AND Two-year lag of Diabetes	-0.023	0.029	-0.002																													
Male AND Two-year lag of Lung disease	0.101***	0.033	0.008																													
Male AND Two-year lag of R had heart attack since last wave	0.120*	0.064	0.009																													
Male AND Two-year lag of Has exactly 1 IADL	-0.087**	0.038	-0.006																													
Male AND Two-year lag of Has 2 or more IADLs	-0.052	0.041	-0.004																													
Male AND Two-year lag of Has exactly 1 ADL	0.068*	0.037	0.005																													
Male AND Two-year lag of Has exactly 2 ADLs	0.122**	0.049	0.010																													
Male AND Two-year lag of Has 3 or more ADLs	0.115***	0.044	0.009																													
Male AND Two-year lag of Current smoking	0.030	0.038	0.002																													
Male AND Two-year lag of Widowed	0.024	0.032	0.002																													
Male AND Heart problem status at age 50 (1/0)-imputed	0.146	0.094	0.012																													
Male AND Stroke status at age 50 (1/0)-imputed	0.081	0.161	0.006																													
Male AND Cancer status at age 50 (1/0)-imputed	0.060	0.087	0.005																													
Male AND Diabetes status at age 50 (imputed)	0.038	0.052	0.003																													
Male AND Init. of Ever smoked	-0.068**	0.030	-0.005																													
Male AND Smoking status at age 50 (imputed)	0.102***	0.032	0.008																													
Max(0, two-year lag age - 73)					0.017***	0.002	0.001		0.027***	0.002	0.001		-0.002	0.002	-0.000		0.001	0.002	0.000		-0.007***	0.003	-0.000		0.006**	0.002	0.000		0.001	0.003	0.000	
Splined two-year lag of BMI <= log(30)					-0.042	0.084	-0.003		-0.318***	0.099	-0.011		-0.084	0.094	-0.005		0.588***	0.087	0.093		1.134***	0.105	0.070		-0.292***	0.104	-0.010		-0.018	0.097	-0.003	
Splined two-year lag of BMI > log(30)					0.276**	0.108	0.022		0.030	0.131	0.001		-0.093	0.128	-0.005		0.464***	0.133	0.074		1.168***	0.107	0.072		0.898***	0.131	0.030		-0.488***	0.143	-0.068	
Splined init of BMI age 50 <= log(30)					0.131	0.084	0.010		0.245**	0.102	0.008		0.134	0.094	0.007		0.092	0.087	0.015		0.645***	0.100	0.040		0.070	0.105	0.002					
Splined init of BMI age 50 > log(30)					0.241**	0.115	0.019		0.324**	0.137	0.011		0.232*	0.135	0.013		-0.059	0.143	-0.009		-0.226*	0.117	-0.014		0.031	0.144	0.001					
Log of years between current interview and previous					0.265***	0.039	0.021		0.368***	0.050	0.013		0.324***	0.044	0.018		0.314***	0.039	0.050		0.350***	0.042	0.022		0.183***	0.050	0.006		0.376***	0.074	0.053	
black_l2heartae																													0.199**	0.084	0.032	
hispan_l2heartae																													-0.006	0.110	-0.001	
hsless_l2heartae																													-0.025	0.072	-0.003	
college_l2heartae																													0.035	0.051	0.005	
male_l2heartae																													-0.109*	0.058	-0.015	
male_hsless_l2heartae																													-0.004	0.099	-0.001	
male_black_l2heartae																													-0.104	0.122	-0.014	
male_hispan_l2heartae																													-0.162	0.161	-0.020	
l2age65l_l2heartae																													0.009	0.007	0.001	
l2age6574_l2heartae																													0.010	0.007	0.001	
l2age75p_l2heartae																													0.004	0.005	0.001	
l2hibpe_l2heartae																													0.110**	0.050	0.016	
l2diabe_l2heartae																													-0.017	0.052	-0.002	
l2smoken_l2heartae																													-0.230***	0.068	-0.028	
l2widowed_l2heartae																													-0.001	0.059	-0.000	
fstrok50_l2heartae																													0.040	0.198	0.006	
fcanc50_l2heartae																													0.128	0.110	0.020	
fdiabe50_l2heartae																													-0.014	0.081	-0.002	
fheart50_l2heartae																													0.389***	0.126	0.070	
fsmokev_l2heartae																													0.015	0.057	0.002	
fsmoken50_l2heartae																													-0.019	0.057	-0.003	
l2logbmi_l30_l2heartae																													-0.204	0.131	-0.029	
l2logbmi_30p_l2heartae																													0.889***	0.224	0.124	
logdeltaage_l2heartae																													-0.177	0.124	-0.025	
_cons	-3.918***	0.237			-3.689***	0.255			-3.703***	0.336			-3.932***	0.281			-4.487***	0.235			-8.931***	0.296			-2.705***	0.311			-1.325***	0.393		

note: .01 - ***; .05 - **; .1 - *;

	HI cov gov/emp/other (anyhi) coefficients		HI cov gov/emp/other (anyhi) marginal effects		Claiming SSDI (diclaim) coefficients		Claiming SSDI (diclaim) marginal effects		Claiming OASI (ssclaim) coefficients		Claiming OASI (ssclaim) marginal effects		Claiming DB (dbclaim) coefficients		Claiming DB (dbclaim) marginal effects		Claiming SSI (ssiclaim) coefficients		Claiming SSI (ssiclaim) marginal effects		R working for pay (work) coefficients		R working for pay (work) marginal effects	
	coef	se	coef	se	coef	se	coef	se	coef	se	coef	se	coef	se	coef	se	coef	se	coef	se	coef	se	coef	se
Non-Hispanic Black	-0.208***	0.024	-0.039		0.099***	0.036	0.006		-0.140***	0.032	-0.052		0.073***	0.017	0.021		0.204***	0.037	0.002		0.046**	0.019	0.017	
Hispanic	-0.566***	0.025	-0.125		-0.172***	0.049	-0.009		-0.165***	0.040	-0.061		-0.158***	0.024	-0.042		0.175***	0.046	0.002		0.008	0.024	0.003	
Less than high school	-0.261***	0.026	-0.051		-0.124***	0.042	-0.007		-0.067*	0.037	-0.025		-0.218***	0.018	-0.058		0.133***	0.038	0.001		-0.077***	0.022	-0.027	
Some college and above	0.268***	0.021	0.047		-0.039	0.034	-0.002		-0.287***	0.028	-0.108		0.139***	0.013	0.039		-0.076*	0.041	-0.001		0.065***	0.015	0.023	
Male	-0.044	0.028	-0.008		0.221***	0.043	0.014		-0.131***	0.035	-0.049		0.280***	0.016	0.080		-0.024	0.051	-0.000		0.120***	0.019	0.043	
Male AND Less than high school	-0.012	0.041	-0.002		0.124*	0.064	0.008		0.093*	0.055	0.035		0.036	0.026	0.010		-0.013	0.062	-0.000		0.040	0.032	0.014	
Male AND Non-Hispanic Black	-0.005	0.037	-0.001		-0.070	0.054	-0.004		0.080*	0.049	0.031		-0.150***	0.025	-0.040		-0.105*	0.060	-0.001		-0.095***	0.029	-0.033	
Male AND Hispanic	0.045	0.039	0.008		-0.055	0.074	-0.003		0.073	0.059	0.028		-0.093***	0.035	-0.025		-0.061	0.073	-0.000		0.018	0.035	0.006	
Male AND Some college and above	-0.106***	0.033	-0.019		-0.124**	0.052	-0.007		0.113***	0.041	0.043		-0.150***	0.020	-0.041		0.033	0.067	0.000		0.012	0.023	0.004	
Min(63, two-year lag of age)	0.006***	0.002	0.001										0.073***	0.002	0.021		-0.016***	0.004	-0.000					
Min(Max(0, two-year lag age - 63), 73 - 63)													-0.003	0.002	-0.001		0.012**	0.006	0.000					
Min(Max(0, two-year lag age - 73), 83 - 73)																								
Max(0, two-year lag age - 83)																								
Two-year lag of Heart disease	0.216***	0.027	0.033		0.112***	0.031	0.007		-0.047*	0.026	-0.018		0.007	0.011	0.002		0.047	0.030	0.000		-0.054***	0.015	-0.019	
Two-year lag of Stroke	-0.060	0.043	-0.011		0.089*	0.047	0.006		-0.066	0.041	-0.025		-0.047***	0.018	-0.013		-0.064	0.041	-0.000		-0.151***	0.028	-0.052	
Two-year lag of Cancer	0.234***	0.040	0.035		0.109**	0.049	0.007		-0.029	0.034	-0.011		0.015	0.014	0.004		0.064	0.042	0.001		-0.046**	0.019	-0.016	
Two-year lag of Hypertension	0.042***	0.015	0.007		0.134***	0.025	0.008		0.066***	0.019	0.025		0.029***	0.009	0.008		0.021	0.028	0.000		-0.093***	0.011	-0.033	
Two-year lag of Diabetes	0.076***	0.025	0.013		0.085**	0.034	0.005		-0.037	0.026	-0.014		0.011	0.012	0.003		-0.036	0.033	-0.000		-0.068***	0.016	-0.024	
Two-year lag of Lung disease	0.048	0.032	0.008		0.116***	0.037	0.008		-0.069*	0.035	-0.026		-0.038**	0.017	-0.011		0.116***	0.035	0.001		-0.126***	0.022	-0.044	
Two-year lag of R had heart attack since last wave																								
Two-year lag of Has exactly 1 IADL	0.062*	0.033	0.010		0.250***	0.037	0.019		-0.053	0.041	-0.020		-0.015	0.019	-0.004		0.094**	0.038	0.001		-0.203***	0.026	-0.069	
Two-year lag of Has 2 or more IADLs	0.255***	0.050	0.038		0.217***	0.047	0.016		0.001	0.053	0.000		0.003	0.024	0.001		0.063	0.044	0.001		-0.407***	0.046	-0.129	
Two-year lag of Has exactly 1 ADL	0.008	0.031	0.001		0.327***	0.037	0.026		-0.060	0.037	-0.022		-0.064***	0.017	-0.018		0.113***	0.037	0.001		-0.107***	0.023	-0.037	
Two-year lag of Has exactly 2 ADLs	0.048	0.046	0.008		0.323***	0.049	0.026		-0.021	0.054	-0.008		-0.077***	0.026	-0.021		0.111**	0.048	0.001		-0.294***	0.039	-0.096	
Two-year lag of Has 3 or more ADLs	0.027	0.047	0.005		0.462***	0.046	0.042		-0.112**	0.053	-0.042		-0.096***	0.026	-0.026		0.102**	0.045	0.001		-0.223***	0.041	-0.075	
Two-year lag of Current smoking																								
Two-year lag of Widowed	-0.108***	0.031	-0.020		0.061	0.046	0.004		0.147***	0.032	0.056		0.171***	0.013	0.050		-0.105***	0.037	-0.001		0.022	0.018	0.008	
Heart problem status at age 50 (1/0)-imputed	-0.100	0.072	-0.018		0.099	0.082	0.007		-0.112	0.078	-0.041		0.143***	0.041	0.042		0.013	0.083	0.000		0.000	0.052	0.000	
Stroke status at age 50 (1/0)-imputed	0.138*	0.082	0.022		-0.011	0.090	-0.001		-0.139	0.138	-0.051		-0.109	0.079	-0.029		0.131	0.093	0.001		0.103	0.076	0.037	
Cancer status at age 50 (1/0)-imputed	-0.107**	0.047	-0.020		0.005	0.064	0.000		-0.009	0.056	-0.003		-0.036	0.030	-0.010		-0.053	0.069	-0.000		0.021	0.032	0.007	
Diabetes status at age 50 (imputed)	-0.000	0.031	-0.000		0.060	0.042	0.004		-0.003	0.041	-0.001		-0.022	0.023	-0.006		0.052	0.045	0.000		-0.043*	0.025	-0.015	
Init. of Ever smoked	0.043**	0.018	0.007		0.068**	0.030	0.004		0.053**	0.022	0.020		0.014	0.011	0.004		0.030	0.035	0.000		-0.015	0.013	-0.005	
Smoking status at age 50 (imputed)	-0.197***	0.019	-0.036		0.090***	0.030	0.006		0.027	0.024	0.010		-0.021*	0.012	-0.006		0.086***	0.033	0.001		-0.101***	0.014	-0.036	
Max(0, two-year lag age - 73)													-0.004***	0.001	-0.001		-0.017***	0.005	-0.000					
Log of years between current interview and previous	0.173***	0.035	0.030		0.145**	0.059	0.009		0.550***	0.056	0.207		0.040	0.026	0.011		-0.161**	0.063	-0.001		-0.127***	0.029	-0.045	
Two-year lag of R working for pay	-0.224***	0.022	-0.037		-0.551***	0.037	-0.040		-0.208***	0.031	-0.078						-0.265***	0.055	-0.002		1.688***	0.014	0.565	
Two-year lag of (IHT of earnings in 1000s)/100, zero otherwise	9.417***	0.523	1.629		-0.592	0.892	-0.035		-3.143***	0.712	-1.185		-1.339***	0.281	-0.377		-6.687***	1.465	-0.053		14.534***	0.353	5.161	
Two-year lag of Non-pension wlth(hatotb) not zero	-0.093***	0.032	-0.015		0.121***	0.045	0.006		0.170***	0.048	0.062		0.291***	0.032	0.072		-0.144***	0.038	-0.001		0.220***	0.034	0.074	
Two-year lag of (IHT of hh wlth in 1000s)/100, zero otherwise	6.843***	0.244	1.184		-2.319***	0.391	-0.139		0.094	0.390	0.035		2.101***	0.211	0.592		-4.762***	0.433	-0.038		-1.386***	0.219	-0.492	
Two-year lag of Claiming SSDI	1.275***	0.047	0.110		2.703***	0.029	0.687		-1.228***	0.036	-0.346		-0.086***	0.027	-0.023		0.661***	0.035	0.013		-0.382***	0.032	-0.122	
Two-year lag of Claiming OASI - Reports receiving SS retirement income and 62+	0.109**	0.043	0.018										-0.018	0.016	-0.005		-0.171***	0.045	-0.001		0.074***	0.018	0.026	
nraplus10					0.317***	0.053	0.022																	
nraplus9					0.497***	0.063	0.045																	
nraplus8					0.475***	0.062	0.042																	
nraplus7					0.382***	0.063	0.031																	
nraplus6					0.472***	0.061	0.042																	
nraplus5					0.438***	0.061	0.038																	
nraplus4					0.470***	0.060	0.041																	
nraplus3					0.582***	0.058	0.056																	
nraplus2					0.518***	0.058	0.047																	
nraplus1					0.383***	0.059	0.031																	
Two-year lag of Claiming DB									0.022	0.024	0.008		1.959***	0.010	0.642		-0.265***	0.041	-0.002		-0.058***	0.013	-0.021	
Respondent is at the EEA age (62) or up to two years older									0.781***	0.033	0.294										-0.154***	0.019	-0.053	
Respondent is at their NRA age or two years older									0.767***	0.029	0.298										-0.080***	0.020	-0.028	
Years to NRA for those not year at NRA									-0.319***	0.014	-0.120										0.051***	0.002	0.018	
Years past NRA for those older than NRA									-0.096***	0.003	-0.036										-0.031***	0.002	-0.011	
Two-year lag of Claiming SSI																	2.971***	0.029	0.538					
Seasonally Adjusted Unemployment Rate																					-0.006*	0.003	-0.002	
_cons	0.398***	0.111			-2.560***	0.086			-0.196***	0.071			-6.235***	0.130			-1.188***	0.232			-1.448***	0.047		

note: .01 - ***; .05 - **; .1 - *;

	Medicaid eligibility MEPS coefficients		Medicaid eligibility MEPS marginal effects	
	coef	se	coef	se
male	-0.146***	0.055	-0.007	
male_black	0.197**	0.089	0.011	
male_hispan	0.231**	0.097	0.013	
male_hsless	0.052	0.083	0.002	
R is non-hispanic black	0.235***	0.055	0.013	
HISPANIC ETHNICITY (EDITED/IMPUTED)	0.198***	0.061	0.011	
Less than HS	0.371***	0.056	0.023	
At least some college	-0.309***	0.045	-0.015	
Marital status:widowed	0.757***	0.068	0.069	
Marital status: single	0.914***	0.042	0.066	
Cancer (except skin) CCC code 11-21/24-45	0.081	0.073	0.004	
logiearnx	-10.086***	0.358	-0.456	
One or more ADLs	0.906***	0.085	0.098	
_cons	-1.417***	0.046		
Number of observations	19,839		19,839	
Pseudo R2	0.312		0.312	

note: .01 - ***; .05 - **; .1 - *;

	Medicaid eligibility MCBS coefficients		Medicaid eligibility MCBS marginal effects	
	coef	se	coef	se
Male	-0.172***	0.024	-0.030	
Black	0.547***	0.032	0.125	
Hispanic	0.670***	0.033	0.161	
Less than high school	0.660***	0.023	0.143	
male_black	-0.072	0.050	-0.012	
male_hispan	0.106**	0.051	0.020	
male_hsless	0.019	0.035	0.003	
Some college and above	-0.247***	0.021	-0.042	
widowed	0.358***	0.020	0.069	
single	0.909***	0.021	0.220	
Cancer	-0.069***	0.021	-0.012	
Living in nursing home	1.556***	0.031	0.499	
ADL 3+-Not in nursing home	0.541***	0.024	0.123	
_cons	-1.716***	0.021		
Number of observations	59,010		59,010	
Pseudo R2	0.244		0.244	

note: .01 - ***; .05 - **; .1 - *;

	Part B initial takeup coefficients		Pt B initial takeup marginal effects	
	coef	se	coef	se
Male	-0.226***	0.028	-0.016	
non-hispanic black	-0.143***	0.044	-0.011	
hispanic origin	-0.225***	0.042	-0.019	
less than high school diploma	0.013	0.037	0.001	
some college and above	-0.082***	0.031	-0.006	
widowed	0.116***	0.038	0.008	
age: (.,65)	-0.003	0.009	-0.000	
age: (65,75)	0.100***	0.005	0.007	
age: (75,.)	-0.000	0.004	-0.000	
iearnx	-0.003***	0.000	-0.000	
Working	-0.722***	0.029	-0.083	
SR cancer (excl. skin)	-0.019	0.033	-0.001	
SR hypertension	0.079***	0.029	0.006	
SR diabetes	-0.048	0.032	-0.003	
SR heart disease	0.090*	0.047	0.006	
SR stroke	-0.102	0.093	-0.008	
adl3p	0.027	0.055	0.002	
obese: bmi >= 30kg/m2	0.062**	0.030	0.004	
ever smoked	-0.069**	0.033	-0.005	
diclaim	0.123*	0.070	0.008	
hibpe_stroke	0.210*	0.108	0.013	
hearte_smokev	0.048	0.057	0.003	
_cons	1.718***	0.558		
Number of observations	54,400		54,400	
Pseudo R2	0.184		0.184	

note: .01 - ***; .05 - **; .1 - *;

	New enrollee Part B Take up coefficients		New enrollee Part B Take up marginal effects		Current enrollee Part B Take up coefficients		Current enrollee Part B Take up marginal effects		New enrollee Part A Take up coefficients		New enrollee Part A Take up marginal effects		Current enrollee Part A Take up coefficients		Current enrollee Part A Take up marginal effects	
	coef	se	coef	se	coef	se	coef	se	coef	se	coef	se	coef	se	coef	se
Male	-0.108	0.083	-0.030		-0.136	0.085	-0.032		-0.108	0.083	-0.030		-0.136	0.085	-0.032	
non-hispanic black	-0.070	0.140	-0.020		-0.044	0.122	-0.010		-0.070	0.140	-0.020		-0.044	0.122	-0.010	
hispanic origin	-0.007	0.155	-0.002		-0.132	0.136	-0.029		-0.007	0.155	-0.002		-0.132	0.136	-0.029	
less than high school diploma	-0.028	0.135	-0.008		0.151	0.124	0.037		-0.028	0.135	-0.008		0.151	0.124	0.037	
some college and above	-0.127	0.089	-0.036		-0.001	0.092	-0.000		-0.127	0.089	-0.036		-0.001	0.092	-0.000	
widowed	0.240	0.149	0.062		-0.082	0.137	-0.019		0.240	0.149	0.062		-0.082	0.137	-0.019	
iearnx	-0.005***	0.001	-0.001		-0.001	0.001	-0.000		-0.005***	0.001	-0.001		-0.001	0.001	-0.000	
Working	-0.760***	0.085	-0.239		-0.646***	0.089	-0.143		-0.760***	0.085	-0.239		-0.646***	0.089	-0.143	
SR cancer (excl. skin)	0.067	0.112	0.019		-0.030	0.103	-0.007		0.067	0.112	0.019		-0.030	0.103	-0.007	
SR hypertension	0.017	0.104	0.005		0.111	0.086	0.026		0.017	0.104	0.005		0.111	0.086	0.026	
SR heart disease	0.088	0.113	0.024		0.112	0.156	0.027		0.088	0.113	0.024		0.112	0.156	0.027	
SR stroke	0.071	0.190	0.019		0.359	0.237	0.097		0.071	0.190	0.019		0.359	0.237	0.097	
iadl1	0.185	0.143	0.049						0.185	0.143	0.049					
iadl2p	0.243	0.199	0.063						0.243	0.199	0.063					
adl1	0.028	0.151	0.008						0.028	0.151	0.008					
adl2	0.088	0.220	0.024						0.088	0.220	0.024					
adl3p	-0.219	0.276	-0.067		0.122	0.159	0.030		-0.219	0.276	-0.067		0.122	0.159	0.030	
obese: bmi >= 30kg/m2	0.058	0.156	0.016		0.139	0.086	0.033		0.058	0.156	0.016		0.139	0.086	0.033	
ever smoked	-0.072	0.082	-0.020		-0.090	0.099	-0.021		-0.072	0.082	-0.020		-0.090	0.099	-0.021	
diclaim	-0.112	0.173	-0.033		-1.381***	0.223	-0.196		-0.112	0.173	-0.033		-1.381***	0.223	-0.196	
diabe_hibpe	0.021	0.137	0.006						0.021	0.137	0.006					
diabe_hearte	-0.269	0.193	-0.082		-0.003	0.189	-0.001		-0.269	0.193	-0.082		-0.003	0.189	-0.001	
hibpe_obese	-0.066	0.191	-0.019						-0.066	0.191	-0.019					
diclaim_adl3p	0.426	0.389	0.100						0.426	0.389	0.100					
age75l					-0.071***	0.014	-0.016						-0.071***	0.014	-0.016	
age75p					-0.050**	0.021	-0.012						-0.050**	0.021	-0.012	
SR diabetes					-0.033	0.110	-0.008						-0.033	0.110	-0.008	
SR emphysema,asthma,COPD					-0.013	0.108	-0.003						-0.013	0.108	-0.003	
hibpe_stroke					-0.463	0.293	-0.086						-0.463	0.293	-0.086	
hearte_smokev					-0.049	0.186	-0.011						-0.049	0.186	-0.011	
_cons	1.317***	0.123			4.297***	0.995			1.317***	0.123			4.297***	0.995		
Number of observations	1,527		1,527		2,102		2,102		1,527		1,527		2,102		2,102	
Pseudo R2	0.104		0.104		0.085		0.085		0.104		0.104		0.085		0.085	

note: .01 - ***; .05 - **; .1 - *;

	Part D enrollment coefficients		Part D enrollment marginal effects		Part D capitated payment coefficients		Part D capitated payment marginal effects	
	coef	se	coef	se	coef	se	coef	se
age75l	0.029***	0.002	0.012		-11.594***	2.706	-11.594	
age75p	-0.023***	0.001	-0.009		-7.863***	1.838	-7.863	
Male	-0.026*	0.014	-0.010		-203.609***	15.214	-203.609	
non-hispanic black	0.099***	0.022	0.039		485.824***	28.913	485.824	
hispanic origin	0.259***	0.024	0.101		133.498***	32.168	133.498	
less than high school diploma	0.095***	0.016	0.038		503.761***	20.031	503.761	
some college and above	-0.051***	0.015	-0.020		-112.022***	15.542	-112.022	
married	0.033**	0.014	0.013					
iearnx	-0.004***	0.000	-0.002					
Working	-0.114***	0.020	-0.045					
SR cancer (excl. skin)	-0.001	0.015	-0.000					
SR diabetes	0.021	0.014	0.008		211.022***	17.546	211.022	
SR hypertension	0.039***	0.014	0.015		146.329***	16.294	146.329	
SR stroke	0.060***	0.019	0.024		101.539***	23.702	101.539	
SR heart disease	0.047***	0.013	0.019		168.093***	15.515	168.093	
adl3p	0.017	0.021	0.007		202.536***	38.062	202.536	
ever smoked	-0.019	0.013	-0.007					
diclaim	0.519***	0.038	0.197		719.101***	47.440	719.101	
widowed					143.054***	17.254	143.054	
SR emphysema,asthma,COPD					212.392***	19.886	212.392	
R smokes now					163.776***	25.027	163.776	
iadl1					173.015***	20.726	173.015	
iadl2p					311.437***	27.081	311.437	
adl1					62.444***	22.178	62.444	
adl2					79.481**	33.860	79.481	
_cons	-1.875***	0.158			1,590.954***	192.251		
Number of observations	54,814		54,814		29,885		29,885	
Adjusted/Pseudo R2	0.028		0.028		0.253		0.253	

note: .01 - ***; .05 - **; .1 - *;

	Smoking status (smkstat) coefficients		Smoking status (smkstat) marginal effects					
				_				
	coef	se	coef	se	coef	se	coef	se
Non-Hispanic Black	-0.020	0.013	0.006		-0.006		-0.000	
Hispanic	-0.169***	0.016	0.056		-0.053		-0.003	
Less than high school	0.005	0.013	-0.001		0.001		0.000	
Some college and above	0.092***	0.010	-0.029		0.027		0.002	
Male	0.524***	0.013	-0.159		0.148		0.011	
Male AND Less than high school	0.028	0.021	-0.009		0.008		0.001	
Male AND Non-Hispanic Black	-0.135***	0.021	0.044		-0.042		-0.002	
Male AND Hispanic	0.139***	0.025	-0.042		0.039		0.003	
Male AND Some college and above	-0.218***	0.016	0.072		-0.069		-0.003	
Min(63, two-year lag of age)	-0.000	0.001	0.000		-0.000		-0.000	
Min(Max(0, two-year lag age - 63), 73 - 63)	-0.008***	0.001	0.003		-0.002		-0.000	
Max(0, two-year lag age - 73)	-0.014***	0.001	0.004		-0.004		-0.000	
Two-year lag of Heart disease	0.071***	0.010	-0.022		0.021		0.001	
Two-year lag of Stroke	0.052***	0.015	-0.016		0.015		0.001	
Two-year lag of Cancer	0.064***	0.012	-0.020		0.019		0.001	
Two-year lag of Hypertension	-0.000	0.008	0.000		-0.000		-0.000	
Two-year lag of Diabetes	-0.014	0.011	0.004		-0.004		-0.000	
Two-year lag of Lung disease	0.191***	0.014	-0.057		0.053		0.004	
Two-year lag of R had heart attack since last wave	0.097***	0.028	-0.030		0.028		0.002	
Two-year lag of Has exactly 1 IADL	0.049***	0.015	-0.015		0.014		0.001	
Two-year lag of Has 2 or more IADLs	0.009	0.019	-0.003		0.003		0.000	
Two-year lag of Has exactly 1 ADL	0.035**	0.014	-0.011		0.010		0.001	
Two-year lag of Has exactly 2 ADLs	0.034	0.021	-0.011		0.010		0.001	
Two-year lag of Has 3 or more ADLs	-0.021	0.020	0.007		-0.006		-0.000	
Two-year lag of Current smoking	2.604***	0.016	-0.375		-0.017		0.392	
Two-year lag of Widowed	-0.036***	0.011	0.012		-0.011		-0.001	
Heart problem status at age 50 (1/0)-imputed	0.079**	0.034	-0.024		0.023		0.002	
Stroke status at age 50 (1/0)-imputed	-0.279***	0.051	0.095		-0.092		-0.003	
Cancer status at age 50 (1/0)-imputed	0.018	0.022	-0.006		0.005		0.000	
Diabetes status at age 50 (imputed)	0.007	0.017	-0.002		0.002		0.000	
Smoking status at age 50 (imputed)	1.959***	0.013	-0.441		0.313		0.128	
Splined two-year lag of BMI <= log(30)	-0.080*	0.045	0.025		-0.024		-0.001	
Splined two-year lag of BMI > log(30)	0.327***	0.059	-0.103		0.097		0.006	
Splined init of BMI age 50 <= log(30)	-0.006	0.045	0.002		-0.002		-0.000	
Splined init of BMI age 50 > log(30)	-0.247***	0.063	0.078		-0.074		-0.004	
Log of years between current interview and previous	-0.003	0.020	0.001		-0.001		-0.000	

note: .01 - ***; .05 - **; .1 - *;

	Log(BMI) (logbmi) coefficients		Log(BMI) (logbmi) marginal effects	
	coef	se	coef	se
Age in years at July 1st				
agesq				
Male	0.000	0.001	0.000	
Married				
Non-Hispanic Black	-0.002**	0.001	-0.002	
Hispanic	-0.002**	0.001	-0.002	
Number of children, based on 2004 report				
Less than high school	-0.002***	0.001	-0.002	
Some college and above	-0.000	0.001	-0.000	
Male AND Less than high school	0.001	0.001	0.001	
Male AND Non-Hispanic Black	-0.005***	0.001	-0.005	
Male AND Hispanic	-0.000	0.001	-0.000	
Male AND Some college and above	-0.001	0.001	-0.001	
Min(63, two-year lag of age)	-0.000**	0.000	-0.000	
Min(Max(0, two-year lag age - 63), 73 - 63)	-0.001***	0.000	-0.001	
Max(0, two-year lag age - 73)	-0.002***	0.000	-0.002	
Two-year lag of Heart disease	-0.000	0.001	-0.000	
Two-year lag of Stroke	-0.002***	0.001	-0.002	
Two-year lag of Cancer	-0.001	0.001	-0.001	
Two-year lag of Hypertension	0.004***	0.000	0.004	
Two-year lag of Diabetes	-0.001	0.001	-0.001	
Two-year lag of Lung disease	-0.005***	0.001	-0.005	
Two-year lag of R had heart attack since last wave	0.005***	0.002	0.005	
Two-year lag of Has exactly 1 IADL	-0.001	0.001	-0.001	
Two-year lag of Has 2 or more IADLs	-0.005***	0.001	-0.005	
Two-year lag of Has exactly 1 ADL	0.001	0.001	0.001	
Two-year lag of Has exactly 2 ADLs	0.000	0.001	0.000	
Two-year lag of Has 3 or more ADLs	0.001	0.001	0.001	
Two-year lag of Current smoking	-0.012***	0.001	-0.012	
Two-year lag of Widowed	0.001	0.001	0.001	
Heart problem status at age 50 (1/0)-imputed	0.001	0.002	0.001	
Stroke status at age 50 (1/0)-imputed	-0.005	0.003	-0.005	
Cancer status at age 50 (1/0)-imputed	0.002	0.001	0.002	
Diabetes status at age 50 (imputed)	-0.004***	0.001	-0.004	
Init. of Ever smoked	0.001**	0.001	0.001	
Smoking status at age 50 (imputed)	0.002***	0.001	0.002	
Splined two-year lag of BMI <= log(30)	0.813***	0.003	0.813	
Splined two-year lag of BMI > log(30)	0.835***	0.004	0.835	
Splined init of BMI age 50 <= log(30)	0.139***	0.003	0.139	
Splined init of BMI age 50 > log(30)	0.099***	0.004	0.099	
Log of years between current interview and previous	-0.010***	0.001	-0.010	
Init. of	0.000	0.000	0.000	
_cons	0.112	0.076		

note: .01 - ***; .05 - **; .1 - *;

	Earnings (iearn) coefficients		Uncapped earnings (iearnuc) coefficients		Household wealth (hatotb) coefficients	
	coef	se	coef	se	coef	se
Non-Hispanic Black	-0.014	0.056	-0.062	0.048	-5.250***	0.292
Hispanic	-0.600***	0.073	-0.464***	0.063	-3.282***	0.375
Less than high school	-0.428***	0.075	-0.284***	0.064	-2.137***	0.264
Some college and above	0.893***	0.046	0.618***	0.040	4.953***	0.214
Male	1.168***	0.058	0.799***	0.050	0.833***	0.259
Male AND Less than high school	-0.015	0.105	0.021	0.090	-0.558	0.415
Male AND Non-Hispanic Black	-0.454***	0.086	-0.315***	0.074	-0.201	0.468
Male AND Hispanic	-0.347***	0.103	-0.281***	0.089	-1.997***	0.584
Male AND Some college and above	0.056	0.067	0.118**	0.058	0.271	0.333
Min(63, two-year lag of age)	-0.061***	0.004	-0.052***	0.004		
Min(Max(0, two-year lag age - 63), 73 - 63)	-0.045***	0.009	-0.037***	0.008		
Max(0, two-year lag age - 73)	-0.097***	0.014	-0.061***	0.012		
Two-year lag of Heart disease	-0.134***	0.051	-0.104**	0.044	-0.903***	0.188
Two-year lag of Stroke	-0.391***	0.104	-0.341***	0.090	-1.228***	0.290
Two-year lag of Cancer	0.085	0.065	0.050	0.056	1.018***	0.223
Two-year lag of Hypertension	-0.017	0.032	-0.026	0.028	-0.953***	0.155
Two-year lag of Diabetes	0.002	0.052	-0.035	0.045	-1.596***	0.208
Two-year lag of Lung disease	-0.180**	0.079	-0.181***	0.068	-1.935***	0.269
Two-year lag of Has exactly 1 IADL	-0.481***	0.096	-0.388***	0.082	-1.606***	0.316
Two-year lag of Has 2 or more IADLs	-0.288	0.220	-0.154	0.189	-1.247***	0.396
Two-year lag of Has exactly 1 ADL	-0.191**	0.080	-0.121*	0.068	-1.373***	0.279
Two-year lag of Has exactly 2 ADLs	-0.461***	0.148	-0.360***	0.128	-1.449***	0.429
Two-year lag of Has 3 or more ADLs	-0.516***	0.178	-0.409***	0.153	-1.944***	0.425
Two-year lag of Widowed	0.285***	0.061	0.286***	0.053	-3.143***	0.203
Two-year lag of R working for pay	0.343***	0.061	0.323***	0.052	-1.455***	0.273
Two-year lag of (IHT of earnings in 1000s)/100, zero otherwise	125.999***	1.121				
Two-year lag of Non-pension wlth(hatotb) not zero	0.668***	0.127	0.459***	0.109		
Two-year lag of (IHT of hh wlth in 1000s)/100, zero otherwise	8.946***	0.628	6.758***	0.541	892.482***	3.602
Two-year lag of Claiming SSDI	-1.029***	0.174	-0.619***	0.149		
Two-year lag of Claiming OASI - Reports receiving SS retirement income and 62+	-1.519***	0.064	-1.291***	0.055		
Two-year lag of Claiming DB	-0.688***	0.047	-0.659***	0.041		
Heart problem status at age 50 (1/0)-imputed	-0.550***	0.175	-0.457***	0.150	-1.387**	0.636
Stroke status at age 50 (1/0)-imputed	0.237	0.240	0.239	0.206	-1.093	1.257
Cancer status at age 50 (1/0)-imputed	0.066	0.099	0.077	0.085	-0.354	0.492
Diabetes status at age 50 (imputed)	-0.101	0.076	-0.033	0.066	-0.485	0.405
Init. of Ever smoked	-0.008	0.036	-0.025	0.031	-0.122	0.182
Smoking status at age 50 (imputed)	-0.192***	0.041	-0.120***	0.036	-2.033***	0.194
Log of years between current interview and previous	-0.310***	0.079	-0.249***	0.068	-2.134***	0.467
Two-year lag of (IHT of earnings in 1000s)/100, zero otherwise			97.208***	1.025	38.009***	6.810
Init. of (IHT of earnings in 1000s)/100, zero otherwise			29.016***	0.971		
l2age_iwe: (.,58)					0.200***	0.056
l2age_iwe: (58,73)					0.057***	0.020
l2age_iwe: (73,.)					-0.110***	0.020
Two-year lag of R live in nursingh ome at interview					-1.684**	0.760
w5					-0.148	0.230
w6					0.098	0.236
w7					0.548**	0.236
w8					1.289***	0.241
_cons	5.683***	0.277	4.481***	0.238	-5.549*	3.175

note: .01 - ***; .05 - **; .1 - *;

	Total med costs MEPS (totmd_meps) coefficients		Total med costs MEPS (totmd_meps) marginal effects	
	coef	se	coef	se
age5559	464.534**	228.032	464.534	
age6064	499.205*	258.643	499.205	
age6569	-220.122	281.469	-220.122	
male	-460.362**	211.987	-460.362	
male_black	750.697	507.308	750.697	
male_hispan	144.067	447.840	144.067	
male_hsless	562.276	520.210	562.276	
R is non-hispanic black	-609.062**	301.161	-609.062	
HISPANIC ETHNICITY (EDITED/IMPUTED)	-679.560**	299.099	-679.560	
Less than HS	-1,237.731***	415.802	-1,237.731	
At least some college	627.250***	210.411	627.250	
Marital status:widowed	-200.180	363.860	-200.180	
Marital status: single	-341.781	208.744	-341.781	
Cancer (except skin) CCC code 11-21/24-45	7,296.490***	729.208	7,296.490	
Ever diagnosed with diabetes	2,096.541***	297.753	2,096.541	
Ever diagnosed with high blood pressure	800.862***	204.490	800.862	
CHD/ANGINA/MI/OTHER heart problems	2,322.464***	290.278	2,322.464	
Ever diagnosed with emphysema	2,034.288***	494.776	2,034.288	
Ever diagnosed with stroke	3,693.040***	767.454	3,693.040	
MI CCC code 100	5,112.582***	1,201.361	5,112.582	
logiearnx	-6,290.732***	2,196.628	-6,290.732	
2 or more PFLs	5,852.323***	380.686	5,852.323	
_cons	2,604.867***	341.168		
Number of observations	24,370		24,370	
Adjusted R2	0.125		0.125	

note: .01 - ***; .05 - **; .1 - *;

	Total med costs MCBS (totmd_mcbs) coefficients		Total med costs MCBS (totmd_mcbs) marginal effects	
	coef	se	coef	se
age6569	622.721	708.717	622.721	
age7074	1,150.926	715.291	1,150.926	
age7579	2,579.613***	738.613	2,579.613	
age8084	2,765.673***	746.934	2,765.673	
age85	1,860.328**	765.709	1,860.328	
Male	-298.499	213.371	-298.499	
male_black	1,955.215**	950.143	1,955.215	
male_hispan	489.000	707.856	489.000	
male_hsless	372.663	486.776	372.663	
Black	736.949	543.757	736.949	
Hispanic	-486.474	443.965	-486.474	
Less than high school	-1,188.173***	317.422	-1,188.173	
Some college and above	326.040	203.861	326.040	
widowed	-67.411	247.930	-67.411	
single	-240.552	260.984	-240.552	
Diagnosis stage for Cancer	15,432.690***	1,158.708	15,432.690	
Diagnosis stage for Diabetes	2,850.537***	847.552	2,850.537	
Diagnosis stage for Hypertension	3,513.701***	589.926	3,513.701	
Diagnosis stage for Heart disease	6,661.840***	771.338	6,661.840	
Diagnosis stage for Lung disease	5,445.194***	802.966	5,445.194	
Diagnosis stage for Stroke	8,049.540***	1,348.074	8,049.540	
Diagnosis stage for Heart attack	11,181.253***	1,896.935	11,181.253	
Maintenance stage for Cancer	2,153.415***	256.729	2,153.415	
Maintenance stage for Diabetes	1,838.109***	521.885	1,838.109	
Maintenance stage for Heart disease	2,298.899***	423.225	2,298.899	
Maintenance stage for Hypertension	1,235.764***	213.179	1,235.764	
Maintenance stage for Lung disease	2,073.376***	268.627	2,073.376	
Maintenance stage for Stroke	435.191	706.017	435.191	
Maintenance stage for Heart attack	1,536.663***	384.137	1,536.663	
Living in nursing home	46,275.881***	940.248	46,275.881	
ADL 1 - Not in nursing home	2,478.771***	309.764	2,478.771	
ADL 2 - Not in nursing home	3,377.968***	436.596	3,377.968	
ADL 3+-Not in nursing home	5,938.621***	526.661	5,938.621	
IADL 1-Not in nursing home	1,879.333***	364.829	1,879.333	
IADL 2+-Not in nursing home	5,926.576***	500.121	5,926.576	
Eligable for Medicare due to disablity	1,455.900*	775.788	1,455.900	
Died	-1,241.608	6,061.244	-1,241.608	
diabe_hearte	1,205.183**	507.844	1,205.183	
diabe_hibpe	521.370	598.938	521.370	
hibpe_hearte	-63.150	473.341	-63.150	
hibpe_stroke	1,338.382	814.699	1,338.382	
diclaim_died	25,284.524***	7,988.619	25,284.524	
diclaim_nhmliv	-10,958.177***	3,111.035	-10,958.177	
died_nhmliv	-29,427.244***	2,425.311	-29,427.244	
Terminal stage for Cancer	2,309.145	2,059.570	2,309.145	
Terminal stage for Diabetes	5,098.450**	2,294.892	5,098.450	
Terminal stage for Hypertension	-1,257.664	2,108.378	-1,257.664	
Terminal stage for Heart disease	5,395.001**	2,296.867	5,395.001	
Terminal stage for Lung disease	-4,448.423**	2,016.236	-4,448.423	
Terminal stage for Stroke	-650.581	2,217.521	-650.581	
Terminal stage for Heart attack	-2,389.863	2,650.831	-2,389.863	
died_age6569	23,641.484***	6,992.854	23,641.484	
died_age7074	27,790.923***	6,846.184	27,790.923	
died_age7579	18,612.132***	6,532.815	18,612.132	
died_age8084	17,267.832***	5,737.284	17,267.832	
died_age85	12,456.736**	5,405.532	12,456.736	
nhmliv_heartae	-590.972	3,126.156	-590.972	
o.died_heartae	(dropped)			
_cons	3,737.433***	696.022		
Number of observations	40,147		40,147	
Adjusted R2	0.229		0.229	

note: .01 - ***; .05 - **; .1 - *;
