# Supplementary material for: Lifetime burden of prescription medication for insomnia in middle-aged and older adults in the US: a microsimulation study
Source: Lancet Reg Health Am. 2025 Oct 24;52:101284. doi: 10.1016/j.lana.2025.101284 (PMC12593590; doi:10.1016/j.lana.2025.101284)
Supplement: Supplementary data 3 [file mmc3.pdf]

The Future Elderly Model: Technical Documentation

Dana P. Goldman, University of Southern California
Hanke Heun-Johnson, University of Southern California
Duncan Ermini Leaf, University of Southern California
Jeffrey Sullivan, Precision Health Economics, LLC
Bryan Tysinger, University of Southern California

July 14, 2025

Contents

1 Functioning of the dynamic model **4**
1.1 Background 4
1.2 Overview 5
1.3 Comparison with other prominent microsimulation models of health expenditures . 6
1.3.1 CBOLT Model 6
1.3.2 Centers for Medicare and Medicaid Services 6

2 Data sources used for estimation **7**
2.1 Health and Retirement Study 7
2.2 Social Security covered earnings files 8
2.3 Medical Expenditure Panel Survey 8
2.4 Medicare Current Beneficiary Survey 8

3 Data sources for trends and baseline scenario **9**
3.1 Data for growth in wages 9
3.2 Demographic adjustments 9

4 Estimation **9**
4.1 Transition model 9
4.1.1 Inverse Hyperbolic Sine Transformation 11
4.2 Health Utility Index Mark 3 12

5 Medical costs **12**
5.1 Medical costs estimation 12

6 Implementation **12**
6.1 Intervention Module 13

7 Model development **14**
7.1 Health Utility Index Mark 3 14
7.1.1 Data description 14
7.1.2 Missing data: Logical limits 14
7.1.3 Missing data: Conditionally-almost-sure imputations 15

8 Validation **15**
8.1 Cross-validation 15
8.1.1 Mortality and Nursing Home Status 15
8.1.2 Demographics 16
8.1.3 Health Outcomes 16
8.1.4 Health Risk Factors 16
8.1.5 Economic Outcomes 16
8.2 External Corroboration 16

9 Baseline Forecasts **16**
9.1 Disease Prevalence 16

10 Acknowledgments **19**

11 Tables 19

References 31

List of Figures

1	Architecture of the FEM	6
2	Historic and Forecasted Chronic Disease Prevalence for Men 55+	17
3	Historic and Forecasted Chronic Disease Prevalence for Women 55+	17
4	Historic and Forecasted ADL and IADL Prevalence for Men 55+	18
5	Historic and Forecasted ADL and IADL Prevalence for Women 55+	18

List of Tables

1	Outcomes in the transition model	20
2	Restrictions on transition model	21
3	Desciptive statistics for exogeneous control variables	22
4	Health condition prevalences in survey data	23
5	Survey questions used to determine health conditions	24
6	Per capita medical spending by payment source, age group, and year	25
7	Assumptions for each calendar year	26
8	Assumptions for each birth year	27
9	OLS regression of the predicted HUI3 score against chronic conditions and FEM-type functional status specification	28
10	Crossvalidation of 1998 cohort: Simulated vs reported mortality and nursing home outcomes in 2000, 2006, 2012, and 2018	29
11	Crossvalidation of 1998 cohort: Simulated vs reported demographic outcomes in 2000, 2006, 2012, and 2018	29
12	Crossvalidation of 1998 cohort: Simulated vs reported binary health outcomes in 2000, 2006, 2012, and 2018	29
13	Crossvalidation of 1998 cohort: Simulated vs reported risk factor outcomes in 2000, 2006, 2012, and 2018	30
14	Crossvalidation of 1998 cohort: Simulated vs reported binary economic outcomes in 2000, 2006, 2012, and 2018	30
15	Crossvalidation of 1998 cohort: Simulated vs reported continuous economic outcomes in 2000, 2006, 2012, and 2018	30

This appendix describes technical details to support the paper **"Lifetime Burden of Prescription Medication for Insomnia in Middle-Aged and Older Adults in the U.S.: A Microsimulation Study"**.

1 Functioning of the dynamic model

1.1 Background

The Future Elderly Model (FEM) is a microsimulation model originally developed out of an effort to examine health and health care costs among the elderly Medicare population (age 65+). A description of the previous incarnation of the model can be found in Goldman et al. (2004). The

original work was founded by the Centers for Medicare and Medicaid Services and carried out by a team of researchers composed of Dana P. Goldman, Paul G. Shekelle, Jayanta Bhattacharya, Michael Hurd, Geoffrey F. Joyce, Darius N. Lakdawalla, Dawn H. Matsui, Sydne J. Newberry, Constantijn W. A. Panis and Baoping Shang.

Since then various extensions have been implemented to the original model. The most recent version now projects health outcomes for all Americans aged 51 and older and uses the Health and Retirement Study (HRS) as a host dataset rather than the Medicare Current Beneficiary Survey (MCBS). The work has also been extended to include economic outcomes such as earnings, labor force participation and pensions. This work was funded by the National Institute on Aging through its support of the RAND Roybal Center for Health Policy Simulation (P30AG024968), the Department of Labor through contract J-9-P-2-0033, the National Institutes of Aging through the R01 grant “Integrated Retirement Modeling” (R01AG030824) and the MacArthur Foundation Research Network on an Aging Society. Finally, the computer code of the model was transferred from Stata to C++. This report incorporates these new development efforts in the description of the model.

1.2 Overview

The defining characteristic of the model is the modeling of real rather than synthetic cohorts, all of whom are followed at the individual level. This allows for more heterogeneity in behavior than would be allowed by a cell-based approach. Also, since the HRS interviews both respondent and spouse, we can link records to calculate household-level outcomes such as net income and Social Security retirement benefits, which depend on the outcomes of both spouses. The omission of the population younger than age 51 sacrifices little generality, since the bulk of expenditure on the public programs we consider occurs after age 50. However, we may fail to capture behavioral responses among the young.

The model has three core components:

- The initial cohort module predicts the economic and health outcomes of new cohorts of 51/52 year-olds. This module takes in data from the Health and Retirement Study (HRS) and trends calculated from other sources. It allows us to “generate” cohorts as the simulation proceeds, so that we can measure outcomes for the age 51+ population in any given year. In this study, no new cohorts are added.

- The transition module calculates the probabilities of transiting across various health states and financial outcomes. The module takes as inputs risk factors such as smoking, weight, age and education, along with lagged health and financial states. This allows for a great deal of heterogeneity and fairly general feedback effects. The transition probabilities are estimated from the longitudinal data in the Health and Retirement Study (HRS).

- The policy outcomes module aggregates projections of individual-level outcomes into policy outcomes such as taxes, medical care costs, and pension benefits paid. This component takes account of public and private program rules to the extent allowed by the available outcomes. Because we have access to HRS-linked restricted data from Social Security records and employer pension plans, we are able to realistically model retirement benefit receipt.

Figure 1 provides a schematic overview of the model. This population simulation example starts in 2004 with an initial population aged 51+ taken from the HRS. We then predict outcomes using our estimated transition probabilities (See section 4). Those who survive make it to the end of that

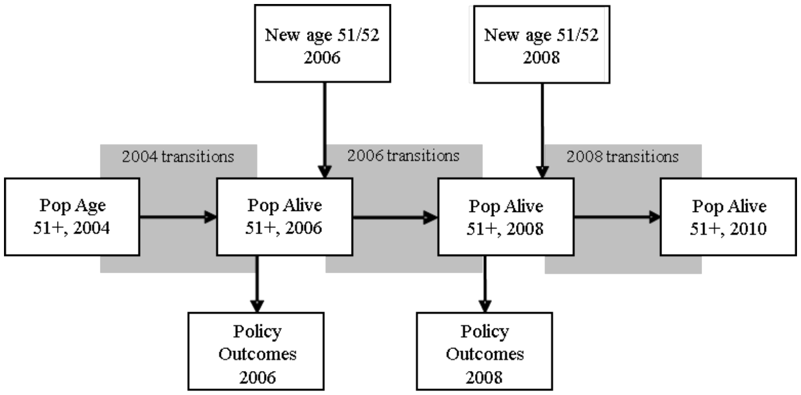

Figure 1: Architecture of the FEM

year, at which point we calculate policy outcomes for the year. We then move to the following time period (two years later), when a new cohort of 51 and 52 year-olds enters in case of a population simulation. This entrance forms the new age 51+ population, which then proceeds through the transition model as before. This process is repeated until we reach the final year of the simulation. In this paper we use a cohort simulation without new cohorts entering the simulation.

1.3 Comparison with other prominent microsimulation models of health expenditures

The FEM is unique among existing models that make health expenditure projections. It is the only model that projects health trends rather than health expenditures. It is also the only model that generates mortality out of assumptions on health trends rather than historical time series.

1.3.1 CBOLT Model

The Congressional Budget Office (CBO) uses time-series techniques to project health expenditure growth in the short term and then makes an assumption on long-term growth. They use a long term growth of excess costs of 2.3 percentage points starting in 2020 for Medicare. They then assume a reduction in excess cost growth in Medicare of 1.5% through 2083, leaving a rate of 0.9% in 2083. For non-Medicare spending they assume an annual decline of 4.5%, leading to an excess growth rate in 2083 of 0.1%.

1.3.2 Centers for Medicare and Medicaid Services

The Centers for Medicare and Medicaid Services (CMS) performs an extrapolation of medical expenditures over the first ten years, then computes a general equilibrium model for years 25 through 75 and linearly interpolates to identify medical expenditures in years 11 through 24 of their estimation. The core assumption they use is that excess growth of health expenditures will be one percentage point higher per year for years 25-75 (that is if nominal GDP growth is 4%, health care expenditure growth will be 5%).

2 Data sources used for estimation

The Health and Retirement Study is the main data source for the model. We supplemented this data with merged Social Security covered earnings histories and data on health care costs coming from 2 major health surveys in the U.S. We describe these surveys below and the samples we selected for the analysis. We first list the variables used in the analysis. We then give details on the data sources.

Estimated Outcomes in Initial Conditions Model

**Economic Outcomes**	**Health Outcomes**
Employment	Hypertension
Earnings	Heart Disease
Wealth	Self-Reported Health
Defined Contribution Pension Wealth	BMI Status
Pension Plan Type	Smoking Status
AIME	Functional Status
Social Security Quarters of Coverage	
Health Insurance	

Estimated Outcomes in/from Transition Model

**Economic Outcomes**	**Health Outcomes**	**Other Outcomes**
Employment	Death	Income Tax Revenue
Earnings	Heart	Social Security Revenue
Wealth	Stroke	Medicare Revenue
Demographics	Cancer	Medical Expenses
Health Insurance	Hypertension	Medicare Part A Expenses
Defined Benefit Claim	Diabetes	Medicare Part B Expenses
SSI Claim	Lung Disease	Medicare Part B Enrollment
Social Security Claim	Nursing Home	Medicare Part D Enrollment
	BMI	OASI Enrollment
	ADL Limitations	SSI enrollment
	IADL Limitations	Medicaid Enrollment
		Medicaid Expenditures

2.1 Health and Retirement Study

The Health and Retirement Study (HRS) waves 1998-2018 are used to estimate the transition model. Interviews occur every two years. We use the dataset created by RAND (RAND HRS, version 1992-2018 v2) as our basis for the analysis. We use all cohorts in the analysis and consider sampling weights whenever appropriate. When appropriately weighted, the HRS in 2016 is representative of U.S. households where at least one member is at least 51. The HRS is also used as the host data for the simulation (pop 51+ in 2016).

The HRS adds new cohorts every six years. Until recently, the latest available cohort had been added in 2016, which is why that is the FEM's base year.

2.2 Social Security covered earnings files

To get information on Social Security entitlements of respondents, we match the HRS data to the Social Security Covered Earnings files of 1992, 1993, 1998, 2004 and 2006 which provides information on earning histories of respondents as well as their entitlement to future Social Security benefits. We then construct the average indexed monthly earnings (AIME), the basis for the determination of benefit levels, from these earning histories. The AIME is constructed by first indexing using the National Wage Index (NWI) to the wage level when the respondent turns age 60. If this occurs after 2008, we project the evolution of the NWI using the average annual rate of change of the last 20 years (2.9% nominal). We then take the 35 highest years (if less than 35 years are available, remaining years are considered zero earning years) and take the average. We then convert back this annual amount on a monthly basis and convert back to $2004 U.S. dollars using the CPI. Quarters of coverage, which determine eligibility to Social Security, are defined as the sum of posted quarters to the file. A worker is eligible for Social Security if he has accumulated at least 40 quarters of coverage. A worker roughly accumulates a quarter of coverage for every $4000 of coverage earnings up to a maximum of 4 per year. Not all respondents agree to have their record matched. Hence, there is the potential for non-representativeness. However, recent studies show that the extent of non-representativeness is quite small and that appropriate weighting using HRS weights mostly corrects for this problem (Kapteyn et al., 2006).

2.3 Medical Expenditure Panel Survey

The Medical Expenditure Panel Survey (MEPS), beginning in 1996, is a set of large-scale surveys of families and individuals, their medical providers (doctors, hospitals, pharmacies, etc.), and employers across the United States. The Household Component (HC) of the MEPS provides data from individual households and their members, which is supplemented by data from their medical providers. The Household Component collects data from a representative sub sample of households drawn from the previous year's National Health Interview Survey (NHIS). Since NHIS does not include the institutionalized population, neither does MEPS: this implies that we can only use the MEPS to estimate medical costs for the non-elderly population. Information collected during household interviews include: demographic characteristics, health conditions, health status, use of medical services, sources of medical payments, and body weight and height. Each year the household survey includes approximately 12,000 households or 34,000 individuals. Sample size for those aged 51-64 is about 4,500. MEPS has comparable measures of social-economic status (SES) variables as those in HRS, including age, race/ethnicity, educational level, census region, and marital status.

FEM uses MEPS years 2007-2010 for cost estimation. See Section 5.1 for a description. FEM also

2.4 Medicare Current Beneficiary Survey

The Medicare Current Beneficiary Survey (MCBS) is a nationally representative sample of aged, disabled and institutionalized Medicare beneficiaries. The MCBS attempts to interview each respondent twelve times over three years, regardless of whether he or she resides in the community, a facility, or transitions between community and facility settings. The disabled (under 65 years of age) and oldest-old (85 years of age or older) are over-sampled. The first round of interviewing was conducted in 1991. Originally, the survey was a longitudinal sample with periodic supplements and indefinite periods of participation. In 1994, the MCBS switched to a rotating panel design

with limited periods of participation. Each fall a new panel is introduced, with a target sample size of 12,000 respondents and each summer a panel is retired. Institutionalized respondents are interviewed by proxy. The MCBS contains comprehensive self-reported information on the health status, health care use and expenditures, health insurance coverage, and socioeconomic and demographic characteristics of the entire spectrum of Medicare beneficiaries. Medicare claims data for beneficiaries enrolled in fee-for-service plans are also used to provide more accurate information on health care use and expenditures. MCBS years 2007-2012 are used for estimating medical cost models. See section 5.1 for discussion.

3 Data sources for trends and baseline scenario

Two types of trends need to be projected in the model. First, we need to project trends in the incoming cohorts (the future new age 51/52 individuals). This includes trends in health and economic outcomes. Second, we need to project excess aggregate growth in real income and excess growth in health spending.

3.1 Data for growth in wages

Earnings outcomes in the simulation are in real 2010 dollars, that are adjusted for real wage growth using historical real wage differential data between 2010 and 2016 (the start of the simulation). For 2017 and later, intermediate projections are used (SSA 2017 Trustees Report, table V.B1). Outcomes are subsequently adjusted for inflation to 2024 dollars.

3.2 Demographic adjustments

We make adjustments to the weighting in the HRS to match population counts, since we deleted some cases from the data and only considered the set of respondents with matched Social Security records; this takes account of selectivity based on these characteristics. We post-stratify the HRS sample by 5 year age groups, gender and race and rebalance weights using the 2016 American Community Survey estimates.

4 Estimation

In this section we describe the approach used to estimate the transition model, the core of the FEM.

4.1 Transition model

We consider a large set of outcomes for which we model transitions. Table 1 gives the set of outcomes considered for the transition model along with descriptive statistics and the population at risk when estimating the relationships.

Since we have a stock sample from the age 51+ population, each respondent goes through an individual-specific series of intervals. Hence, we have an unbalanced panel over the age range starting from 51 years old. Denote by j_{i0} the first age at which respondent i is observed and j_{iT_i} the last age when he is observed. Hence we observe outcomes at ages $j_i = j_{i0}, \ldots, j_{iT_i}$.

We first start with discrete outcomes which are absorbing states (e.g. disease diagnostic, mortality, benefit claiming). Record as $h_{i,j_i,m} = 1$ if the individual outcome m has occurred as of age j_i.

We assume the individual-specific component of the hazard can be decomposed in a time invariant and variant part. The time invariant part is composed of the effect of observed characteristics x_i that are constant over the entire life course and initial conditions $h_{i,j_0,-m}$ (outcomes other than the outcome m) that are determined before the first age in which each individual is observed The time-varying part is the effect of previously diagnosed outcomes $h_{i,j_i-1,-m}$, on the hazard for m.[1] We assume an index of the form $z_{m,j_i} = x_i\beta_m + h_{i,j_i-1,-m}\gamma_m + h_{i,j_0,-m}\psi_m$. Hence, the latent component of the hazard is modeled as

$$h^*_{i,j_i,m} = x_i\beta_m + h_{i,j_i-1,-m}\gamma_m + h_{i,j_0,-m}\psi_m + a_{m,j_i} + \varepsilon_{i,j_i,m}, \tag{1}$$

$$m = 1,\ldots,M_0,\ j_i = j_{i0},\ldots,j_{i,T_i},\ i = 1,\ldots,N$$

The term $\varepsilon_{i,j_i,m}$ is a time-varying shock specific to age j_i. We assume that this last shock is normally distributed and uncorrelated across diseases. We approximate a_{m,j_i} with an age spline. After several specification checks, knots at age 65 and 75 appear to provide the best fit. This simplification is made for computational reasons since the joint estimation with unrestricted age fixed effects for each condition would imply a large number of parameters. The absorbing outcome, conditional on being at risk, is defined as

$$h_{i,j_i,m} = \max\{I(h^*_{i,j_i,m} > 0), h_{i,j_i-1,m}\}$$

The occurrence of mortality censors observation of other outcomes in a current year. Mortality is recorded from exit interviews.

A number of restrictions are placed on the way feedback is allowed in the model. Table 2 documents restrictions placed on the transition model. We also include a set of other controls. A list of such controls is given in Table 3 along with descriptive statistics.

We have three other types of outcomes:

1. First, we have binary outcomes which are not an absorbing state, such as living in a nursing home. We specify latent indices as in (1) for these outcomes as well but where the lag dependent outcome also appears as a right-hand side variable. This allows for state-dependence.

2. Second, we have ordered outcomes. These outcomes are also modeled as in (1) recognizing the observation rule is a function of unknown thresholds ς_m. Similarly to binary outcomes, we allow for state-dependence by including the lagged outcome on the right-hand side.

3. The third type of outcomes we consider are censored outcomes, earnings and financial wealth. Earnings are only observed when individuals work. For wealth, there are a non-negligible number of observations with zero and negative wealth. For these, we consider two part models where the latent variable is specified as in (1) but model probabilities only when censoring does not occur. In total, we have M outcomes.

The parameters $\theta_1 = \left(\{\beta_m, \gamma_m, \psi_m, \varsigma_m\}_{m=1}^{M},\right)$, can be estimated by maximum likelihood. Given the normality distribution assumption on the time-varying unobservable, the joint probability of all time-intervals until failure, right-censoring or death conditional on the initial conditions $h_{i,j_0,-m}$ is the product of normal univariate probabilities. Since these sequences, conditional on initial conditions, are also independent across diseases, the joint probability over all disease-specific sequences is simply the product of those probabilities.

[1] With some abuse of notation, $j_i - 1$ denotes the previous age at which the respondent was observed.

For a given respondent observed from initial age j_{i0} to a last age j_{T_i}, the probability of the observed health history is (omitting the conditioning on covariates for notational simplicity)

$$l_i^{-0}(\theta; h_{i,j_{i0}}) = \left[\prod_{m=1}^{M-1}\prod_{j=j_{i1}}^{j_{T_i}} P_{ij,m}(\theta)^{(1-h_{ij-1,m})(1-h_{ij,M})}\right] \times \left[\prod_{j=j_{i1}}^{j_{T_i}} P_{ij,M}(\theta)\right]$$

We use the -0 superscript to make explicit the conditioning on $\mathbf{h}_{i,j_{i0}} = (h_{i,j_{i0},0}, \ldots, h_{i,j_{i0},M})'$. We have limited information on outcomes prior to this age. The likelihood is a product of M terms with the mth term containing only $(\beta_m, \gamma_m, \psi_m, \varsigma_m)$. This allows the estimation to be done seperately for each outcome.

4.1.1 Inverse Hyperbolic Sine Transformation

One problem fitting the wealth and earnings distribution is that they have a long right tail and wealth has some negative values. We use a generalization of the inverse hyperbolic sine transform (IHT) presented in MacKinnon and Magee (1990). First denote the variable of interest y. The hyperbolic sine transform is

$$y = \sinh(x) = \frac{\exp(x) - \exp(-x)}{2} \tag{2}$$

The inverse of the hyperbolic sine transform is

$$x = \sinh^{-1}(y) = h(y) = \log(y + (1 + y^2)^{1/2})$$

Consider the inverse transformation. We can generalize such transformation, first allowing for a shape parameter θ,

$$r(y) = h(\theta y)/\theta \tag{3}$$

Such that we can specify the regression model as

$$r(y) = x\beta + \varepsilon, \varepsilon \sim \mathrm{N}(0, \sigma^2) \tag{4}$$

A further generalization is to introduce a location parameter ω such that the new transformation becomes

$$g(y) = \frac{h(\theta(y + \omega)) - h(\theta\omega)}{\theta h'(\theta\omega)} \tag{5}$$

where $h'(a) = (1 + a^2)^{-1/2}$.

We specify (4) in terms of the transformation g. The shape parameters can be estimated from the concentrated likelihood for θ, ω. We can then retrieve β, σ by standard OLS.

Upon estimation, we can simulate

$$\tilde{g} = x\hat{\beta} + \sigma\tilde{\eta}$$

where η is a standard normal draw. Given this draw, we can retransform using (5) and (2)

$$h(\theta(y + \omega)) = \theta h'(\theta\omega)\tilde{g} + h(\theta\omega)$$
$$\tilde{y} = \frac{\sinh\left[\theta h'(\theta\omega)\tilde{g} + h(\theta\omega)\right] - \theta\omega}{\theta}$$

4.2 Health Utility Index Mark 3

As an alternative measure of life expectancy, we compute the Health Utility Index Mark 3 (HUI3) score, a health-related quality-of-life (HRQoL) measure. Horsman et al. (2003) provide an overview of HUI systems with supporting references. The 33-item HUI3 questionnaire (HUI3SU.33Q) was included as experimental module 7 in the 2000 Health and Retirement Study (HRS) wave. The Schaeffer Center for Health Policy & Economics obtained the HUI3 scoring procedures (Furlong et al., 2001) from HUInc. and applied them to the questionnaire responses in the 2000 HRS. See section 7.1 for more details. HUI3 is an alternative measure of Quality-Adjusted Life Years, but scores include a cognitive component, which better represents the effects of cognitive changes on quality of life.

5 Medical costs

5.1 Medical costs estimation

In the FEM, a cost module links a person's current state–demographics, economic status, current health, risk factors, and functional status to 4 types of individual medical spending. The FEM models: total medical spending (medical spending from all payment sources), Medicare spending[2], Medicaid spending (medical spending paid by Medicaid), and out of pocket spending (medical spending by the respondent). These estimates are based on pooled weighted least squares regressions of each type of spending on risk factors, self-reported conditions, and functional status, with spending inflated to constant dollars using the medical component of the consumer price index. We use the 2007-2010 Medical Expenditure Panel Survey for these regressions for persons not Medicare eligible, and the 2007-2012 Medicare Current Beneficiary Survey for spending for those that are eligible for Medicare. Those eligible for Medicare include people eligible due to age (65+) or due to disability status. Comparisons of prevalences and question wording across these different sources are provided in Tables 4 and 5, respectively.

In the baseline scenario, this spending estimate can be interpreted as the resources consumed by the individual given the manner in which medicine is practiced in the United States during the post-part D era (2006-2010). Models are estimated for total, Medicaid, out of pocket spending, and for the Medicare spending. These estimates only use the MCBS dataset.

Since both the MEPS and MCBS are known to under-predict medical spending (see, e.g., Selden and Sing, 2008, and references therein), we applied adjustment factors to the predicted three types of individual medical spending so that the predicted per-capita spending in FEM equal the corresponding spending in National Health Expenditure Accounts (NHEA) for age group 55-64 in year 2004 and ages 65 and over in year 2010, respectively. Table 6 shows how these adjustment factors were determined by using the ratio of expenditures in the NHEA to expenditures predicted in the FEM.

6 Implementation

The FEM is implemented in multiple parts. Estimation of the transition and cross sectional models is performed in Stata. The incoming cohort model is estimated in Stata using the CMP package

[2]We estimate annual medical spending paid by specific parts of Medicare (Parts A, B, and D) and sum to get the total Medicare expenditures.

(Roodman, 2011). The simulation is implemented in C++ to increase speed.

To match the two year structure of the Health and Retirement Study (HRS) data used to estimate the transition models, the FEM simulation proceeds in two year increments. The end of each two year step is designed to occur on July 1st to allow for easier matching to population forecasts from Social Security. A simulation of the FEM proceeds by first loading a population representative of the age 51+ US population in 2016, generated from HRS. In two year increments, the FEM applies the transition models for mortality, health, working, wealth, earnings, and benefit claiming with Monte Carlo decisions to calculate the new states of the population. If incoming cohorts are being used, the new 51/52 year olds are added to the population. The number of new 51/52 year olds added is consistent with estimates from the Census, stratified by race. Once the new states have been determined and new 51/52 year olds added, the cross sectional models for medical costs, and calculations for government expenditures and revenues are performed. Summary variables are then computed. Computation of medical costs includes the persons that died to account for end of life costs. Other computations, such as Social Security benefits and government tax revenues, are restricted to persons alive at the end of each two year interval. To eliminate uncertainty due to the Monte Carlo decision rules, the simulation is performed multiple times (here 75), and the mean of each summary variable is calculated across repetitions.

FEM simulation takes as inputs assumptions regarding growth in the national wage index, normal retirement age, interest rates, cost of living adjustments, the consumer price index, significant gainful activity, and deferred retirement credit. The default assumptions are taken from the 2010 Social Security Intermediate scenario, adjusted for no price increases after 2010. Therefore simulation results are in real 2010 dollars. Table 7 shows the assumptions for each calendar year and Table 8 shows assumptions for each birth year.

Different simulation scenarios are implemented by changing any of the following components: incoming cohort model, transition models, interventions that adjust the probabilities of specific transition, and changes to assumptions on future economic conditions.

6.1 Intervention Module

The intervention module can adjust characteristics of individuals when they are first read into the simulation "init_interventions" or alter transitions within the simulation "interventions." At present, init_interventions can act on chronic diseases, ADL/IADL status, program participation, and some demographic characteristics. Interventions within the simulation can currently act on mortality, chronic diseases, and some program participation variables.

Interventions can take several forms. The most commonly used is an adjustment to a transition probability. One can also delay the assignment of a chronic condition or cure an existing chronic condition. Additional flexibility comes from selecting who is eligible for the intervention. Some examples might help to make the interventions concrete.

- Example 1: Delay the enrollment into Social Security OASI by two years. In this scenario claiming of Social Security benefits is transitioned as normal. However, if a person is predicted to claim their benefits, then that status is not immediately assigned, but is instead assigned two years later.

- Example 2: Cure hypertension for those with no other chronic diseases. In this scenario any individual with hypertension (including those who have had hypertension for many years) is cured (hypertension status is set to 0), as long as they do not have other chronic diseases.

This example uses the individuals chronic disease status as the eligibility criteria for the intervention.

- Example 3: Reduce the incidence of hypertension for half of men aged 55 to 65 by 10% in the first year of the simulation, gradually increasing the reduction to 20% after 10 years. This example begins to show the flexibility in the intervention module. The eligibility criteria are more complex (half of men in a specific age range are eligible) and the intervention changes over time. Mathematically, the intervention works by acting on the incidence probability, ρ. In the first year of the simulation, the probability is replaced by $(1 - 0.5 * 0.1)\,\rho = 0.95\rho$. The binary outcome is then assigned based on this new probability. Thus, at the population level, there is a 5% reduction in incidence for men aged 55 to 65, as desired. After 10 years, the probability for this eligible population becomes $(1 - 0.5 * 0.2)\,\rho = 0.9\rho$.

More elaborate interventions can be programmed by the user.

7 Model development

7.1 Health Utility Index Mark 3

7.1.1 Data description

HUI3 is a measure of healthy life years encompassing eight dimensions of health. The variables *hui3vl*, *hui3hl*, *hui3sl*, *hui3al*, *hui3dl*, *hui3el*, *hui3cl*, and *hui3pl*, were derived from the 2000 HRS module 7 responses according to instructions in the HUI3 manual (Furlong et al., 2001). The variables correspond to vision, hearing, speech, ambulation, dexterity, emotion, cognition, and pain, respectively, with each dimension having five or six attribute levels. Information about these attribute levels, and the mapping from attribute levels to a single-attribute and multi-attribute HUI3 score can be found in Feeny et al. (1995, 1996, 2002) and Furlong et al. (1998). Each single-attribute utility score is defined to be in the range from 0 (most morbidity) to 1 (no morbidity). The multi-attribute utility score is defined to be in the range from -0.36 to 1. A score of 0 is equivalent to death, 1 is perfect health, and negative scores represent health states considered worse than death.

For some of the respondents in the 2000 HRS module, an exact HUI3 attribute level could not be derived because one or more responses in the block of questions was missing or incomplete. Without an attribute level, it is impossible to compute the single- and multi-attribute utility scores. The number of missing cases are 10, 66, 1, 2, 1, 14, 1 and 3, for the variables vision, hearing, speech, ambulation, dexterity, emotion, cognition and pain, respectively. The overall utility score is missing for 90 cases, leaving 1,066 nonmissing cases. While the HUI3 manual discusses options for handling missing data, it does not provide any specific rules for imputation (Furlong et al., 2001). Below we describe the two steps in our imputation strategy.

Additionally, since the HUI3 questionnaire is only administered during the 2000 HRS wave to 1,156 respondents, we used the parameter estimates in Table 9 to predict HUI3 scores for the entire FEM simulation sample.

7.1.2 Missing data: Logical limits

By considering a respondent's nonmissing responses for an attribute, we find that there is a logical lower and upper limit to what their attribute level could be had they responded completely to the entire block of questions. Using the lower and upper limits of the attribute level, the logical

limits for the single- and multi-attribute utility score were also derived, following the mapping and formula in Feeny et al. (2002). The lower and upper attribute level limits are the same in some cases, resulting in a logical imputation of the exact attribute level value. These logical imputations reduced the number of missing overall utility scores and overall health states by 6 cases to a total of 1,072 nonmissing cases.

7.1.3 Missing data: Conditionally-almost-sure imputations

To develop the second step in the imputation model for missing attribute levels, we used HRS data to estimate a probability distribution for the attribute level. This probability distribution is conditional upon a respondent's observed questionnaire responses in the block of questions for the attribute. For some attributes, HRS respondents who have the same pattern of observed responses and have a nonmissing attribute level all share the same attribute level. If we assume the attribute level is missing-at-random, then the empirical estimate is a degenerate distribution, assigning 100% probability to a single attribute level.

These cases were identified and their attribute levels were imputed at the level with 100% conditional probability. Using imputed attribute levels, the single- and multi-attribute utility score were also derived. The second step reduced the number of missing overall utility scores and overall health states by 60 cases to a total of 1,132 nonmissing cases.

8 Validation

We perform two validation exercises:

1. Cross-validation
2. External corroboration

Cross-validation is a test of the simulations internal validity that compares simulated outcomes to actual outcomes, and external corroboration compares model forecasts to others' forecasts.

8.1 Cross-validation

The cross-validation exercise randomly samples half of the HRS respondent IDs for use in estimating the transition models. The respondents not used for estimation, but who were present in the HRS sample in 1998, are then simulated from 1998 through 2018. Demographic, health, and economic outcomes are compared between the simulated (”FEM”) and actual (”HRS”) cohorts. These results are presented in Table 10 - Table 13 for 2000, 2006, 2012, and 2018 with a statistical test of the difference between the average values in the two cohorts.

Worth noting is how the composition of the cohort changes in this exercise. In 1998, the sample represents those 51 and older. Since we follow a fixed cohort, the average age of the cohort will increase to 71 and older in 2018 (Table 10). This has consequences for some measures in later years where the eligible cohort shrinks.

8.1.1 Mortality and Nursing Home Status

Mortality rates are similar between the FEM simulation and HRS survey cohorts (Table 10). The prevalence rate of living in a nursing home is slightly underestimated in 2012 (by 0.6 percentage points) and overestimated in 2018 (by 0.8 percentage points) in the FEM compared to the HRS.

8.1.2 Demographics

Demographic measures are presented in Table 11. The gender balance and fraction of the cohort that is male, non-Hispanic Black, or Hispanic is consistent between the FEM and HRS.

8.1.3 Health Outcomes

The two cohorts are not statistically different from each other for prevalence of most health outcomes in 2018. Lung disease prevalence was approximately 2.3 percentage points higher in the FEM cohort than in the HRS cohort (see Table 12).

8.1.4 Health Risk Factors

Average BMI and the prevalence of ever-smokers is similar between the FEM and HRS cohorts in 2018 (see Table 13).

8.1.5 Economic Outcomes

Claiming Old Age and Survivor's Insurance benefits is slightly lower at the start of the simulation, but ends apprixmately 1 percentage point higher than the HRS cohort in 2018. Claiming disability is underestimated in the year that most simulants have reached retirement age (2006). The fraction of the cohort working for pay is similar between the two cohorts (see Table 14). Estimates of earnings for those who are still working are lower for earlier years in FEM compared to the HRS cohort, but similar for years 2012 and 2018 (See Table 15).

On the whole, the cross-validation exercise is reassuring. Comparing simulated outcomes to actual outcomes using a set of transition models estimated on a separate population reveals that the majority of outcomes of interest are not statistically different. In cases where they are, the practical difference is potentially low.

8.2 External Corroboration

Finally, we compare FEM population forecasts to Census forecasts of the US population.. Here, we focus on the full HRS population (51 and older) and those 65 and older. For this exercise, we begin the simulation in 2010 and simulate the full population through 2050. Population projections are compared to the 2012 Census projections for years 2012 through 2050. FEM population forecasts are always within two percent of Census forecasts.

9 Baseline Forecasts

In this section we present baseline forecasts of the Future Elderly Model. The figures show data from the HRS for the 55+ population from 1998 through 2012 and forecasts from the FEM for the 55+ population beginning in 2010.

9.1 Disease Prevalence

Figure 2 depicts the six chronic conditions we project for men. And Figure 3 depicts the historic and forecasted values for women.

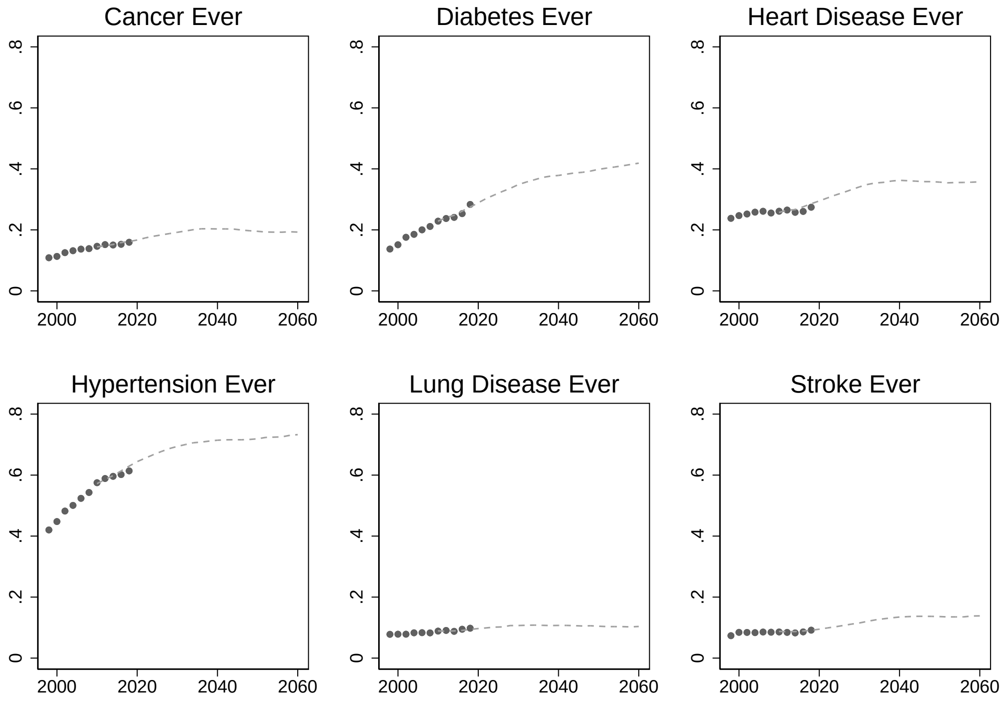

Figure 2: Historic and Forecasted Chronic Disease Prevalence for Men 55+

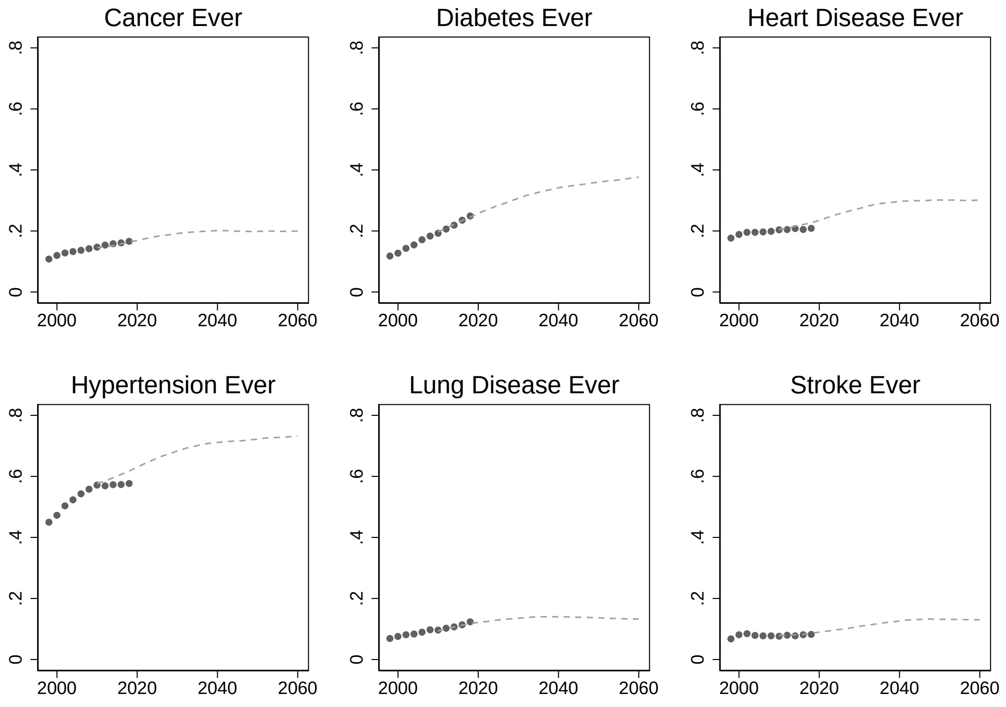

Figure 3: Historic and Forecasted Chronic Disease Prevalence for Women 55+

Figure 4 shows historic and forecasted levels for any ADL difficulties, three or more ADL difficulties, any IADL difficulties, and two or more IADL difficulties for men 55 and older. Figure 5

shows historic and forecasted levels for any ADL difficulties, three or more ADL difficulties, any IADL difficulties, and two or more IADL difficulties for women 55 and older.

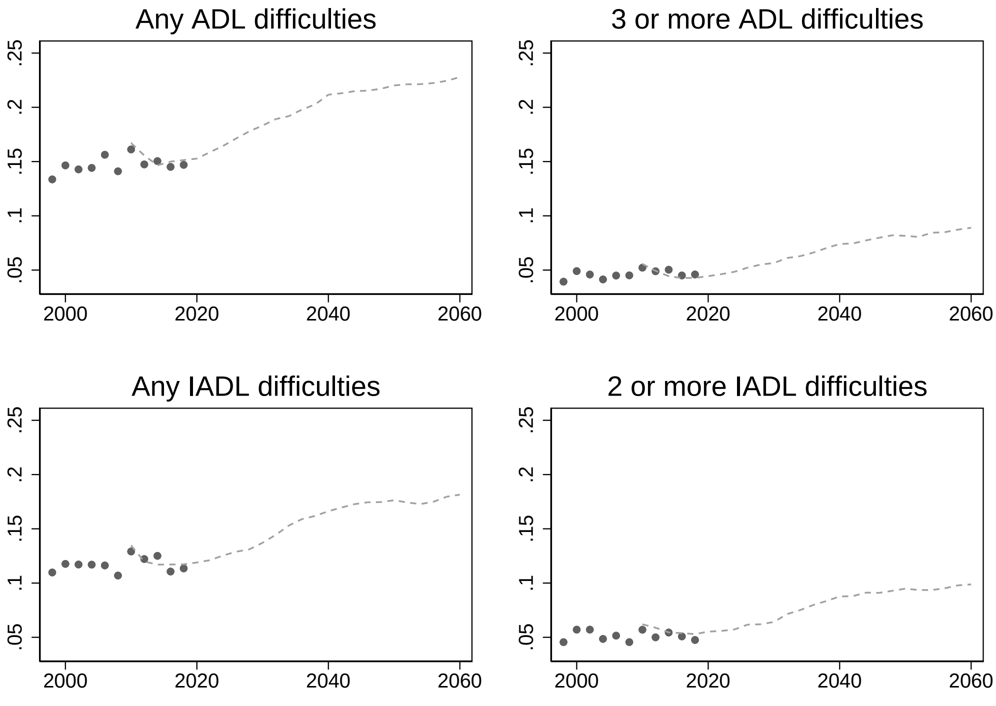

Figure 4: Historic and Forecasted ADL and IADL Prevalence for Men 55+

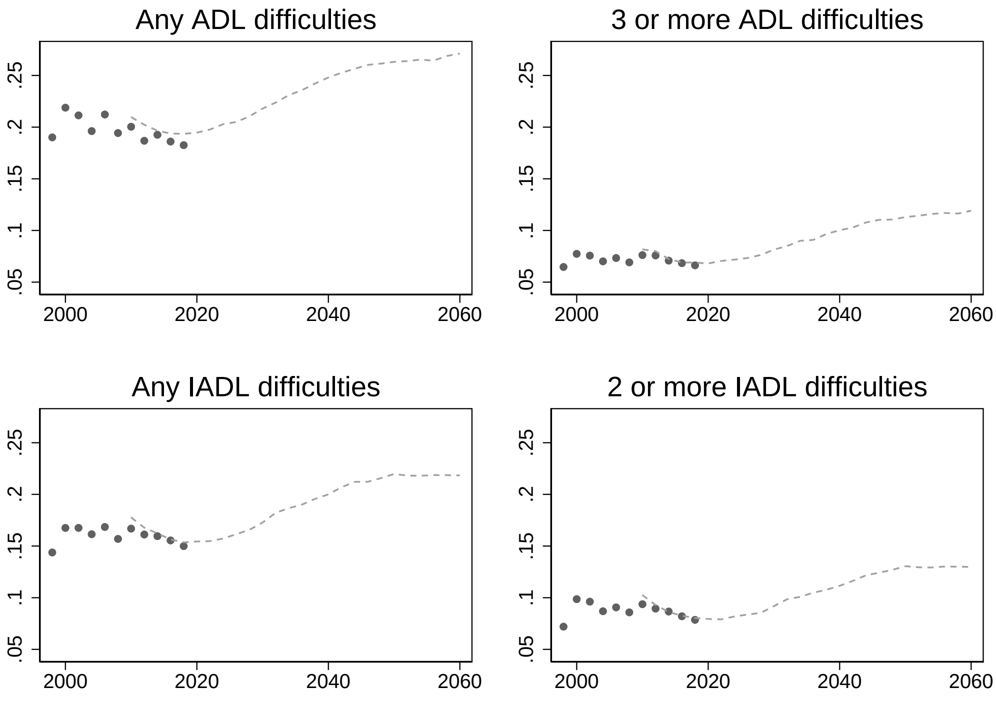

Figure 5: Historic and Forecasted ADL and IADL Prevalence for Women 55+

10 Acknowledgments

The Future Elderly Model has been developed by a large team over the last decade. Jay Bhattacharya, Eileen Crimmins, Christine Eibner, Étienne Gaudette, Geoff Joyce, Darius Lakdawalla, Pierre-Carl Michaud, and Julie Zissimopoulos have all provided expert guidance. Adam Gailey, Baoping Shang, and Igor Vaynman provided programming and analytic support during the first years of development at RAND. In more recent years, the University of Southern California research programming team has supported model development. These programmers include Patricia St. Clair, Laura Gascue, Henu Zhao, and Yuhui Zheng. Barbara Blaylock and Wendy Cheng have greatly aided model development while working as research assistants at USC.

11 Tables

			Type	At risk	Mean/fraction
Disease	heart disease		biennial incidence	undiagnosed	0.03
	hypertension		biennial incidence	undiagnosed	0.04
	stroke		biennial incidence	undiagnosed	0.01
	lung disease		biennial incidence	undiagnosed	0.01
	cancer		biennial incidence	undiagnosed	0.02
	diabetes		biennial incidence	undiagnosed	0.02
Risk Factors	Smoking Status	never smoked	ordered	all	0.43
		ex smoker	ordered	all	0.43
		current smoker	ordered	all	0.14
	Log BMI		continuous	all	3.31
	ADL Status	no ADLs	ordered	all	0.77
		1 ADL	ordered	all	0.08
		2 ADLS	ordered	all	0.04
		3+ ADLS	ordered	all	0.06
	IADL Status	no IADLs	ordered	all	0.79
		1 IADL	ordered	all	0.08
		2+ IADLs	ordered	all	0.08
LFP & Benefits	working		prevalence	age < 80	0.48
	DB pension receipt		biennial incidence	eligible & not receiving	0.12
	SS benefit receipt		biennial incidence	eligible & not receiving	0.08
	DI benefit receipt		prevalence	eligible & age < 65	0.04
	Any health insurance		prevalence	age < 65	0.87
	SSI receipt		prevalence	all	0.03
	Nursing Home residency		prevalence	all	0.02
	Death		biennial incidence	all	0.07
Financial Resources ($USD 2004)	financial wealth		median	all non-zero wealth	186,860.41
	earnings		median	all working	5,148.73
	wealth positive		prevalence	all	0.96

Table 1: Outcomes in the transition model. Estimation sample is HRS 1991-2008 waves.

Value at time $T-1$	Outcome at time T																			
	Heart disease	hypertension	stroke	Lung disease	diabetes	cancer	disability	mortality	Smoking status	BMI	Any HI	DI Claim	SS Claim	DB Claim	SSI Claim	Nursing Home	Work	Earnings	Nonzero Wealth	Wealth
Heart disease			✓				✓	✓	✓	✓	✓	✓	✓	✓	✓	✓	✓	✓	✓	✓
Blood pressure	✓		✓				✓	✓	✓	✓	✓	✓	✓	✓	✓	✓	✓	✓	✓	✓
Stroke							✓	✓	✓	✓	✓	✓	✓	✓	✓	✓	✓	✓	✓	✓
Lung disease							✓	✓	✓	✓	✓	✓	✓	✓	✓	✓	✓	✓	✓	✓
Diabetes	✓	✓	✓				✓	✓	✓	✓	✓	✓	✓	✓	✓	✓	✓	✓	✓	✓
Cancer			✓				✓	✓	✓	✓	✓	✓	✓	✓	✓	✓	✓	✓	✓	✓
Disability							✓	✓	✓	✓	✓	✓	✓	✓	✓	✓	✓	✓	✓	✓
Claimed DI											✓	✓	✓	✓	✓		✓	✓	✓	✓
Claimed SS											✓			✓	✓		✓	✓	✓	✓
Claimed DB													✓		✓		✓	✓	✓	✓
Claimed SSI															✓					
Work											✓	✓	✓		✓		✓	✓	✓	✓
Earnings											✓	✓	✓	✓	✓		✓	✓	✓	✓
Nonzero wealth											✓	✓	✓	✓	✓	✓	✓	✓	✓	✓
Wealth											✓	✓	✓	✓	✓	✓	✓	✓	✓	✓
Nursing home stay															✓	✓			✓	✓

Table 2: Restrictions on transition model. ✓indicates that an outcome at time $T-1$ is allowed in the transition model for an outcome at time T.

	Unweighted Statistics			
Control variable	Mean	Standard deviation	Minimum	Maximum
Non-Hispanic Black	0.138	0.345	0	1
Hispanic	0.0887	0.284	0	1
Less than high school	0.250	0.433	0	1
Some college and above	0.397	0.489	0	1
Male	0.436	0.496	0	1
Ever smoked	0.590	0.492	0	1
Fitted values	1864	1113	-97.01	4350
frq				
Init.of Any DB from current job	0.173	0.378	0	1
fnra3	0.0723	0.259	0	1
fnra4	0.0450	0.207	0	1
fnra5	0.0965	0.295	0	1
Any DC from current job	0.109	0.311	0	1
(IHT of DC wlth in 1000s)/100 if any DC, zero otherwise	0.000132	0.00237	0	0.0688

Table 3: Desciptive statistics for exogeneous control variables in 2004 HRS ages 51+ sample used as simulation stock population

	Prevalence %							
Source (years, ages)	Cancer	Heart Diseases	Stroke	Diabetes	Hypertension	Lung Disease	Overweight	Obese
HRS (1991-2008, 55-64)	9%	14%	4%	17%	45%	7%	38%	34%
NHIS (1997-2010, 55-64)	8%	17%	4%	14%	44%	8%	37%	33%
MEPS (2000-2010, 55-64)	7%	17%	4%	15%	47%	7%	38%	32%
HRS (1991-2008, 65+)	18%	30%	10%	21%	60%	11%	38%	25%
NHIS (1997-2010, 65+)	16%	31%	9%	17%	56%	10%	36%	25%
MCBS (2000-2010, 65+)	18%	40%	11%	23%	65%	16%	38%	23%
MEPS (2000-2010, 65+)	12%	33%	11%	19%	64%	10%	38%	25%

Table 4: Health condition prevalences in survey data

Disease	Survey: HRS	NHIS	MEPS	MCBS
Cancer	Has a doctor ever told you that you have cancer or a malignant tumor, excluding minor skin cancers?	Have you ever been told be a doctor or other health professional that you had cancer or a malignanacy of any kind? (WHEN RECODED, SKIN CANCERS WERE EXCLUDED)	List all the conditions that have bothered (the person) from (START time) to (END time) CCS codes for the conditions list are 11-21, 24-45	Has a doctor ever told you that you had any (other) kind of cancer malignancy, or tumor other than skin cancer?
Heart Diseases	Has a doctor ever told you that you had a heart attack, coronary heart disease, angina, congestive heart failure, or other heart problems?	Four separate questions were asked about whether ever told by a docotor or oither health professional that had: CHD, Angina, MI, other heart problems.	Have you ever been told by a doctor or health professional that you have CHD; Angina; MI; other heart problems	Six separate questions were asked about whether ever told by a doctor that had: Angina or MI; CHD; other heart problems (included four questions)
Stroke	Has a doctor ever told you that you had a stroke?	Have you EVER been told by a doctor or other health professional that you had a stroke?	If Female, add: [Other than during pregnancy,] Have you ever been told by a doctor or health professional that you have a stroke or TIA (transient ischemic attack)	[Since (PREV< SUPP. RD. INT. DATE),] has a doctor (ever) told (you/SP) that (you/he/she) had a stroke, a brain hemorrhage, or a cerebrovascular accident?
Diabetes	Has a doctor ever told you that you have diabetes or high blood sugar?	If Female, add: [Other than during pregnancy,] Have you ever been told by a doctor or health professional that you have diabetes or sugar diabetes?	If Female, add: [Other than during pregnancy,] Have you hever been told by a doctor or health professional that you have diabetes or sugar diabetes?	Has a doctor (ever) told (you/SP) that (you/he/she) had diabetes, high blood sugar, or sugar in (your/his/her) urine? [DO NOT INCLUDE BOERDERLINE PREGNANCY, OR PREDIABETEIC DIABETES.]
Hypertension	Has a doctor ver told you that you have high blood pressure or hypertension?	Have you EVER been told by a doctor or other health professional that you had Hypertension, also called high blood pressure?	Have you EVER been told by a doctor or other health professional that you had Hypertension, also called high blood pressure?	Has a doctor (ever) told (you/SP) that (you/he/she) (still) (had) (have/has) hypertension, sometimes called high blood pressure?
Lung Disease	Has a doctor ever told you that you have chronic lung disease such a schronic bronchitis or emphysema? [IWER: DO NOT INCLUDE ASTHMA]	Question 1: During the PAST 12 MONTHS, have you ever been told by a doctor or other health professional that you had chronic bronchitis? Question 2: Have you EVER been told by a docotor or other health professional that you had emphysema?	List all the conditions that have bothered (the person) from (START time) to (END time) CCS codes for the conditions list are 127, 129-312	Has a doctor (ever) told (you/SP) that (you/he/she) had emphysema, asthma, or COPD? [COPD=CHRONIC OBSTRUCTIVE PULMONARY DISEASE.]
Overweight	Self-reported body weight and height			
Obese				

Table 5: Survey questions used to determine health conditions

	Ages 55-64			Ages 65 and over		
Payment sources	NHEA 2004 ($) (A)	FEM 2004, unadjusted ($) (B)	Adjustment factor (A)/(B)	NHEA 2010 ($) (C)	FEM 2010, unadjusted ($) (D)	Adjustment factor (C)/(D)
Total	7787.00	6678.00	1.17	18424.00	14690.00	1.25
Medicare	706.00	572.00	1.23	10016.00	7933.00	1.26
Medicaid	1026.00	579.00	1.77	2047.00	1168.00	1.75

Table 6: Per capita medical spending by payment source, age group, and year

Calendar year	National Wage Index	Real interest rate on wealth	COLA	Consumer Price Index	Substantial Gainful Activity	Y-o-Y excess real growth in medical costs
2004	35648.55	154.7553	3.606042	188.9	9720	.015
2005	36952.94	157.0766	3.703405	195.3	9960	.0148
2006	38651.41	158.3332	3.855245	201.6	10320	.0147
2007	40405.48	160.0749	3.982468	207.342	10800	.0145
2008	41334.97	163.1163	4.074064	215.303	11280	.0143
2009	40711.61	163.7688	4.31036	214.537	11760	.0141
2010	41673.83	171.4659	4.31036	214.537	12000	.0139
2011	41498.8	173.6949	4.31036	214.537	12000	.0138
2012	42005.09	176.4741	4.31036	214.537	12120	.0136
2013	41921.08	180.533	4.31036	214.537	12480	.0134
2014	42826.57	185.2268	4.31036	214.537	12840	.0133
2015	44543.92	190.7836	4.31036	214.537	13080	.0131
2016	45301.16	196.8887	4.31036	214.537	12699.34	.0129
2017	46134.7	202.5985	4.31036	214.537	12827.86	.0128
2018	47020.49	208.2712	4.31036	214.537	12961.05	.0126
2019	47904.48	214.1028	4.31036	214.537	13096.16	.0124
2020	48800.29	220.0977	4.31036	214.537	13233.25	.0122
2021	49585.97	226.2604	4.31036	214.537	13373.07	.0121
2022	50235.55	232.5957	4.31036	214.537	13512.89	.0119
2023	50803.21	239.1084	4.31036	214.537	13650.72	.0117
2024	51412.85	245.8035	4.31036	214.537	13794.57	.0115
2025	52045.23	252.6859	4.31036	214.537	13941.41	.0114
2026	52680.18	259.7611	4.31036	214.537	14089.9	.0112
2027	53312.34	267.0345	4.31036	214.537	14241.72	.011
2028	53952.09	274.5114	4.31036	214.537	14395.28	.0109
2029	54599.52	282.1978	4.31036	214.537	14550.68	.0107
2030	55254.71	290.0993	4.31036	214.537	14708.93	.0105
2031	55917.77	298.2221	4.31036	214.537	14870.88	.0104
2032	56588.78	306.5723	4.31036	214.537	15035.67	.0101
2033	57267.84	315.1563	4.31036	214.537	15204.43	.01
2034	57955.06	323.9807	4.31036	214.537	15374.82	.0097
2035	58650.52	333.0521	4.31036	214.537	15546.86	.0094
2036	59354.33	342.3776	4.31036	214.537	15720.37	.0091
2037	60066.58	351.9642	4.31036	214.537	15896.74	.0088
2038	60787.38	361.8192	4.31036	214.537	16076.81	.0085
2039	61516.82	371.9501	4.31036	214.537	16258.24	.0082
2040	62255.03	382.3647	4.31036	214.537	16441.81	.0079
2041	63002.09	393.0709	4.31036	214.537	16627.22	.0076
2042	63758.11	404.0769	4.31036	214.537	16814.65	.0073
2043	64523.21	415.3911	4.31036	214.537	17004.37	.007
2044	65297.49	427.022	4.31036	214.537	17195.47	.0067
2045	66081.06	438.9786	4.31036	214.537	17387.93	.0064
2046	66874.03	451.27	4.31036	214.537	17582.33	.0061
2047	67676.52	463.9056	4.31036	214.537	17777.72	.0058
2048	68488.64	476.895	4.31036	214.537	17975.72	.0055
2049	69310.5	490.248	4.31036	214.537	18175.11	.0052
2050	70142.23	503.9749	4.31036	214.537	18376.09	.0049

Table 7: Assumptions for each calendar year

Birth year	Normal Retirement Age	Delayed Retirement Credit
1890	780	.03
1891	780	.03
1892	780	.03
1893	780	.03
1894	780	.03
1895	780	.03
1896	780	.03
1897	780	.03
1898	780	.03
1899	780	.03
1900	780	.03
1901	780	.03
1902	780	.03
1903	780	.03
1904	780	.03
1905	780	.03
1906	780	.03
1907	780	.03
1908	780	.03
1909	780	.03
1910	780	.03
1911	780	.03
1912	780	.03
1913	780	.03
1914	780	.03
1915	780	.03
1916	780	.03
1917	780	.03
1918	780	.03
1919	780	.03
1920	780	.03
1921	780	.03
1922	780	.03
1923	780	.03
1924	780	.03
1925	780	.035
1926	780	.035
1927	780	.04
1928	780	.04
1929	780	.045
1930	780	.045
1931	780	.05
1932	780	.05
1933	780	.055
1934	780	.055
1935	780	.06
1936	780	.06
1937	780	.065
1938	782	.065
1939	784	.07
1940	786	.07
1941	788	.075
1942	790	.075
1943	792	.08
1944	792	.08
1945	792	.08
1946	792	.08
1947	792	.08
1948	792	.08
1949	792	.08
1950	792	.08
1951	792	.08
1952	792	.08
1953	792	.08
1954	792	.08
1955	794	.08
1956	796	.08
1957	798	.08
1958	800	.08
1959	802	.08
1960	804	.08

Table 8: Assumptions for each birth year. In years after 1960, all values are held constant at their 1960 levels.

	Coef.	P-value
Ever diagnosed with cancer	0.003	0.879
Ever diagnosed with diabetes	-0.036	0.064
Ever diagnosed with heart disease	-0.053*	0.003
Ever diagnosed with high blood pressure	0.001	0.960
Ever diagnosed with lung disease	-0.082	0.001
Ever diagnosed with stroke	-0.094*	0.000
Heart attack since last wave	0.011	0.802
One IADL limitation	-0.226*	0.000
Two or more IADL limitations	-0.321*	0.000
One ADL limitation	-0.247*	0.000
Two ADL limitations	-0.285*	0.000
Three or more ADL limitations	-0.270*	0.000
Current smoker	-0.015	0.373
Obese	-0.005	0.757
Single	-0.011	0.590
Widowed	-0.006	0.733
Mild cognitive impairment (lowest cogn. score observed)	-0.067	0.050
Dementia (lowest cogn. score observed)	-0.077*	0.000
Constant	0.865*	0.000
N	1,001	
Adjusted R^2	0.44	

* $p < 0.01$

Table 9: OLS regression of the predicted HUI3 score against chronic conditions, demographic characteristics, and FEM-type functional status specification. Data source: Health and Retirement Study, 2000. Sample included community respondents over age 50. ADL: Activities of Daily Living, IADL: Instrumental Activities of Daily Living.

	2000			2006			2012			2018		
	FEM	HRS		FEM	HRS		FEM	HRS		FEM	HRS	
Outcome	mean	mean	p	mean	mean	p	mean	mean	p	mean	mean	p
Died	0.054	0.050	0.175	0.067	0.074	0.076	0.087	0.083	0.356	0.122	0.106	0.010
Lives in nursing home	0.013	0.015	0.176	0.013	0.014	0.332	0.015	0.023	0.007	0.024	0.017	0.078

Table 10: Crossvalidation of 1998 cohort: Simulated vs reported mortality and nursing home outcomes in 2000, 2006, 2012, and 2018

	2000			2006			2012			2018		
	FEM	HRS		FEM	HRS		FEM	HRS		FEM	HRS	
Outcome	mean	mean	p	mean	mean	p	mean	mean	p	mean	mean	p
Age on July 1st	66.659	66.643	0.905	70.818	70.823	0.968	74.987	74.890	0.465	79.176	78.932	0.093
Black	0.082	0.080	0.576	0.082	0.080	0.664	0.081	0.080	0.883	0.079	0.082	0.665
Hispanic	0.057	0.055	0.548	0.059	0.056	0.439	0.061	0.063	0.645	0.062	0.062	0.973
Male	0.450	0.450	0.913	0.443	0.446	0.711	0.429	0.444	0.096	0.410	0.431	0.069

Table 11: Crossvalidation of 1998 cohort: Simulated vs reported demographic outcomes in 2000, 2006, 2012, and 2018

	2000			2006			2012			2018		
	FEM	HRS		FEM	HRS		FEM	HRS		FEM	HRS	
Outcome	mean	mean	p	mean	mean	p	mean	mean	p	mean	mean	p
Any ADLs	0.147	0.167	0.000	0.148	0.175	0.000	0.167	0.180	0.091	0.200	0.187	0.266
Any IADLs	0.111	0.126	0.001	0.112	0.132	0.000	0.132	0.157	0.000	0.163	0.153	0.323
Cancer	0.110	0.116	0.189	0.154	0.153	0.870	0.201	0.199	0.825	0.244	0.233	0.339
Diabetes	0.141	0.138	0.531	0.188	0.191	0.716	0.237	0.242	0.494	0.280	0.281	0.955
Heart Disease	0.190	0.195	0.393	0.227	0.245	0.008	0.279	0.290	0.247	0.337	0.355	0.187
Hypertension	0.448	0.444	0.543	0.556	0.563	0.360	0.639	0.652	0.162	0.708	0.699	0.478
Lung Disease	0.071	0.071	0.990	0.091	0.085	0.189	0.110	0.105	0.436	0.123	0.111	0.205
Stroke	0.060	0.065	0.136	0.071	0.083	0.006	0.090	0.105	0.010	0.115	0.120	0.583

Table 12: Crossvalidation of 1998 cohort: Simulated vs reported binary health outcomes in 2000, 2006, 2012, and 2018

	2000			2006			2012			2018		
	FEM	HRS		FEM	HRS		FEM	HRS		FEM	HRS	
Outcome	mean	mean	p	mean	mean	p	mean	mean	p	mean	mean	p
BMI	27.242	27.241	0.988	27.730	27.914	0.045	27.906	28.010	0.361	27.957	28.141	0.261
Ever smoked	0.592	0.596	0.549	0.569	0.580	0.165	0.531	0.551	0.037	0.476	0.488	0.399

Table 13: Crossvalidation of 1998 cohort: Simulated vs reported risk factor outcomes in 2000, 2006, 2012, and 2018

	2000			2006			2012			2018		
	FEM	HRS		FEM	HRS		FEM	HRS		FEM	HRS	
Outcome	mean	mean	p	mean	mean	p	mean	mean	p	mean	mean	p
Claiming DB pension	0.278	0.298	0.001	0.326	0.336	0.167	0.379	0.378	0.933	0.413	0.414	0.922
Claiming SSDI	0.054	0.054	0.921	0.061	0.099	0.000						
Claiming OASI	0.888	0.913	0.000	0.866	0.884	0.001	0.940	0.917	0.000	0.980	0.962	0.001
Claiming SSI	0.028	0.027	0.714	0.015	0.018	0.157	0.010	0.015	0.025	0.007	0.009	0.465
Working for pay	0.406	0.406	0.980	0.313	0.311	0.765	0.221	0.217	0.634	0.145	0.135	0.336

Table 14: Crossvalidation of 1998 cohort: Simulated vs reported binary economic outcomes in 2000, 2006, 2012, and 2018

	2000			2006			2012			2018		
	FEM	HRS		FEM	HRS		FEM	HRS		FEM	HRS	
Outcome	mean	mean	p	mean	mean	p	mean	mean	p	mean	mean	p
Earnings (thou.)	35.43	43.701	0	32.19	39.913	0	27.017	29.195	.2327	23.838	22.612	.65277

Table 15: Crossvalidation of 1998 cohort: Simulated vs reported continuous economic outcomes in 2000, 2006, 2012, and 2018

References

Feeny, D., Furlong, W., Boyle, M., and Torrance, G. W. (1995). Multi-attribute health status classification systems. *Pharmacoeconomics*, 7(6):490–502.

Feeny, D., Furlong, W., Torrance, G. W., Goldsmith, C. H., Zhu, Z., DePauw, S., Denton, M., and Boyle, M. (2002). Multiattribute and single-attribute utility functions for the health utilities index mark 3 system. *Medical care*, 40(2):113–128.

Feeny, D. H., Torrance, G. W., and Furlong, W. J. (1996). Health Utilities Index. In Spilker, B., editor, *Quality of Life and Pharmacoeconomics in Clinical Trials*, chapter 26, pages 239–252. Lippincott-Raven Press, Philadelphia, second edition.

Furlong, W., Feeny, D., Torrance, G. W., Goldsmith, C., DePauw, S., Boyle, M., Denton, M., and Zhu, Z. (1998). Multiplicative multi-attribute utility function for the Health Utilities Index Mark 3 (HUI3) system: a technical report. Technical Report 98-11, McMaster University Centre for Health Economics and Policy Analysis, Hamilton ON, Canada.

Furlong, W. J., Feeny, D. H., and Torrance, G. W. (2001). *HEALTH UTILITIES INDEX (HUI) procedures manual: algorithm for determining HUI Mark 3 (HUI3) health status classification levels, health states, health-related quality of life scores and single-attribute level utility scores from 33-item interviewer-administered health status questionnaires*. Health Utilities Inc., Dundas ON, Canada.

Goldman, D. P., Shekelle, P. G., Bhattacharya, J., Hurd, M., and Joyce, G. F. (2004). Health status and medical treatment of the future elderly. Technical report, DTIC Document.

Horsman, J., Furlong, W., Feeny, D., and Torrance, G. (2003). The Health Utilities Index (HUI®): concepts, measurement properties and applications. *Health and Quality of Life Outcomes*, 1(1):54.

Kapteyn, A., Michaud, P.-C., Smith, J. P., and Van Soest, A. (2006). Effects of attrition and non-response in the health and retirement study.

MacKinnon, J. G. and Magee, L. (1990). Transforming the dependent variable in regression models. *International Economic Review*, pages 315–339.

Roodman, D. (2011). Fitting fully observed recursive mixed-process models with cmp. *Stata Journal*, 11(2):159–206(48).

Selden, T. M. and Sing, M. (2008). Aligning the Medical Expenditure Panel Survey to aggregate us benchmarks. Technical report, Agency for Healthcare Research and Quality.
